# Multivariate genome-wide association analysis of dyslexia and quantitative reading skill improves gene discovery

**DOI:** 10.1038/s41398-025-03514-0

**Published:** 2025-08-18

**Authors:** Hayley S. Mountford, Else Eising, Pierre Fontanillas, Adam Auton, 23andMe Research Team, Adam Auton, Stella Aslibekyan, Robert K. Bell, Katelyn Kukar Bond, Zayn Cochinwala, Sayantan Das, Kahsaia de Brito, Emily DelloRusso, Chris Eijsbouts, Sarah L. Elson, Chris German, Julie M. Granka, Barry Hicks, David A. Hinds, Reza Jabal, Aly Khan, Matthew J. Kmiecik, Alan Kwong, Yanyu Liang, Keng-Han Lin, Matthew H. McIntyre, Shubham Saini, Anjali J. Shastri, Jingchunzi Shi, Suyash Shringarpure, Qiaojuan Jane Su, Vinh Tran, Joyce Y. Tung, Catherine H. Weldon, Wanwan Xu, Evan K. Irving-Pease, Catherine Doust, Timothy C. Bates, Nicholas G. Martin, Simon E. Fisher, Michelle Luciano

**Affiliations:** 1 School of Philosophy, Psychology and Language Sciences, University of Edinburgh, Edinburgh, UK; 2 Language and Genetics Department, Max Planck Institute for Psycholinguistics, Nijmegen, Netherlands; 3 23andMe, Inc., Sunnyvale, CA USA; 4 Section for Molecular Ecology and Evolution, Globe Institute, University of Copenhagen, Copenhagen, Denmark; 5 Lundbeck Foundation GeoGenetics Centre, Globe Institute, University of Copenhagen, Copenhagen, Denmark; 6 Genetic Epidemiology Laboratory, QIMR Berghofer Medical Research Institute, Herston, QLD Australia; 7 Donders Institute for Brain, Cognition and Behaviour, Radboud University, Nijmegen, Netherlands

**Keywords:** Molecular neuroscience, Genetics, Human behaviour

## Abstract

The ability to read is an important life skill and a major route to education. Dyslexia, characterized by difficulties with accurate/ fluent word reading, and poor spelling is influenced by genetic variation, with a twin study heritability estimate of 0.4–0.6. Until recently, genomic investigations were limited by modest sample size. We used a multivariate genome-wide association study (GWAS) method, MTAG, to leverage summary statistics from two independent GWAS efforts, boosting power for analyses of dyslexia; the GenLang meta-analysis of word reading (*N* = 27,180) and the 23andMe, Inc., study of dyslexia (N_cases_ = 51,800, N_controls_ = 1,087,070). We increased the effective sample size to 1,228,832 participants, representing the largest genetic study of reading-related phenotypes to date. Our analyses identified 80 independent genome-wide significant loci, including 36 regions which were not previously reported as significant. Of these 36 loci, 13 were novel regions with no prior association with dyslexia. We observed clear genetic correlations with cognitive and educational measures. Gene-set analyses revealed significant enrichment of dyslexia-associated genes in four neuronal biological process pathways, and findings were further supported by enrichment of neuronally expressed genes in the developing embryonic brain. Polygenic index analysis of our multivariate results predicted between 2.34–4.73% of variance in reading traits in an independent sample, the National Child Development Study cohort (*N* = 6410). Polygenic adaptation was examined using a large panel of ancient genomes spanning the last ~15 k years. We did not find evidence of selection, suggesting that dyslexia has not been subject to recent selection pressure in Europeans. By combining existing datasets to improve statistical power, these results provide novel insights into the biology of dyslexia.

## Introduction

Reading is a key academic skill and an important component of education. Difficulties with reading are associated with poorer life outcomes, lower socioeconomic status, and can greatly impact quality of life [1]. Dyslexia, characterized by difficulties with accurate and/or fluent word reading, and poor spelling, occurs in 5–10% of school age children [2]. Some diagnostic definitions of dyslexia extend to reduced performance on measures of verbal memory and processing speed, and/or emphasize a discrepancy between reading and other cognitive abilities [3]. Dyslexia tends to cluster within families [4] and shows high heritability in twin-studies (0.4–0.6) [5]. Similarly, quantitative measures of reading ability are highly heritable, with a recent twin-based meta-analysis reporting a heritability of 0.66 [6, 7], Unravelling the biological basis of dyslexia is essential in understanding difference in reading skill, and why some people struggle with reading throughout their lives.

Allelic variation in several genes have been associated with dyslexia and reading-related traits [5, 8–11], although with mixed support from replication studies [11]. Doust et al. (2022) [11] performed the largest genome-wide association study (GWAS) of this trait to-date, using 23andMe self-reported dyslexia diagnosis in 51,800 cases and 1,087,070 controls. Forty-two significantly associated regions were identified, including 27 not previously reported in studies of educational attainment or cognitive traits. The largest GWAS of quantitative reading skill [9] (meta-analysis of 33,959 individuals from 19 cohorts) by the GenLang Consortium identified a single locus associated with word reading (rs11208009, *P* = 1.10 × 10^−8^) containing three candidate genes (*DOCK7*, *ANGPTL3* and *USP1*). Strong genetic correlations were observed between quantitative measures of word reading (−0.71, 95% CI −0.62–−0.8), nonword reading (−0.7, 95% CI −0.61–−0.8), spelling (−0.75, 95% CI −0.64–−0.86) and dyslexia [11]. This is consistent with the view that dyslexia is representative of the low extreme of normal varying reading ability in the population [12, 13] rather than being a qualitatively distinct phenotype.

It is clear from studies of the genetics of dyslexia and reading [8, 9], echoed in other neurodevelopmental traits such as autism spectrum disorder (ASD) [14] and attention deficit hyperactivity disorder (ADHD) [15], that large sample sizes are key to improving resolution of associated variants. For developmental measures of literacy collected in childhood, it has historically been challenging to gather sufficient sample size. Similarly, availability of large cohorts with clinical diagnoses of dyslexia and suitable genetic data, are limited. One of the alternative methods to collecting and phenotyping new cohorts for a trait of interest, multi-trait analysis of GWAS approach (MTAG) [16], uses the shared genetic architecture of related phenotypes to increase gene discovery power. Grove and colleagues [14] used MTAG to increase their ASD GWAS power by adding GWAS for schizophrenia, educational attainment, and major depression. This showed stronger evidence for previously reported regions, and seven novel regions shared with educational attainment or depression. More recently, multivariate analyses were used across five psychiatric traits (ASD, ADHD, bipolar disorder, schizophrenia, and depression) [17], again increasing the number of associated loci identified for each individual trait, particularly bipolar disorder, which increased from 8 loci to 54. Given the strong genetic correlation between dyslexia and word-reading skills [11], we applied the multivariate method to boost sample size of the dyslexia and word reading GWAS, and identify novel associated loci.

## Methods and materials

### Multivariate GWAS

Multivariate GWAS (MTAG) [16] was performed using the dyslexia (23andMe, Inc, N_cases_ = 51,800, N_controls_ = 1,087,070) [11] and word reading (GenLang, *N* = 27,180) [9] GWAS summary statistics [16] without genomic control correction applied. Power estimation was calculated using the Genetic Power Calculator (https://zzz.bwh.harvard.edu/gpc/) [18] for quantitative traits and the Genetic Association Study (GAS) power calculator for binary traits (https://csg.sph.umich.edu/abecasis/cats/gas_power_calculator/). Associations were visualized using ggplot2 [19] and LocusZoom (http://locuszoom.org), and annotated using FUMA v1.5.0 [20]. SNP-based heritability $({h}_{{\rm{snp}}}^{2})$ was estimated using LDSC v1.0.1 [21] and SumHer (LDAK) [22]. Sample prevalence was estimated at 5% [11] and sample size of *N* = 1,228,832. Genetic correlations were performed using LD-Score v1.0.1 within the Complex-Traits Virtual Genetics Lab (CTG-VL) platform (https://vl.genoma.io) (Supplemental Method). Code generated for this study is available online (https://github.com/hayley-mountford/multivariate_GWAS_dyslexia).

### Biological annotation

Gene-based associations and gene-set biological pathways analyses were calculated using MAGMA v1.08 [23] within the FUMA interface (https://fuma.ctglab.nl/). Fine mapping and annotations were performed using the Variant Effect Predictor (VEP) online tool (http://grch37.ensembl.org/). Expression QTL analysis was performed using FUMA. MAGMA, within FUMA, was used to test for enrichment of tissue-specific annotations. To interrogate cell- and region-specific resolution, we accessed single-cell RNA-seq (scRNA) data via FUMA. Partitioned SNP heritability was examined using stratified LDSC, as described by Finucane et al. [24] (Supplemental Methods).

### Polygenic index prediction and selection

The dyslexia polygenic index (PGI) was calculated for the National Child Development Study (NCDS) (*N* = 6410) [25] on six longitudinal reading measures described in Bridges et al. [26] using both PRSice2 v2.3.5 [27] and SBayesRC v0.2.6 [28]. Evidence of polygenic selection for dyslexia was examined using a large panel of 1015 imputed ancient genomes from the last 15 k years, sampled from across West Eurasia [29, 30]. Allele frequency trajectories and selection coefficients were modelled using CLUES [31], then polygenic selection gradients with PALM [32] using imputed ancestral data described by Barrie et al. [33] (Supplemental Methods).

## Results

### Multivariate GWAS of dyslexia

The genetic correlation between the univariate summary statistics of dyslexia and word reading (Europeans only, without GC correction) was −0.71 (SE = 0.05, Z = −15.06), and indicative of a high degree of shared genetic etiology enabling multivariate GWAS analysis with MTAG [16]. Meta-analysis produced an equivalent sample size of 1,228,832 for dyslexia, using 5,449,985 SNPs shared between the univariate summary statistics. This provided more than 83% power to detect additive risk of up to 1.046 (5% prevalence) and ≥ 84% power to detect additive risk of 1.038 at 10% prevalence (*N* = 1,228,832, α = 5 × 10^−8^).

**Fig. 1:**
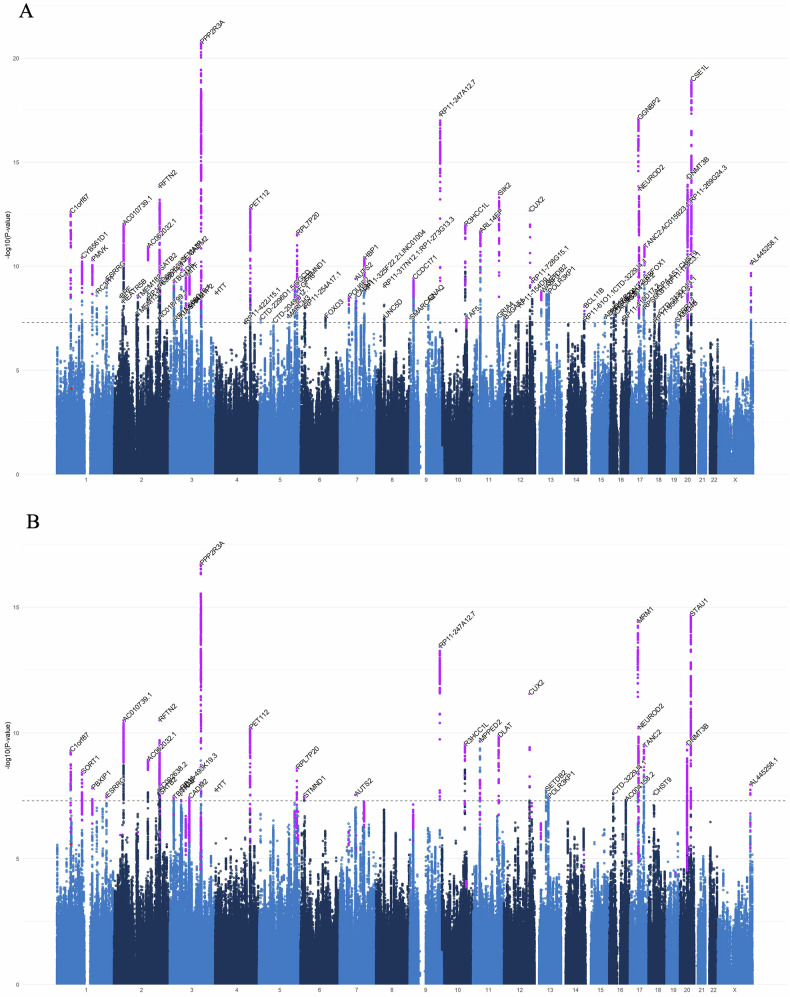
Regions associated with dyslexia and reading ability. Manhattan plot of the multivariate GWAS of **A** dyslexia and **B** reading ability. The y axis indicates the -log_10_ *P* value for association. The threshold for genome-wide significance (*P* < 5 × 10^−8^) is represented by a dashed grey line. Significant loci that were previously reported in the GenLang word reading GWAS [9] are represented in red, and those reported in the dyslexia GWAS [11] are shown in purple.

We identified 80 genome-wide significant (*P* ≤ 5 × 10^−8^) independent loci (*r*^*2*^ < 0.6, and < 250 kb maximum distance between LD blocks to merge into one genomic locus) (Fig. 1a, Table 1), containing 211 independent significant SNPs, independent from each other at an *R*^2^ of 0.1. Forty-four of these regions were previously reported as significantly associated with dyslexia; 41 in the univariate dyslexia GWAS [11], and three by Ciulkinyte et al. [34] who used genomic structural equation modelling (GenomicSEM) to identify pleiotropic loci for dyslexia and attention deficit disorder (ADHD). Of our 80 loci, 36 were not present in the literature. However, of these 80 identified loci, 66 were present in the uncorrected univariate dyslexia summary statistics available from 23andMe, Inc. for which genomic control correction was not applied (*P* ≤ 5 × 10^−08^). We therefore consider novel regions more conservatively; not reported as significant in Doust et al. [11], Ciulkinyte et al. [34] or in the uncorrected univariate summary statistics. We detected 36 previously unreported loci, and more conservatively, thirteen novel loci not previously associated at significance threshold with dyslexia (Tables 1 and 2).

**Table 1:** Loci associated with dyslexia detected by multivariate GWAS (MTAG), with the lead SNP indicated.

Cytoband	Lead SNP	Effect allele	Frequency	Effect (OR)	SE	MTAG P	Nearest Gene(s) to lead SNP	Doust et al. 2023	23andMe dyslexia uncorrected	Ciulkinyte et al. 2024
chr1p32.1	rs12723322	T	0.85	1.013	0.002	2.47 × 10^−13^	*[C1orf87]*	**rs12737449,** ***p*** = **1.40** **×** **10**^**−11**^	**rs12737449,** ***p*** = **2.40** **×** **10**^**−13**^	–
chr1p13.3	rs749056	T	0.69	1.009	0.001	3.55 × 10^−11^	*[CYB561D1]*	**rs2091329,** ***p*** = **1.90** **×** **10**^**−09**^	**rs2091329,** ***p*** = **7.50** **×** **10**^**−11**^	–
chr1q21.3	rs4845687	G	0.44	0.991	0.001	5.02 × 10^−11^	*KCNN3--[]--PMVK*	**rs4845687,** ***p*** = **1.10** **×** **10**^**−09**^	**rs4845687,** ***p*** = **3.79** **×** **10**^**−11**^	–
chr1q25.1	rs117256995	T	0.16	0.989	0.002	1.81 × 10^−09^	*[RC3H1]*	rs117256995, *p* = 9.70 × 10^−08^	**rs117256995,** ***p*** = **7.35** **×** **10**^**−09**^	–
chr1q41	rs17043436	G	0.84	0.989	0.002	4.26 × 10^−10^	*[ESRRG]*	**rs35570426,** ***p*** = **4.10** **×** **10**^**−08**^	**rs17043436,** ***p*** = **2.73** **×** **10**^**−09**^	–
chr2p23.2	rs1969131	T	0.17	1.01	0.002	2.99 × 10^−09^	*[BABAM2]*	**rs1969131,** ***p*** = **3.00** **×** **10**^**−08**^	**rs1969131,** ***p*** = **1.88** **×** **10**^**−09**^	–
chr2p22.2	rs2706809	T	0.46	1.008	0.001	5.20 × 10^−09^	*[HEATR5B]*	rs2287108, *p* = 1.30 × 10^−07^	**rs2287108,** ***p*** = **1.07** **×** **10**^**−08**^	–
chr2p22.1	rs906549	T	0.64	1.01	0.001	9.14 × 10^−13^	*SLC8A1---[]---C2orf91*	**rs906549,** ***p*** = **1.40** **×** **10**^**−09**^	**rs906549,** ***p*** = **5.06** **×** **10**^**−11**^	–
chr2q11.2	rs35783535	G	0.63	1.008	0.001	2.06 × 10^−08^	*TMEM131--[]--VWA3B*	rs11886417, *p* = 1.70 × 10^−07^	**rs11886417,** ***p*** = **1.44** **×** **10**^**−08**^	–
chr2q12.1	rs2540287	T	0.68	0.992	0.001	3.17 × 10^−09^	*[TMEM182]*	**rs367982014,** ***p*** = **1.80** **×** **10**^**−08**^	**rs2540287,** ***p*** = **2.53** **×** **10**^**−09**^	–
chr2q22.3	rs55683518	T	0.62	1.008	0.001	3.26 × 10^−09^	*ZEB2----[]----ACVR2A*	–	**rs497418,** ***p*** = **1.25** **×** **10**^**−10**^	–
chr2q22.3	rs497418	C	0.62	0.991	0.001	1.13 × 10^−11^	*ZEB2----[]---ACVR2A*	**rs497418,** ***p*** = **3.00** **×** **10**^**−09**^		–
chr2q32.3	rs719166	G	0.18	1.01	0.002	7.70 × 10^−10^	*TMEFF2----[]----SLC39A10*	rs719166, *p* = 1.50 × 10^−07^	**rs719166,** ***p*** = **1.18** **×** **10**^**−08**^	–
chr2q32.3	rs115653413	T	0.06	1.015	0.003	3.04 × 10^−08^	*TMEFF2----[]----SLC39A10*	rs115653413, *p* = 1.60 × 10^−07^		–
chr2q33.1	rs72916919	T	0.49	0.99	0.001	1.50 × 10^−14^	*[RFTN2]*	**rs72916919,** ***p*** = **4.10** **×** **10**^**−12**^	**rs72916919,** ***p*** = **5.50** **×** **10**^**−14**^	**rs7571545,** ***p*** = **1.58** **×** **10**^**−07**^
chr2q33.1	rs11692189	G	0.68	1.009	0.001	1.38 × 10^−10^	*PLCL1----[]-SATB2*	**rs6435017,** ***p*** = **4.90** **×** **10**^**−09**^	**rs6435017,** ***p*** = **2.23** **×** **10**^**−10**^	–
chr3p24.3	rs12493567	G	0.44	0.992	0.001	5.93 × 10^−10^	*[TBC1D5]*	**rs373178590,** ***p*** = **1.30** **×** **10**^**−09**^	**rs1374197,** ***p*** = **2.33** **×** **10**^**−09**^	–
chr3p24.3	rs79162559	G	0.92	1.013	0.002	3.84 × 10^−08^	*SGO1---[]----ZNF385D*	rs17805117, *p* = 6.20 × 10^−08^	**rs17805117,** ***p*** = **4.36** **×** **10**^**−09**^	rs7652099, *p* = 3.19 × 10^−05^
**chr3p21.31**	**rs140587615**	**T**	**0.14**	**0.99**	**0.002**	**2.52** **×** **10**^**−08**^	*ZNF501-[]-KIAA1143*	rs140587615, p = 5.40 × 10^−07^	rs140587615, *p* = 5.47 × 10^−08^	–
chr3p21.31	rs2624839	T	0.58	0.991	0.001	5.63 × 10^−11^	*[SEMA3F]*	**rs2624839,** ***p*** = **3.10** **×** **10**^**−09**^	**rs2624839,** ***p*** = **1.31** **×** **10**^**−10**^	**rs1005678,** ***p*** = **2.16** **×** **10**^**−08**^
chr3p13	rs6777363	T	0.58	1.008	0.001	2.57 × 10^−10^	*[MITF]*	**rs13097431,** ***p*** = **1.30** **×** **10**^**−09**^	**rs13097431,** ***p*** = **4.81** **×** **10**^**−11**^	–
chr3p12.1	rs35843490	T	0.51	1.007	0.001	9.77 × 10^−09^	*GBE1----[]----CADM2*	rs75197892, *p* = 1.10 × 10^−07^	**rs10511073,** ***p*** = **1.39** **×** **10**^**−11**^	rs79273178, *p* = 3.52 × 10^−05^
chr3p12.1	rs10511073	G	0.63	0.991	0.001	2.60 × 10^−11^	*[CADM2]*	**rs10511073,** ***p*** = **4.60** **×** **10**^**−10**^		**rs10511073,** ***p*** = **6.64** **×** **10**^**−08**^
chr3q22.3	rs13082684	G	0.76	0.986	0.002	1.80 × 10^−21^	*[PPP2R3A]*	**rs13082684,** ***p*** = **1.00** **×** **10**^**−16**^	**rs13082684,** ***p*** = **2.19** **×** **10**^**−19**^	**rs1403767,** ***p*** = **8.32** **×** **10**^**−11**^
**chr4p16.3**	**rs362307**	**T**	**0.07**	**1.015**	**0.002**	**1.98** **×** **10**^**−09**^	*[HTT]*	rs72239206, *p* = 3.40 × 10^−07^	rs362307, *p* = 5.84 × 10^−08^	–
**chr4q28.2**	**rs2952899**	**G**	**0.27**	**1.008**	**0.001**	**4.84** **×** **10**^**−08**^	*C4orf33---[]----PCDH10*	–	rs2952899, *p* = 1.71 × 10^−07^	rs7671307, *p* = 7.7 × 10^−05^
chr4q31.3	rs907237	T	0.51	0.991	0.001	1.65 × 10^−13^	*[GATB]*	rs4696277, *p* = 4.80 × 10^−11^	**rs4696277,** ***p*** = **9.89** **×** **10**^**−13**^	–
**chr5p15.31**	**rs17812877**	**T**	**0.8**	**1.009**	**0.002**	**3.21** **×** **10**^**−08**^	*TENT4A---[]---ADCY2*	–	rs536261263, *p* = 1.73 × 10^−07^	–
**chr5q12.1**	**rs12653108**	**G**	**0.69**	**0.992**	**0.001**	**4.72** **×** **10**^**−08**^	*IPO11---[]---HTR1A*	–	rs12653108, *p* = 6.90 × 10^−07^	–
**chr5q23.2**	**rs2055873**	**T**	**0.31**	**1.008**	**0.001**	**2.67** **×** **10**^**−08**^	*LMNB1--[]-MARCH3*	rs11749282, *p* = 9.10 × 10^−07^	rs11749282, *p* = 1.01 × 10^−07^	–
chr5q33.3	rs867009	G	0.36	1.008	0.001	7.45 × 10^−10^	*[SGCD]*	**rs867009,** ***p*** = **2.30** **×** **10**^**−08**^	**rs867009,** ***p*** = **1.36** **×** **10**^**−09**^	–
chr5q34	rs41012	C	0.25	0.99	0.001	2.77 × 10^−12^	*MAT2B----[]---TENM2*	**rs41012,** ***p*** = **1.30** **×** **10**^**−10**^	**rs41012,** ***p*** = **3.27** **×** **10**^**−12**^	–
chr5q35.1	rs3806929	G	0.62	1.008	0.001	1.31 × 10^−09^	*NPM1-[]--FGF18*	**rs59261790,** ***p*** = **7.20** **×** **10**^**−09**^	**rs62383978,** ***p*** = **1.66** **×** **10**^**−09**^	–
chr6p22.3	rs2876430	T	0.34	1.008	0.001	4.70 × 10^−10^	*ATXN1---[]--STMND1*	**rs2876430,** ***p*** = **3.70** **×** **10**^**−08**^	**rs2876430,** ***p*** = **2.37** **×** **10**^**−09**^	–
**chr6p22.3**	**rs7776042**	**T**	**0.15**	**1.011**	**0.002**	**8.88** **×** **10**^**−09**^	*RNF144B---[]---ID4*	rs2064081, *p* = 5.50 × 10^−07^	rs2064081, *p* = 5.65 × 10^−08^	–
chr6q21	rs2802295	G	0.62	0.993	0.001	2.63 × 10^−08^	*[FOXO3]*	rs11398842, *p* = 1.70 × 10^−07^	**rs6918436,** ***p*** = **3.31** **×** **10**^**−08**^	–
chr7p14.1	rs12673488	G	0.45	1.008	0.001	3.01 × 10^−09^	*[POU6F2]*	**rs62453457,** ***p*** = **3.30** **×** **10**^**−08**^	**rs62453457,** ***p*** = **2.07** **×** **10**^**−09**^	–
chr7q11.22	rs3735260	G	0.08	1.015	0.002	4.08 × 10^−10^	*[AUTS2]*	**rs3735260,** ***p*** = **4.70** **×** **10**^**−08**^	**rs3735260,** ***p*** = **3.14** **×** **10**^**−09**^	**rs73175930,** ***p*** = **7.16** **×** **10**^**−06**^
chr7q11.22	rs11770441	T	0.78	1.009	0.002	2.67 × 10^−09^	*[CALN1]*	**rs77059784,** ***p*** = **3.00** **×** **10**^**−08**^	**rs77059784,** ***p*** = **1.86** **×** **10**^**−09**^	–
chr7q22.3	rs6466034	T	0.61	0.992	0.001	1.48 × 10^−09^	*LHFPL3--[]--KMT2E*	rs7807853, *p* = 2.70 × 10^−07^	**rs7807853,** ***p*** = **3.59** **×** **10**^**−09**^	rs7776707, *p* = 2.52 × 10^−05^
chr7q22.3	rs75007274	G	0.78	0.99	0.002	4.06 × 10^−11^	*[HBP1]*	**rs3839821,** ***p*** = **2.80** **×** **10**^**−10**^	**rs112665932,** ***p*** = **1.91** **×** **10**^**−11**^	–
**chr8p12**	**rs79445414**	**T**	**0.96**	**0.98**	**0.003**	**9.22** **×** **10**^**−10**^	*DUSP26---[]----UNC5D*	rs79445414, *p* = 5.00 × 10^−08^	rs79445414, *p* = 3.40 × 10^−09^	rs117396993, *p* = 4.01 × 10^−06^
**chr8p12**	**rs56372812**	**T**	**0.9**	**0.988**	**0.002**	**2.64** **×** **10**^**−08**^	*DUSP26----[]--UNC5D*			
chr9p24.3	rs10964508	G	0.2	1.009	0.002	3.15 × 10^−08^	*[SMARCA2]*	rs10964508, *p* = 6.60 × 10^−07^	**rs10964508,** ***p*** = **6.93** **×** **10**^**−08**^	–
chr9p22.3	rs10756715	C	0.45	0.992	0.001	3.71 × 10^−10^	*[CCDC171]*	**rs3122702,** ***p*** = **8.30** **×** **10**^**−09**^	**rs3122702,** ***p*** = **4.14** **×** **10**^**−10**^	**rs1848582,** ***p*** = **9.68** **×** **10**^**−07**^
chr9q21.2	rs10869969	C	0.76	1.009	0.002	4.09 × 10^−09^	*[GNAQ]*	rs10869969, *p* = 5.80 × 10^−08^	**rs10869969,** ***p*** = **4.03** **×** **10**^**−09**^	**rs1246268,** ***p*** = **1.51** **×** **10**^**−06**^
chr9q34.11	rs9696811	T	0.31	0.988	0.001	5.38 × 10^−18^	*PTPA--[]--IER5L*	**rs9696811,** ***p*** = **1.00** **×** **10**^**−14**^	**rs9696811,** ***p*** = **4.92** **×** **10**^**−17**^	–
chr10q24.2	rs11189513	G	0.32	0.99	0.001	1.03 × 10^−12^	*[R3HCC1L]*	**rs10786387,** ***p*** = **1.10** **×** **10**^**−10**^	**rs10786387,** ***p*** = **2.67** **×** **10**^**−12**^	–
chr10q24.33	rs11191672	T	0.46	0.993	0.001	3.35 × 10^−08^	*[TAF5]*	**rs34732054** ***p*** = **3.70** **×** **10**^**−09**^	**rs11191672,** ***p*** = **7.82** **×** **10**^**−10**^	–
chr11p14.1	rs1765141	G	0.65	0.991	0.001	2.08 × 10^−12^	*ARL14EP-[]--MPPED2*	**rs676217** ***p*** = **1.10** **×** **10**^**−11**^	**rs676217,** ***p*** = **1.77** **×** **10**^**−13**^	–
**chr11q22.3**	**rs641574**	**G**	**0.38**	**0.993**	**0.001**	**2.70** **×** **10**^**−08**^	*[GRIA4]*	–	rs694514, *p* = 1.32 × 10^−07^	–
chr11q23.1	rs55945818	G	0.58	1.01	0.001	3.36 × 10^−14^	*[SIK2]*	**rs138127836** ***p*** = **1.70** **×** **10**^**−13**^	**rs55945818,** ***p*** = **1.13** **×** **10**^**−14**^	–
chr11q25	rs77867811	G	0.12	1.011	0.002	2.98 × 10^−08^	*B3GAT1--[]*	rs77867811 *p* = 2.00 × 10^−07^	**rs77867811,** ***p*** = **1.71** **×** **10**^**−08**^	–
chr12q14.1	rs57431669	T	0.21	1.009	0.002	8.51 × 10^−09^	*SLC16A7---[]----TAFA2*	rs57431669 *p* = 2.60 × 10^−07^	**rs57431669,** ***p*** = **2.30** **×** **10**^**−08**^	–
chr12q24.12	rs1265564	C	0.46	0.991	0.001	2.11 × 10^−13^	*[CUX2]*	**rs7310615** ***p*** = **1.10** **×** **10**^**−10**^	**rs7310615,** ***p*** = **2.66** **×** **10**^**−12**^	–
chr12q24.31	rs4767921	G	0.64	1.008	0.001	5.48 × 10^−10^	*POP5--[]-CABP1*	**rs4767921** ***p*** = **2.10** **×** **10**^**−08**^	**rs4767921,** ***p*** = **1.24** **×** **10**^**−09**^	–
chr13q12.13	rs9507729	G	0.74	1.009	0.001	1.63 × 10^−09^	*CDK8---[]--WASF3*	**rs375018025** ***p*** = **5.60** **×** **10**^**−09**^	**rs9507729,** ***p*** = **1.23** **×** **10**^**−09**^	–
chr13q14.2	rs4142285	G	0.3	1.009	0.001	7.17 × 10^−10^	*[SETDB2]*	rs7328782 *p* = 1.10 × 10^−07^	**rs7328782,** ***p*** = **8.10** **×** **10**^**−09**^	–
chr13q21.2	rs9538299	G	0.31	1.008	0.001	1.77 × 10^−09^	*PCDH17----[]---DIAPH3*	rs9538299 *p* = 2.60 × 10^−07^	**rs9538299,** ***p*** = **2.36** **×** **10**^**−08**^	–
chr14q32.2	rs10151901	G	0.79	0.991	0.002	3.97 × 10^−08^	*VRK1---[]---BCL11B*	rs7160112 *p* = 8.80 × 10^−07^	rs7160112, *p* = 9.65 × 10^−08^	**rs1246268,** ***p*** = **1.51** **×** **10**^**−06**^
chr14q32.2	rs7146019	G	0.58	0.993	0.001	8.74 × 10^−09^	*[BCL11B]*	**rs35131341** ***p*** = **6.00** **×** **10**^**−09**^	**rs2748809,** ***p*** = **9.57** **×** **10**^**−10**^	–
**chr15q25.1**	**rs60567504**	**G**	**0.73**	**1.008**	**0.001**	**2.86** **×** **10**^**−08**^	*[ABHD17C]*	rs71455352 *p* = 7.30 × 10^−07^	rs60567504, *p* = 1.25 × 10^−07^	rs60567504, *p* = 1.4 × 10^−05^
chr16p13.3	rs1639161	G	0.51	1.007	0.001	1.66 × 10^−08^	*[RBFOX1]*	rs565248640 *p* = 1.00 × 10^−07^	**rs1639161,** ***p*** = **2.19** **×** **10**^**−08**^	–
chr16p12.3	rs6498749	G	0.37	0.992	0.001	1.53 × 10^−09^	*XYLT1---[]---NPIPA8*	rs6498749 *p* = 2.50 × 10^−07^	**rs6498749,** ***p*** = **2.23** **×** **10**^**−08**^	**rs6498749,** ***p*** = **2.04** **×** **10**^**−06**^
**chr16p12.1**	**rs7184217**	**T**	**0.51**	**1.007**	**0.001**	**1.74** **×** **10**^**−08**^	*[CACNG3]*	–	rs7184217, *p* = 1.53 × 10^−07^	–
chr16p12.1	rs6497931	T	0.5	0.993	0.001	1.10 × 10^−08^	*HS3ST4--[]---C16orf82*	rs8057487 *p* = 9.60 × 10^−08^	**rs8057487,** ***p*** = **7.21** **×** **10**^**−09**^	–
chr16q21	rs62066820	T	0.01	0.971	0.005	3.58 × 10^−08^	*GOT2---[]----CDH8*	rs188134315 *p* = 4.70 × 10^−07^	**rs188134315,** ***p*** = **4.69** **×** **10**^**−08**^	–
chr16q22.2	rs11646282	T	0.16	0.99	0.002	4.64 × 10^−09^	*PMFBP1---[]---ZFHX3*	rs11646282 *p* = 4.50 × 10^−07^	**rs11646282,** ***p*** = **4.42** **×** **10**^**−08**^	–
chr17q12	rs12150665	T	0.59	1.011	0.001	7.88 × 10^−18^	*[GGNBP2]*	**rs34349354** ***p*** = **8.20** **×** **10**^**−15**^	**rs9906189,** ***p*** = **2.71** **×** **10**^**−16**^	–
chr17q12	rs12453682	T	0.68	1.011	0.001	2.03 × 10^−14^	*NEUROD2-[]--PPP1R1B*	**rs12453682** ***p*** = **2.90** **×** **10**^**−12**^	**rs12453682,** ***p*** = **3.71** **×** **10**^**−14**^	–
chr17q23.1	rs7219089	T	0.19	1.009	0.002	1.03 × 10^−08^	*[RNFT1]*	rs3066247 *p* = 2.80 × 10^−07^	**rs7219089,** ***p*** = **3.75** **×** **10**^**−08**^	–
chr17q23.3	rs113815022	T	0.23	0.99	0.002	1.19 × 10^−11^	*[TANC2]*	**rs72841395** ***p*** = **5.40** **×** **10**^**−09**^	**rs72841395,** ***p*** = **2.48** **×** **10**^**−10**^	–
**chr18q11.2**	**rs5022348**	**T**	**0.58**	**1.007**	**0.001**	**4.58** **×** **10**^**−08**^	*HRH4---[]-ZNF521*	–	rs1013987, *p* = 1.14 × 10^−06^	rs8089996, *p* = 9.76 × 10^−05^
chr18q11.2	rs7505854	G	0.47	1.008	0.001	1.82 × 10^−09^	*[CHST9]*	rs7505854 *p* = 3.20 × 10^−07^	**rs7505854,** ***p*** = **2.96** **×** **10**^**−08**^	–
chr18q21.1	rs12965648	C	0.57	1.007	0.001	1.74 × 10^−08^	*SKOR2--[]---SMAD2*	rs16950864 *p* = 1.10 × 10^−07^	**rs16950864,** ***p*** = **8.56** **×** **10**^**−09**^	–
chr19q13.2	rs30458	G	0.11	0.989	0.002	4.36 × 10^−08^	*[SAMD4B]*	**rs60963584** ***p*** = **2.70** **×** **10**^**−08**^	**rs30458,** ***p*** = **2.71** **×** **10**^**−09**^	–
chr19q13.33	rs112205523	T	0.31	0.992	0.001	2.23 × 10^−08^	*[PRR12]*	rs7508601 *p* = 1.10 × 10^−07^	**rs7508601,** ***p*** = **8.11** **×** **10**^**−09**^	–
chr20q11.21	rs4911257	T	0.61	0.99	0.001	5.56 × 10^−15^	*[DNMT3B]*	**rs4911257** ***p*** = **7.50** **×** **10**^**−14**^	**rs4911257,** ***p*** = **5.04** **×** **10**^**−16**^	**rs6061195,** ***p*** = **1.02** **×** **10**^**−06**^
chr20q13.13	rs2426117	G	0.68	1.013	0.001	1.15 × 10^−19^	*[CSE1L]*	**rs11393101** ***p*** = **2.20** **×** **10**^**−16**^	**rs6019624,** ***p*** = **1.00** **×** **10**^**−18**^	–
chrXq27.3	rs6626462	T	0.35	0.991	0.001	7.29 × 10^−11^	*TMEM257--[]---CXorf51B*	**rs5904158** ***p*** = **3.30** **×** **10**^**−09**^	**rs6626462,** ***p*** = **2.12** **×** **10**^**−10**^	–

Gene context is represented by [x], indicating the lead SNP is located within gene x; x-[]-y indicates the lead SNP is located in the intergenic region between gene x and gene y, and the number of dashes in the intergenic region represents the genomic distance as follows: no dash < 1000 bp; - < 10,000 bp; -- < 100,000 bp, --- < 1000,000 bp; ---- > 10,000,000 bp. Where loci overlap with regions present in Doust et al. [11], uncorrected dyslexia summary statistics, or Ciulkinyte et al. [34], the lead SNP and *P*-value, and significant regions (*P* ≤ 5.08 × 10^−06^ for Doust [11] and uncorrected, and *P* ≤ 3.08 × 10^*−*6^ for Ciulkinyte [34]) are indicated in bold. Dash indicates the region did not meet suggestive significance. Novel regions associated with dyslexia are indicated in bold.

**Table 2:** Regions not previously associated with dyslexia from multivariate GWAS using MTAG (i.e. regions that were not significant in Doust et al. [11] or Ciulkinyte et al. [34]).

Associated region	Lead SNP	Other Independent significant SNPs	*P*	Number of SNPs	Number of significant SNPs	Nearest gene(s) to lead SNP	Known GWAS associations (*p* ≤ 5 × 10^−8^) for lead SNP	Known GWAS associations (*p* ≤ 5 × 10^−8^) for candidate SNPs in LD with independent significant SNPs
chr1:173746659–174941727	rs117256995	rs7553539	1.81 × 10^−09^	33	26	*RC3H1*	None	Low density lipoprotein cholesterol levels
chr2:37063240–37278206	rs2706809	–	5.20 × 10^−09^	78	57	*HEATR5B*	None	Personality or cognitive traits, cortical surface area, dorsolateral prefrontal thickness, subcortical volume, node-level brain connectivity, brain morphology, brain region volumes, whole brain free water diffusion, schizophrenia, alanine transaminase levels, gamma glutamyl transpeptidase, red cell distribution width, C-reactive protein, keratinocyte cancer, heart failure
chr2:98325330–98626286	rs35783535	rs11886417	2.06 × 10^−08^	260	176	*TMEM131, VWA3B*	Acute myeloid leukemia	Insomnia, acute myeloid leukemia, diastolic blood pressure, milk oligosaccharide concentration, mean spheric corpuscular volume, neutrophil percentage of white cells, eosinophil counts
chr2:147356975–147525485	rs55683518	–	3.26 × 10^−09^	121	59	*ZEB2, ACVR2A*	None	Insomnia
chr2:193965614–194320930	rs719166	–	7.70 × 10^−10^	15	11	*TMEFF2, SLC39A10*	None	None
chr2:194986654–195250719	rs115653413	–	3.04 × 10^−08^	9	4	*TMEFF2, SLC39A10*	None	None
chr3:20402881–20565077	rs79162559	–	3.84 × 10^−08^	63	54	*SGO1, ZNF385D*	None	None
**chr3:44370851–44780449**	**rs140587615**	**–**	**2.52** **×** **10**^**−08**^	**94**	**62**	***ZNF501, KIAA1143***	None	Daytime nap
chr3:83571268–84334893	rs35843490	rs143828159	9.77 × 10^−09^	273	181	*GBE1, CADM2*	None	Body mass index, F-fish liking (derived food-liking factor)
**chr4:3142660–3273010**	**rs362307**	**–**	**1.98** **×** **10**^**−09**^	**6**	**3**	***HTT***	Educational attainment, household income, brain morphology, drinks per week, worry/ vulnerability (factor of neuroticism), worry too long after and embarrassing experience, automobile speeding propensity, walking pace, type 2 diabetes, high density lipoprotein cholesterol levels, predicted visceral adipose tissue	Educational attainment, household income, brain morphology, drinks per week, worry/ vulnerability (factor of neuroticism), worry too long after and embarrassing experience, automobile speeding propensity, walking pace, type 2 diabetes, high density lipoprotein cholesterol levels, predicted visceral adipose tissue
**chr4:130740635–130950008**	**rs2952899**	**–**	**4.84** **×** **10**^**−08**^	**258**	**147**	***C4orf33, PCDH10***	None	Insomnia, chronotype, type 2 diabetes, body mass index, weight, adult body size
**chr5:7367049–7440086**	**rs17812877**	**–**	**3.21** **×** **10**^**−08**^	**36**	**19**	***TENT4A, ADCY2***	None	Educational attainment, highest math class taken, cognitive performance, insomnia
**chr5:62606583–62976937**	**rs12653108**	**–**	**4.72** **×** **10**^**−08**^	**203**	**125**	***IPO11, HTR1A***	None	Attention deficit hyperactivity disorder or autism spectrum disorder or intelligence (pleiotropy), cognitive performance, intelligence, insomnia, smoking initiation, leisure screen time, leisure sedentary behavior (television watching), body mass index
**chr5:126200835–126201232**	**rs2055873**	**–**	**2.67** **×** **10**^**−08**^	**3**	**2**	***LMNB1, MARCH3***	None	None
**chr6:19033780–19207129**	**rs7776042**	**–**	**8.88** **×** **10**^**−09**^	**59**	**43**	***RNF144B, ID4***	None	None
chr6:108861264–109005588	rs2802295	–	2.63 × 10^−08^	41	34	*FOXO3*	Cortical surface area, body mass index	Attention deficit hyperactivity disorder or autism spectrum disorder or intelligence (pleiotropy), intelligence, educational attainment, self-reported math ability, reaction time, schizophrenia, brain morphology, brain region volumes, subcortical volumes, brainstem volume, cortical thickness, cortical surface area, total PHF-tau, node-level brain connectivity, ganglion cell inner plexiform layer, macular thickness, retinal nerve fiber thickness, coffee consumption, gamma glutamyl transpeptidase, creatinine levels, vigorous physical activity, body mass index, waist-hip ratio, hip circumference, weight, fat-free mass, predicted visceral adipose tissue, systolic blood pressure, height, lung function, IGF-1 and IGF-3 levels, pulse pressure, platelet distribution
chr7:104579775–105031108	rs6466034	–	1.48 × 10^−09^	277	190	*LHFPL3, KMT2E*	None	Anorexia nervosa or attention-deficit/hyperactivity disorder or autism spectrum disorder or bipolar disorder or major depression or obsessive-compulsive disorder or schizophrenia or Tourette syndrome (pleiotropy), autism spectrum disorder or schizophrenia (pleiotropy), schizophrenia, general cognitive ability, household income, smoking cessation, general risk tolerance, height, biological sex, sunburns, serum 25-hydroxyvitamin D levels, peak expiratory flow, lung function, waist circumference, waist-hip-ratio, age-related macular degeneration, estimated glomerular filtration rate
**chr8:33604120–34678632**	**rs79445414**	**–**	**9.22** **×** **10**^**−10**^	**25**	**18**	***DUSP26, UNC5D***	Schizophrenia	Anorexia nervosa, attention-deficit/hyperactivity disorder, autism spectrum disorder, bipolar disorder, major depression, obsessive-compulsive disorder, schizophrenia, or Tourette syndrome (pleiotropy), attention deficit hyperactivity disorder or cannabis use, attention deficit disorder, major depressive disorder vs ADHD, bipolar disorder, schizophrenia
**chr8:35010162–35315188**	**rs56372812**	**–**	**2.64** **×** **10**^**−08**^	**83**	**44**	***DUSP26, UNC5D***	None	Insomnia, adolescent idiopathic scoliosis, height
chr9:2030300–2037264	rs10964508	–	3.15 × 10^−08^	11	9	*SMARCA2*	None	None
**chr11:105729068–105877624**	**rs641574**	**rs583452**	**2.70** **×** **10**^**−08**^	**115**	**81**	***GRIA4***	Cognitive performance	Educational attainment, highest math class taken, self-reported math ability, age when finished full-time education, cognitive performance, intelligence
chr11:134261150–134294813	rs77867811	–	2.98 × 10^−08^	64	55	*B3GAT1*	None	Insomnia, galactosylgalactosylxylosylprotein 3-beta-glucuronosyltransferase 1 level in chronic kidney disease with hypertension and no diabetes, serum alkaline phosphatase levels, N-glycan levels, transferrin N-glycan 21 levels, transferrin N-glycan 33 levels, transferrin N-glycan 35 levels, liver enzyme levels (alkaline phosphatase), prostate cancer
chr12:60861783–61207322	rs57431669	rs11173526	8.51 × 10^−09^	241	163	*SLC16A7, TAFA2*	None	Educational attainment (years of education), noncognitive aspects of educational attainment, adventurousness, lung function
chr13:50002976–50101159	rs4142285	rs7327520	7.17 × 10^−10^	148	101	*SETDB2*	None	Mean spheric corpuscular volume, height, AFP levels, lung function
chr13:59170846–59706143	rs9538299	rs944875; rs9569898	1.77 × 10^−09^	188	122	*PCDH17, DIAPH3*	None	Intelligence, externalizing behavior, chronotype, age and first sexual intercourse
**chr15:80993570–81061697**	**rs60567504**	**–**	**2.86** **×** **10**^**−08**^	**24**	**14**	***ABHD17C***	None	Externalizing behavior, smoking initiation, migraine, body mass index, waist-hip ratio
chr16:6524773–6566626	rs1639161	–	1.66 × 10^−08^	27	21	*RBFOX1*	None	Insomnia, sleep duration, chronic obstructive pulmonary disease liability
**chr16:24353071–24355216**	**rs7184217**	**–**	**1.74** **×** **10**^**−08**^	**2**	**2**	***CACNG3***	None	None
chr16:26152155–26199501	rs6497931	–	1.10 × 10^−08^	31	19	*HS3ST4, C16orf82*	None	Educational attainment
chr16:58684165–58872564	rs62066820	–	3.58 × 10^−08^	8	5	*GOT2, CDH8*	None	None
chr16:72333576–72697419	rs11646282	–	4.64 × 10^−09^	231	150	*PMFBP1, ZFHX3*	None	Intelligence, smoking initiation, height, neck pain or should pain, multi-trait sex score
chr17:57796677–58046246	rs7219089	–	1.03 × 10^−08^	102	63	*RNFT1*	None	Chronic inflammatory diseases (pleiotropy), citrate levels, platelet count, eosinophil percentage of granulocytes, triglyceride levels, hematocrit, high density lipoprotein cholesterol levels, low density lipoprotein cholesterol levels, total bilirubin levels, glycated hemoglobin levels, C-reactive protein levels, serum phosphate levels, red cell distribution width, red blood cell count,
**chr18:22618399–22648505**	**rs5022348**	**–**	**4.58** **×** **10**^**−08**^	**15**	**10**	***HRH4, ZNF521***	None	Educational attainment, chronotype, morningness, smoking initiation, age at first sexual intercourse
chr18:24627620–24757543	rs7505854	rs12956646	1.82 × 10^−09^	70	44	*CHST9*	None	None
chr18:44817871–44932591	rs12965648	–	1.74 × 10^−08^	102	65	*SKOR2, SMAD2*	None	None
chr19:50105239–50164994	rs112205523	–	2.23 × 10^−08^	24	10	*PRR12*	None	Schizophrenia, autoimmune thyroid disease

Novel regions, not significant in Doust et al. [11], the uncorrected dyslexia summary statistics or in Ciulkinyte et al. [34], are indicated in bold. Table shows associated region, lead SNP and *P*-value, number of independent and total number of significant SNPs in the region, and the closest gene to the lead SNP. Known GWAS associations reported in GWAS catalogue at *P* ≤ 5 × 10^−8^ are reported for the lead SNP and for candidate SNPs in LD with independent significant SNPs.

Quantile-Quantile (Q-Q) plots (Figure S1) indicated appropriate control for population stratification, as markers showing low association with dyslexia did not deviate from the expected quantile. Moderate genomic inflation was observed (λ = 1.573, χ^2^ = 1.773, LD intercept = 1.017 (0.012)) and consistent with a highly polygenic trait (see Supplementary Note for further discussion). Summary statistics for SNPs reaching suggestive significance (*P* ≤ 1 × 10^−5^) are presented in Table S1.

The most significantly associated loci were consistent with regions reported in the univariate dyslexia GWAS [11], with each showing higher significance in the multivariate analysis. The top locus, chr3q22.2 (rs13082684, *P* = 1.8 × 10^−21^) containing *PPP2R3A* (Table 1) is consistent with the most highly associated SNP of the dyslexia GWAS. The second top SNP in the present study, rs2426117 (*P* = 1 × 10^−18^) mapped in region chr20q13.13 mapped to within the gene *CSE1L*, where previously it was rs6019624 (*P* = 2.2 × 10^−16^) in neighboring gene *ARFGEF2*. The third top SNP, rs9696811 (*P* = 5.38 × 10^−18^) was located in region chr9q34.11, showing consistently higher significance than in the prior study (Table 1).

The lead SNP identified by the GenLang word-reading meta-GWAS [9], rs11208009, did not reach genome-wide significance in the present multivariate study (*P* = 7.85 × 10^−5^). However, it fell within a region that reached suggestive significance (chr1:62900811–63199936) at *P* = 1.9 × 10^−6^ in which rs1168114 (LD = 0.636) was now the lead SNP. This locus overlaps completely with the original study, therefore including candidate genes *DOCK7*, *ANGPTL3*, and *USP1* (Figure S2).

### Novel regions associated with dyslexia

Our multivariate analysis detected 36 regions not previously reported as significantly associated with dyslexia [11, 34] (Table 2). Individual LocusZoom plots for these 36 regions are presented in Figures S3–S38. More conservatively, we detected 13 regions that did not previously reach genome-wide significance in the univariate or the uncorrected dyslexia summary statistics [11] or the more recent GenomicSEM study [34] (shown in bold in Table 2).

Prior associations were reported for three novel lead-SNPs: the most significantly associated novel SNP, rs79445414 (*P* = 9.22 × 10^−10^) with schizophrenia [35], rs583452 (*P* = 2.7 × 10^−08^) in gene *GRIA4* with cognitive performance, and rs362307 (*P* = 1.98 × 10^−09^) within the gene *HTT*, previously linked to a range of phenotypes including educational attainment [36], cognitive ability [37], and a “worry” phenotype key to neuroticism [38, 39]. No associations were reported in the GWAS Catalog for three of the novel loci, with lead SNPs rs2055873 mapped to *LMNB1* and *MARCH3*, rs7776042 mapped to *RNF144B* and *ID4*, and rs7184217 in gene *CACNG3*. Associations of SNPs in LD with significant SNPs in the regions showed associations with several cognitive, psychiatric and neurodevelopmental phenotypes, particularly ADHD [40, 41].

### Multivariate GWAS of reading ability

We also examined the reading ability output of MTAG, resulting in an effective sample size of *N* = 102,082, providing 87% power to detect additive trait variance of up to 0.04% (α = 5 × 10^−8^). Thirty-five independent loci met the genome-wide significance threshold (Fig. 1b). Of these 35 associated loci, 28 were genome-wide significant in the original univariate dyslexia GWAS [11], and 34 were significant in the uncorrected dyslexia summary statistics. The novel locus present in the reading ability multivariate GWAS (rs362307 (*P* = 1.91 × 10^−8^) in *HTT*) was also novel in the dyslexia multivariate analysis. Summary statistics for SNPs reaching suggestive significance (*P* ≤ 1 × 10^−5^) are presented in Table S2 and regions significantly associated with the reading ability multivariate analysis are presented in Table S3.

The GenLang word-reading meta-GWAS lead-SNP [9], rs11208009, did not reach genome-wide significance in the present multivariate study (*P* = 2.71 × 10^−6^). However, it fell within a region of suggestive significance (chr1:62900811–63199936) in which rs1168114 (LD = 0.636) was now the lead SNP (*P* = 1.96 × 10^−7^), fully overlapping with the original study containing genes *DOCK7*, *ANGPTL3*, and *USP1*.

Q-Q plots (Figure S39) indicated appropriate control for population stratification. Genomic inflation suggested that moderate population stratification was present (λ = 1.28, χ^2^ = 1.431), however the low LD intercept (0.843 (0.01) indicated that the discrepancy in sample size affects the reliability of results for reading ability (see Supplementary Note for further discussion). Therefore, subsequent genetic and biological analyses are focused only on the dyslexia multivariate summary statistics.

### Heritability and genetic correlations of dyslexia

LDSC analysis of the dyslexia multivariate GWAS revealed a liability-scale SNP-based heritability estimate $({h}_{{\rm{snp}}}^{2})$ of 0.129 (SE = 0.005, 95% CI 0.12–0.139) at 5% population prevalence of dyslexia, ${h}_{{\rm{snp}}}^{2}=0.143$ (SE = 0.006, 95% CI 0.131–0.155) at 7%, and ${h}_{{\rm{snp}}}^{2}=0.160$ (SE = 0.006, 95% CI 0.148–0.173) at 10%. LDSC is known to overestimate SNP-based heritability because it assumes that each SNP contributes equal heritability, so we also applied SumHer which expects heritability to vary with both linkage disequilibrium and minor allele frequency [22]. SumHer liability-scale ${h}_{{\rm{snp}}}^{2}$ estimates for the dyslexia multivariate GWAS were ${h}_{{\rm{snp}}}^{2}=0.164$ (SE = 0.005, 95% CI 0.154–0.174) at 5% population prevalence of dyslexia, ${h}_{{\rm{snp}}}^{2}=0.192$ (SE = 0.006, 95% CI 0.18–0.204) at 7%, and ${h}_{{\rm{snp}}}^{2}=0.204$ (SE = 0.006, 95% CI 0.191–0.216) at 10%.

**Fig. 2:**
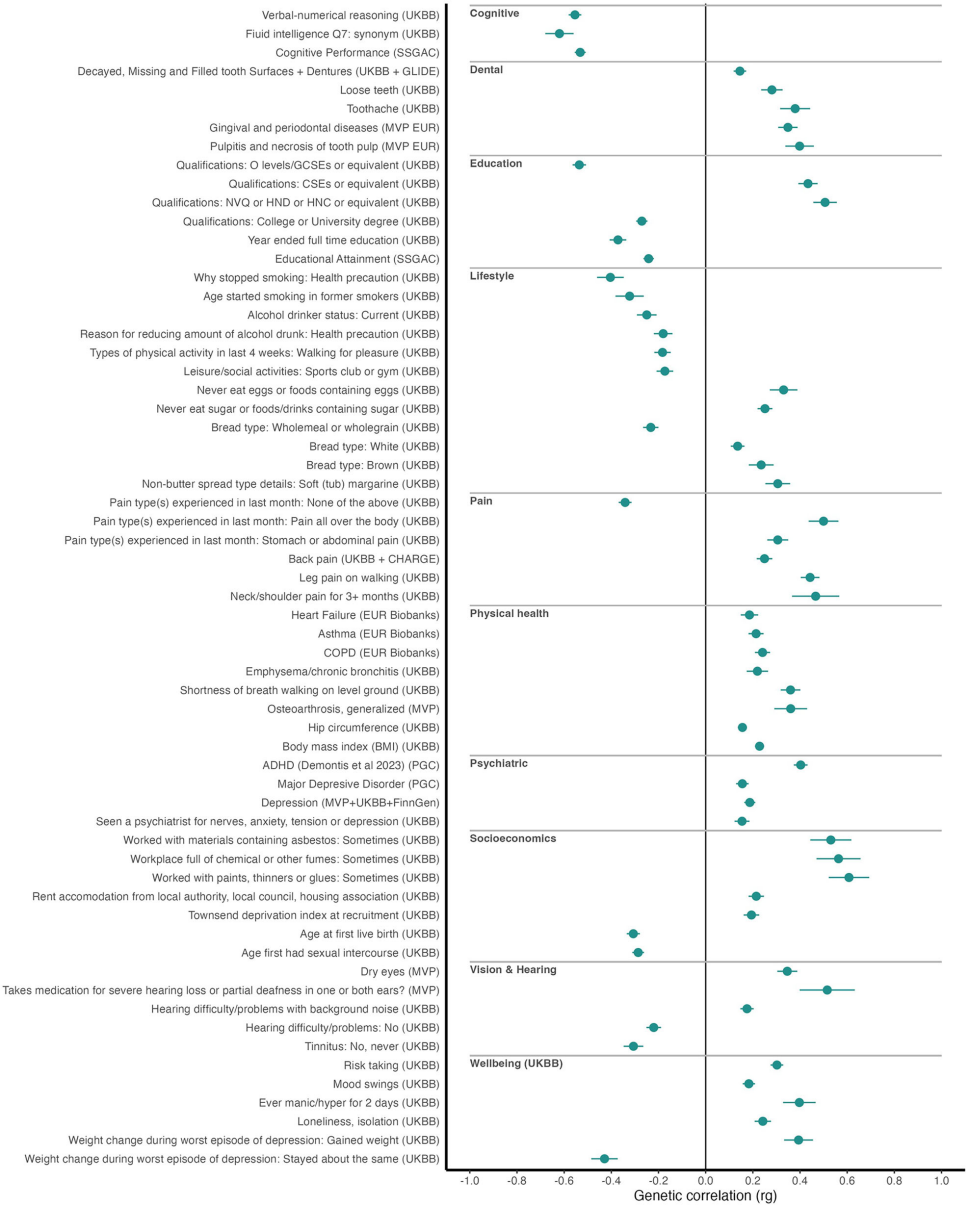
Genetic correlations of dyslexia with selected relevant phenotypes. Significant (*P* ≤ 1.77 × 10^−5^) genetic correlations (r_g_) between multivariate analysis of dyslexia and other selected phenotypes. UKBB UK Biobank, MVP Million Veterans Program, MVP EUR Million Veterans Program Europeans, GLIDE Gene-Lifestyle Interactions in Dental Endpoints, SSGAC Social Science Genetic Association Consortium, PGC Psychiatric Genetics Consortium.

Genetic correlations were estimated between multivariate dyslexia and 2824 traits (Bonferroni corrected threshold of *P* ≤ 1.77 × 10^−5^) including recently published summary statistics for ASD [14] and ADHD [15]. Statistically significant genetic correlations (*P* ≤ 1.77 × 10^−5^) were found for 489 traits. A subset of relevant significant correlations is presented in Fig. 2, with full results in Table S4.

Dyslexia showed strongly negative correlations (*r*_g_) with measures of intelligence, specifically verbal-numerical reasoning (UK Biobank) (−0.56, SE = 0.03) in which the F17 synonym question was most strongly correlated (−0.62, SE = 0.06), and cognitive performance (−0.53, SE = 0.02) (SSGAC). Consistent with previous findings, dyslexia was strongly genetically correlated with academic achievement and education level, including highest qualification of GCSEs or equivalent (−0.52, SE = 0.04), years in full time education (−0.37, SE = 0.04), completing college or a university degree (−0.27, SE = 0.02) (UK Biobank) and educational attainment (−0.24, SE = 0.02) (SSGAC). Dyslexia showed positive genetic correlations with workplace hazards (exposure to paints, thinners or glues (0.61, SE = 0.09); chemicals or fumes (0.56, SE = 0.09); asbestos (0.53, SE = 0.09); workplace dust (0.53, SE = 0.1)) (UK Biobank), and achieving either vocational qualifications (NVQ or HND or HNC: 0.51, SE = 0.05; CSEs or equivalent: 0.43, SE = 0.04) (UK Biobank).

In terms of neurodevelopmental traits, ADHD [15] showed a positive correlation with dyslexia (0.4, SE = 0.03). This was weaker than that shown by the univariate dyslexia study alone (0.53, SE = 0.12) [11], and while not reported by Eising et al. [9], we calculated *r*_*g*_ for ADHD and the univariate word-reading as −0.40 (SE = 0.05). ASD was not significantly correlated (0.09, SE = 0.04, *P* = 0.02) with dyslexia.

Major depressive disorder showed a positive correlation (0.16, SE = 0.03) (Psychiatric Genetics Consortium), as did measures linked to poorer wellbeing including manic episodes (0.4, SE = 0.07), risk taking (0.3, SE = 0.03) and mood swings (0.18, SE = 0.03). Measures of pain, injury, and use of pain medication were also highly correlated, including taking medication for severe hearing loss or deafness (0.52, SE = 0.12) (Million Veterans Program), pain all over the body (0.5, SE = 0.06), neck and should pain (0.47, SE = 0.1) and leg pain on walking (0.44, SE = 0.04) (UK Biobank).

### Gene and gene-set associations

Gene-based analysis of the multivariate dyslexia summary statistics identified 203 associated genes, meeting the Bonferroni-derived α level for 18,842 tests (*P* < 2.65 × 10^−6^). Most of these genes associated with dyslexia (*N* = 150/203) were also present in associated regions detected by GWAS, while 53 fell outside of associated regions from the SNP-based screen (Table S5). The overall number of associated genes (*N* = 203) was higher than the 172 detected in the gene-based testing of the original dyslexia study [11] (Table S5). One-hundred and twenty-six of the 172 genes associated in Doust et al. fall within regions that met genome-wide significance in the present dyslexia multivariate GWAS.

Eising et al. (2022) did not report a gene-based association analysis, however, our own analysis using their data showed one gene meeting the significance threshold (*AC079354.1*, *P* = 7.4 × 10^−7^), with candidate genes *DOCK7* and *USP1* showing the next strongest associations [9].

MAGMA gene-set analysis detected enrichment of four biological pathways from 9113 curated gene sets and gene ontology (GO) terms (Table S6) (Bonferroni threshold of *P* ≤ 5.49 × 10^−6^). The Reactome term for oncogene induced senescence (Beta = 0.82, SE = 0.18, *P* = 1.78 × 10^−6^), Verhaak glioblastoma proneural (genes correlated with proneural type of glioblastoma multiforme tumors) (Beta = 0.36, SE = 0.03, *P* = 2.88 × 10^−6^), GO-biological process of cell part morphogenesis (Beta = 0.19, SE = 0.03, *P* = 3.34 × 10^−6^) and GO-cellular compartment synapse (Beta = 0.13, SE = 0.03, *P* = 4.54 × 10^−6^) showed significant associations. Other sub-threshold pathways were GO-molecular function proton channel activity (*P* = 3.12 × 10^−5^), adherens junction interactions (Reactome) (*P* = 3.66 × 10^−5^) and post-synapse (cellular compartment) (*P* = 4.87 × 10^−5^).

### Variant mapping and functional annotation

VEP annotation of candidate SNPs (*N* = 14,663) found in LD (*R*^2^ ≥ 0.6) with one of the independent significant SNPs, including tagged SNPs from the 1000 Genomes reference panel, resulted in 138,467 individual candidate SNP annotations, due to multiple transcript variants and multiallelic sites. Intronic variants were most common (58%) with coding variants making up 0.29% (*N* = 397) (Figure S40a). Of the 397 coding variants, 53% were missense and 6% were stop-gains (Figure S40b).

Nine variants predicted as damaging by SIFT and PolyPhen and with CADD scores ≥ 20, an indication for possible deleterious effects of the variants, were found in six genes: rs11142 (chr1:109897103, tag SNP) allelic variants C and T in *SORT1*; rs1983864 (chr10:100017453, tag SNP) in *LOXL4*; rs10891314 (chr11:111916647) in *DLAT* with allelic variants A and T; rs8539 (chr 2:198362018, tag SNP) in *HSPD1*; rs1064213 (chr2:198950240, tag SNP) in *PLCL1*; rs1130146 (chr 20:47859217, tag SNP) in *DDX27*; and rs11539148 (chr3:49138810, tag SNP) in *QARS* (Table S7). Five of the variants with CADD scores ≥ 20 (but not annotated by SIFT or PolyPhen) were predicted to result in stop-gain changes: rs2424922 alleles T and A (chr20:31386449, tag SNP) (both alleles annotated at stop-gained and splice region variants), and rs6058891 (chr 20:31386347, tag SNP) in *DNMT3B*; rs6169 (chr11:30255185, tag SNP) in *FSHB*; and rs3764090 (chr13:50008301, tag SNP) in *AL136218.1*.

At the gene level, 1115 genes were contained within genome-wide significant regions (Table S8). Sensitivity to loss-of-function was annotated with probability of loss-intolerance scores (pLI) and sensitivity to non-coding variation in regulatory sequences was annotated with non-coding residual intolerance scores (ncRVIS). Two-hundred and twenty-four genes (20.1%) were predicted as loss-of-function intolerant by pLI ≥ 0.9, and seventeen (1.5%) were predicted as less tolerant to non-coding variation by ncRVIS ≥ 2.0. Four genes (*SIK2*, *PTPN14, ARNT2* and *XYLT1*) were predicted as intolerant by both metrics (pLI ≥ 0.9 and ncRVIS ≥ 2.0).

Two-hundred and nineteen genes (*N* = 219/1115, 19.6%) located within associated regions showed evidence of association with expression QTLs (eQTL) in brain tissue (minimum eQTL false discovery rate of mapped SNPs (*P* ≤ 0.05)) (Table S8). The strongest eQTL associations were for *DHRS11* (*P* = 6.36 × 10^−77^), *CYB561* (*P* = 1.41 × 10^−69^), *INA* (*P* = 3.68 × 10^−60^), *ZNF660* (*P* = 1.59 × 10^−57^) and *CCDC171* (*P* = 6.91 × 10^−56^).

### Functional enrichment using partitioned heritability and gene property analysis

To examine the tissue-specific expression profiles of genes implicated in dyslexia, we used MAGMA gene property analysis within FUMA. Using RNA-seq data from the Genotype-Tissue Expression (GTEx) project, we found significant enrichments of genes associated with dyslexia in brain tissue: 11 brain regions tested showed significantly higher expression levels of dyslexia associated genes (*P* ≤ 4.03 × 10^−4^), particularly the cerebellum, cerebellar hemisphere and frontal cortex (Figure S41, Table S9). As MAGMA analysis corrects for the average expression level in the dataset, a significant association indicates that genes associated with dyslexia have a higher expression in that tissue relative to the average expression within the dataset.

**Fig. 3:**
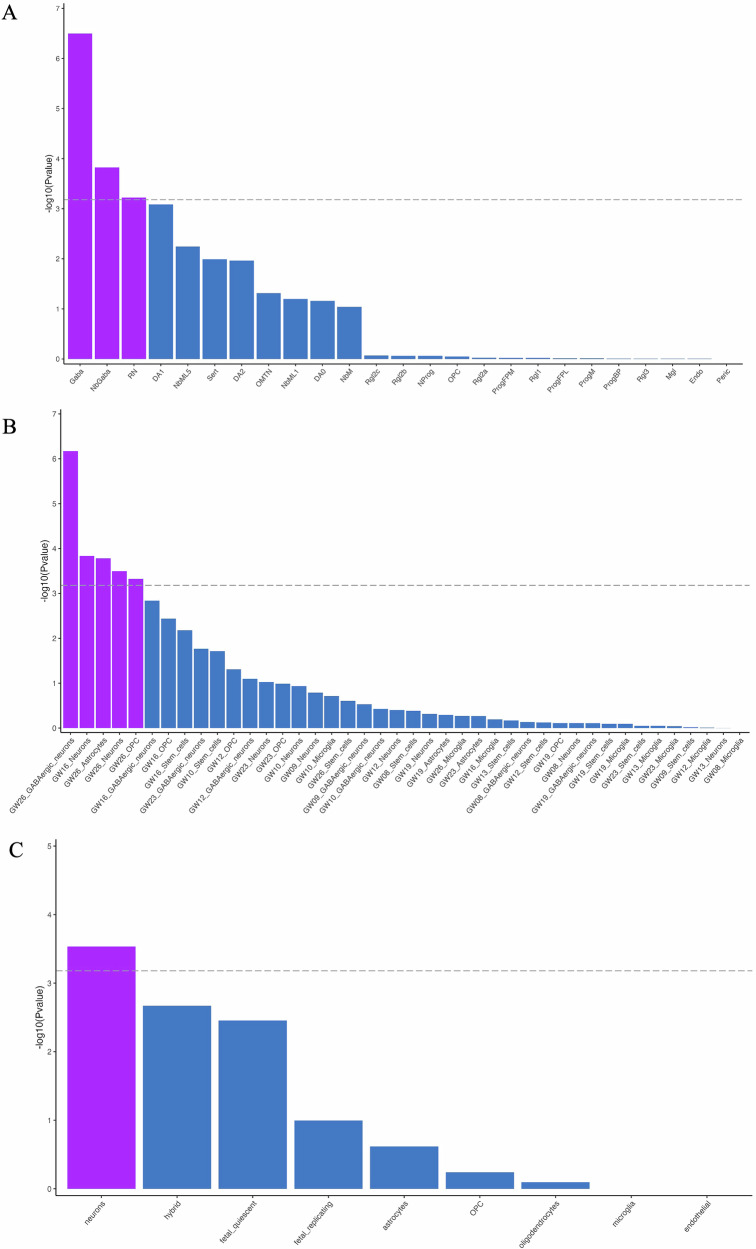
Analysis of expression patterns of dyslexia-associated genes in the developing human brain. MAGMA gene property analyses of dyslexia associated genes with single cell gene expression data from **A** embryonic ventral midbrain from 6–11 post conception weeks (pcw), **B** embryonic prefrontal cortex from 8–26 post conception/ gestational weeks (GW), **C** human fetal and adult cortex. DA0-1 dopaminergic neurons, Endo endothelial cells, Gaba GABAergic neurons, Mgl microglia, NbGaba neuroblast GABAergic, NbM medial neuroblast, NbML1-5 mediolateral neuroblasts, NProg neuronal progenitor, OMTN oculomotor and trochlear nucleus, OPC oligodendrocyte precursor cells, Peric pericytes, ProgBP progenitor basal plate, ProgFPL progenitor medial floorplate, ProgM progenitor midline, Rgl1-3 radial glia-like cells, RN red nucleus, Sert serotonergic.

Next, we tested for enrichment within the BrainSpan data set, consisting of RNA-seq from 11 developmental stages (Figure S42, Table S10) and 29 ages (Figure S43, Table S11) of human brains. No associations met Bonferroni correction for 124 tests (*P* ≤ 4.03 × 10^−4^). To further investigate enrichment of gene expression within the developing human brain, we tested for associations with specific cell types in single cell RNA-seq (scRNA) data using MAGMA within FUMA. Embryonic ventral midbrain (6–11 post conception weeks (pcw)) revealed three enriched cell types meeting Bonferroni correction (*P* < 6.58 × 10^−4^, 76 tests) (Fig. 3a, Table S12): GABAergic neurons (Gaba, *P* = 3.19 × 10^−7^), neuroblast GABAergic neurons (NbGaba, *P* = 1.49 × 10^−4^), and red nucleus neurons (RN, *P* = 5.99 × 10^−4^). Embryonic prefrontal cortex scRNA-seq data spanning 8–26 pcw, showed significant enrichment in neurons at 16 pcw (*P* = 1.46 × 10^−4^), and then GABAergic neurons (*P* = 6.76 × 10^−7^), astrocytes (*P* = 1.65 × 10^−4^), neurons (*P* = 3.18 × 10^−4^) and oligodendrocyte precursor cells (OPC) (*P* = 2.85 × 10^−4^) at 26 pcw (Fig. 3b, Table S13). Finally, scRNA-seq data from fetal and adult human cortex showed significant expression in adult cortical neurons (*P* = 2.94 × 10^−4^) (Fig. 3c, Table S14).

**Fig. 4:**
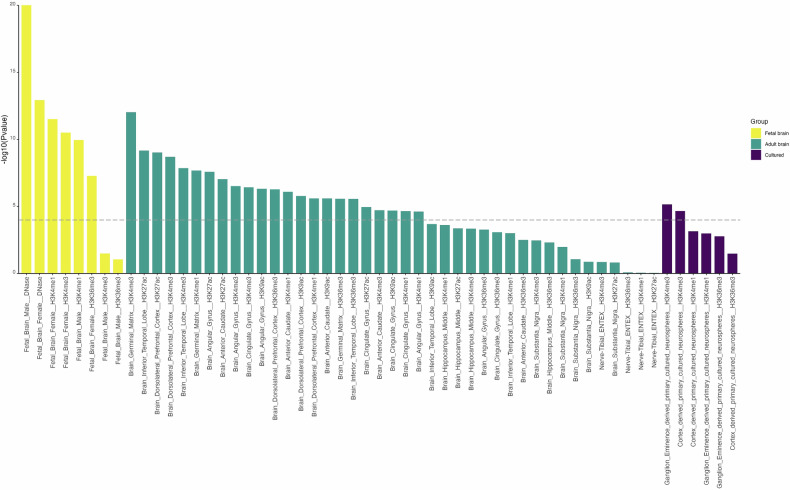
Partitioned heritability enrichment analysis of chromatin signatures. SNP-based heritability of the multivariate dyslexia GWAS is significantly enriched in brain enhancers, promoters and transcribed regions. 489 annotation of tissue-specific chromatin signatures were used to analyze the GWAS results with LDSC heritability partitioning. Only brain-related annotations are shown. *P* values are plotted on the y axis as -log_10_.

Heritability partitioning by LDSC identified statistically significant enrichment of variants associated with dyslexia in chromatin signatures annotated in fetal and adult brain tissues obtained from the Roadmap Epigenomics project and Enhancing GTEx project (ENTEx) (Fig. 4, Table S15). Out of 489 chromatin signatures tested, thirty-one annotations were enriched (Bonferroni corrected threshold *P* < 1.02 × 10^−4^), from fetal brain (*N* = 6), adult brain (*N* = 23) and primary neuronal cultured cell lines (*N* = 2), across a range of chromatin signatures of (active) enhancers and promoters and actively transcribed regions.

### Polygenic index prediction in NCDS

The multivariate dyslexia polygenic index (PGI) was computed in an independent cohort across five developmental stages and a composite reading score combining all ages. When calculated using PRSice2, the dyslexia PGI explained between 1.57 and 3.61% of variance in reading ability in the NCDS cohort. Explained variance for measures of reading proficiency was 2.22% at age 7 years (*N* = 5712), 2.24% at age 11 years (*N* = 5528), 1.57% at age 16 years (*N* = 4809) and 2.40% for overall composite measure of reading proficiency across all ages (*N* = 3089). Binary measures of self-reported difficulties with reading explained the highest proportion of variance, at 3.2% (OR = 1.73, 95% CI = 1.48–2.02) at age 23 years (N_cases_ = 167, N_controls_ = 5 288) and 2% (OR = 1.54, 95% CIs = 1.33–1.78) at age 33 years (N_cases_ = 203, N_controls_ = 5497) (Figures S44–S49; Table S16). SBayesRC yielded improved predictions: estimates of variance explained by PGI ranged between 2.3 and 4.7%. The PGI for dyslexia predicted 3.6% at age 7 years, 3.5% at age 11 years, 2.3% at age 16 years and 4.1% in the reading composite across all ages. Similarly, there was increased predictive power for self-reported reading difficulties at ages 23 years (4.7%, OR = 1.98, CI = 1.69–2.33) and 33 years (3.3%, OR = 1.74, CI = 1.51–2.01) in the NCDS cohort (Table S16).

### Polygenic selection analysis

**Fig. 5:**
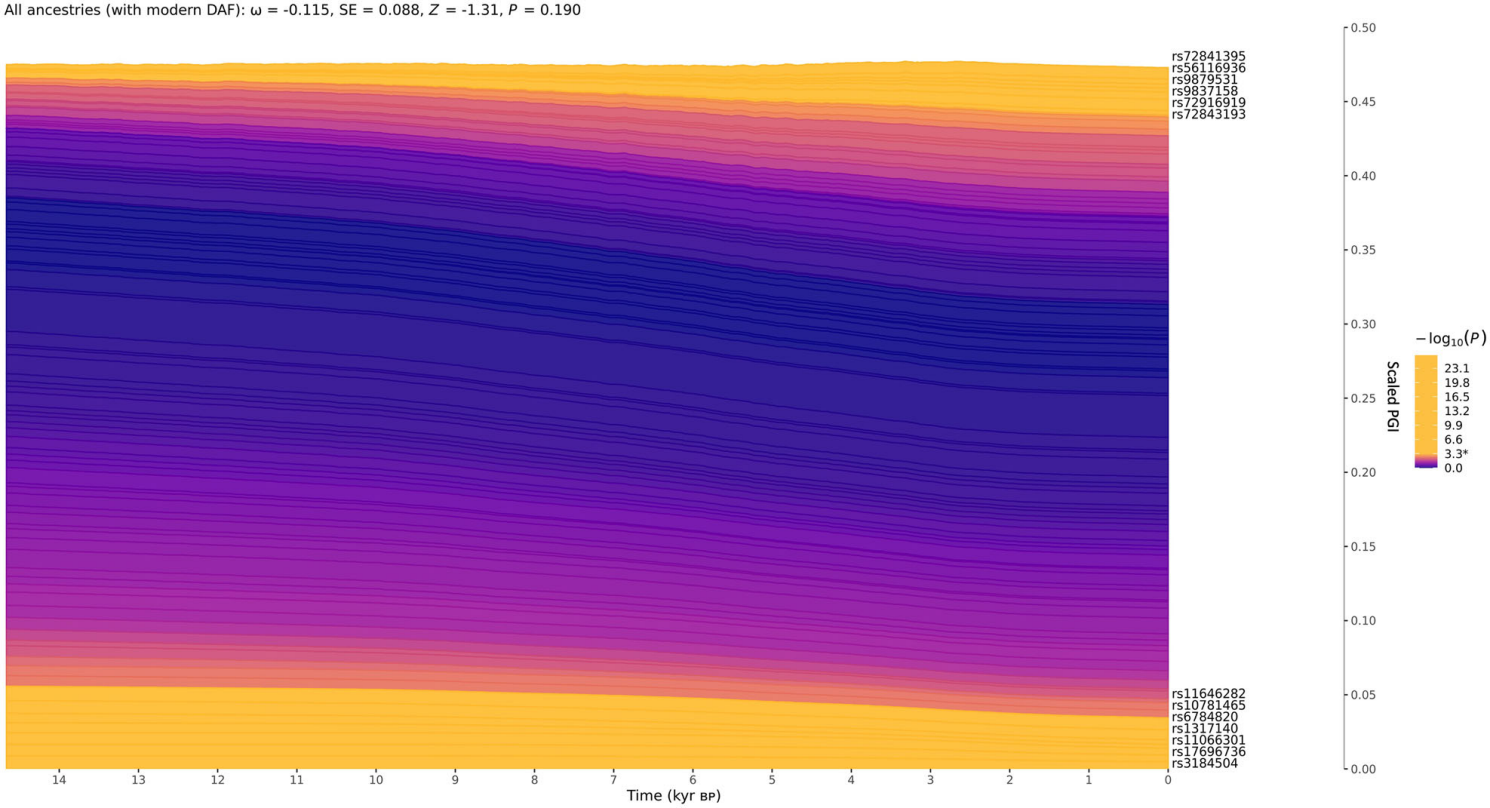
No evidence for directional selection of dyslexia associated SNPs. Stacked line plot of the ancient ancestry PALM analysis, showing the contribution of SNPs to dyslexia over time. SNPs are shown as stacked lines, the width of each line being proportional to the population frequency of the positive effect allele, weighted by its effect size. When a line widens over time the positive effect allele has increased in frequency, and vice versa. SNPs are sorted by the magnitude and direction of selection, with positively selected SNPs at the top, negatively selected SNPs at the bottom, and neutral SNPs in the middle. SNPs are colored by their corresponding *P*-value in a single locus selection test. The asterisk on the scale bar marks the Bonferroni corrected significance threshold, and nominally significant SNPs are shown in yellow and labelled by their rsIDs. The Y-axis shows the scaled average polygenic index (PGI) in the population, ranging from 0 to 1, with 1 corresponding to the maximum possible average PGI (i.e. when all individuals in the population are homozygous for all positive effect alleles) and the X-axis shows time in units of thousands of years before present (kyr BP).

The polygenic selection analysis examined if there was evidence of selection on alleles associated with dyslexia seen through the past 15,000 years of human history. Essentially, this looks for differences in allele frequencies in variants associated with a trait, between the ancient ancestral population(s) and the present day. Such shifts in frequency indicate that selection acted to change the allele frequency of that variant in response to environmental pressures. From the 211 significant independent variants within 80 loci associated with dyslexia in the multivariate GWAS, 104 were retained after clumping and present at high quality in the previously generated imputed ancestral data set [29, 30, 33]. Overall analyses of these SNPs identified no evidence of directional selection acting on dyslexia over the past 15,000 years (ω = −0.115, SE = 0.088, Z = 01.31, *P* = 0.19) (Fig. 5). Thirteen SNPs showed individually statistically significant evidence of directional selection, after accounting for the number of tests (*P* ≤ 4.8 × 10^−4^) (Table S17). It is plausible that these variants individually showed modest directional selection because of their individual contributions to other traits, given that our overall analysis showed no evidence of selection acting on dyslexia through the past 15,000 years (Supplementary Note).

## Discussion

We performed a multivariate GWAS using MTAG on dyslexia using summary statistics from the two largest reading-related studies to increase the effective sample size to 1,228,832 and identified 80 independent loci associated with dyslexia. The most significantly associated independent SNPs; rs13082684 (*PPP2R3A*), rs2426117 (*CSE1L*) and rs9696811 (located between *PTPA* and *IER5L*), were consistent with loci reported in the univariate dyslexia GWAS [11]. We identified thirty-six regions not previously associated with dyslexia at genome-wide significance threshold [11, 34]. Taking a conservative approach of novel regions not meeting significance threshold in either study or the univariate dyslexia uncorrected summary statistics, we identified thirteen novel loci associated with dyslexia.

Three novel lead SNPs showed prior associations with brain-related phenotypes. Novel lead SNP, rs362307 in *HTT*, has been associated with a range of cognitive traits with potential relevance to reading ability in prior studies, including cognitive function [37] and educational attainment [36]. GenomicSEM and genetic correlation analyses have shown that reading-related traits have substantial (albeit partial) genetic overlaps with educational attainment and IQ [9, 11], and therefore SNPs involved in general cognition are likely to be important across traits. Interestingly, rs362307 is used as a marker for identification of the A1 haplotype of the *HTT* gene in Europeans. The A1 haplotype contains a gain-of-function mutation known to cause Huntington disease [42]. Two novel regions, containing lead SNPs rs12653108 (*IPO11, HTR1A*) and rs79445414 (*DUSP26, UNC5D*), showed previous significant associations with ADHD [40, 41]. Preliminary evidence for six of these novel regions came from a recent study using GenomicSEM, where they were short of the significance threshold for pleiotropic loci for dyslexia and ADHD [34]. The presence of these pleiotropic regions and high cooccurrence between dyslexia and ADHD (25–40%) [43, 44] reflects the increasing importance of shared genetics between the traits.

The most significantly associated SNP identified by the GenLang word-reading meta-GWAS [9], rs11208009, did not reach genome-wide significance in the present multivariate study (*P* = 7.85 × 10^−5^). It fell within a region that reached suggestive significance (chr1:62900811–63199936, *P* = 1.9 × 10^−6^) and fully overlapped with the previously reported region. Interestingly, rs11208009 has been associated with phonological awareness, but not with other processes related to dyslexia such as rapid automized naming [45]. Phonological awareness could be better captured by quantitative measures of word reading than by self-reported dyslexia diagnosis which may include broader processing difficulties.

SNP-based heritability estimates using SumHer [22], which utilizes both linkage disequilibrium and allele frequencies, increased the liability-scale ${h}_{{\rm{snp}}}^{2}$ estimates for the multivariate dyslexia GWAS to 16.4% at 5% dyslexia population prevalence, 19.2% at 7% prevalence, and 20.4% at 10% prevalence. These improved on our LDSC estimates for multivariate dyslexia of 12.9% (5% dyslexia population prevalence), 14.3% (7% prevalence) and 16% (10% prevalence).

LDSC ${h}_{{\rm{snp}}}^{2}$ estimates for the univariate dyslexia study were 15.2% (${h}_{{\rm{snp}}}^{2}=0.152$, SE = 0.006, 95% CI 0.14–0.164) using the 23andMe sample prevalence of 5, and 18.9% (${h}_{{\rm{snp}}}^{2}=0.189$, SE = 0.008, 95% CI 0.173–0.205) using a 10% population prevalence of dyslexia, thought to be representative of the general population [11]. The lower ${h}_{{\rm{snp}}}^{2}$ LDSC estimates in the present study may reflect the phenotype differences between univariate studies: self-reported dyslexia in adults unselected for cooccurring conditions such as ADHD versus quantitative measures of reading in children and young people. It may also be influenced by challenges in estimating both the sample and population prevalence of dyslexia: 5% for the 23andMe cohort [11] and difficult to estimate in the GenLang cohort as this includes cohorts of children who are yet to attain maximal reading [9], while the population prevalence of dyslexia varies widely (5–17.5%) [46–48].

We predicted a maximum of 4.7% (OR = 1.97, CI = 1.69–2.33) of trait variance in reading measures in the NCDS cohort using the dyslexia polygenic index derived from our multivariate GWAS. This is an improvement on the previous predictions using the univariate dyslexia GWAS on measures of reading in similar population-based cohorts, where 2.9% of variance was explained in adolescents and ~2% in adults [11]. Predictions using SBayesRC across longitudinal measures at five ages within the NCDS ranged from 2.3–4.7% suggesting that this part of the genetic contribution of reading is stable through schooling and into adulthood where the measures are more focused on reading difficulties and thus, more aligned with dyslexia. Other studies have used phenotypically related PGIs to predict reading outcomes. For example, Selzam et al. (2017) [49] used a years-in-education PGI [50] to predict 5% of reading variance at 14 years of age. Future studies might test whether our dyslexia PGI explains incremental variance in reading variation above an educational attainment PGI, which is a broader phenotype encompassing cognitive and non-cognitive factors.

Genetic correlations between the multivariate dyslexia GWAS and other phenotypes showed a high degree of similarity to the profiles of genetic correlations seen for both the univariate GWAS studies of dyslexia and word reading. Key findings were consistent with the univariate GWAS, that is, positive genetic correlations with measures of lower socio-economic status and less desirable workplace conditions, and negative correlations with higher socioeconomic and health measures. Notably, the strongest correlation for the multivariate dyslexia GWAS was verbal-numerical reasoning sub-question F17- synonyms in the UK Biobank (−0.62, SE = 0.06) followed by the full verbal-numerical reasoning measure (−0.56, SE = 0.03). Verbal-numerical reasoning was the among the strongest correlations for the univariate dyslexia at −0.5 (SE = 0.03) [11].

Correlations with other neurodevelopmental traits were consistent between the univariate and multivariate GWASs. The strongest correlation of the univariate dyslexia GWAS was with ADHD at 0.53 (SE = 0.01) [11], and although this correlation was lower in absolute magnitude for the multivariate dyslexia analysis (0.4, SE = 0.03), it remains consistent with the literature linking ADHD with reading and language outcomes [5, 51]. The observation that genetic correlation with ADHD is stronger with the univariate dyslexia (0.53) than the multivariate dyslexia (0.4), but the same as the univariate word-reading (−0.4) may be due to the presence of participants self-reporting ADHD diagnosis in the 23andMe cohort and thus inflating the correlation. Consistent with previous findings [9, 11], we found no significant genetic correlation between autism spectrum disorder (ASD) and dyslexia.

We found strong correlations between dyslexia and several measures of chronic pain including all over body pain (0.5, SE = 0.06), neck and should pain (0.47, SE = 0.1) and leg pain on walking (0.44, SE = 0.04) (UK Biobank). The prevalence of chronic pain is higher in both neurodevelopmental [52] and psychiatric conditions [53]. The underlying mechanism remains unelucidated, however, the genetic overlap between pain-related phenotypes and neurodevelopmental traits may hint at a shared biological basis [11, 54].

Gene-set analysis revealed four enriched biological pathways implicated in dyslexia: the Reactome term for oncogene induced senescence, genes correlated with proneural type of glioblastoma multiforme tumors (Verhaak glioblastoma proneural), the gene-ontology biological process of cell part morphogenesis, and the term for cellular compartment neural synapses, hinting at essential neuronal mechanisms.

Our analysis of expression patterns of dyslexia-associated genes in the developing human brain offered further evidence for a role in early developmental processes, implicating GABAergic and red neurons in embryonic midbrain, GABAergic neurons, astrocytes and oligodendrocyte precursor cells in embryonic prefrontal cortex, as well as cortical neurons in adults. These findings echo the cell type analysis reported in the GenLang GWAS study, where an enrichment in red nucleus neurons and a trend towards enrichment in fetal GABAergic neurons was observed [9]. Price and colleagues reported evidence supporting neuronal migration/axon guidance as potential pathways using a candidate gene-set approach for known neurodevelopmental genes in a hypothesis-driven association analysis of word reading which included the GenLang meta-GWAS [55]. More recently, the same research group implicated glutamatergic (excitatory) and GABAergic (inhibitory) neurons in the adult cortex in word reading, using a subset of the GenLang cohorts (*N* = 5054) [56]. The sample used in the present study overlaps with that of the prior work [55, 56], which may contribute to the consistency between the two sets of results. This work offers support for the GABAergic inhibitory system as a future focus for connecting genetics to neuronal mechanisms.

Polygenic selection analysis found no significant selection observed from ancestral populations suggesting that the genetic influences on dyslexia were not specifically selected for or against in the transition between hunter gatherer and farmers in Europeans. This finding may be considered unsurprising since reading is many thousands of years old but has only recently become widespread and has no obvious selection pressure on reproductive fitness. Because reading processes are highly dependent on brain circuits that evolved in support of spoken language, it was still possible that we might have detected signals with relevance to aspects of language evolution. The consistency of the PGI through the past 15 k years of history in northern Europe suggests it has not been affected by any major social or societal changes that have taken place in history such as the transition to farming, although it is important to note that our PGI accounts for only a modest proportion of heritable dyslexia. We identified thirteen SNPs that showed individually significant changes through recent history, although the directions of effect on dyslexia were mixed. We speculate that the patterns observed for these thirteen SNPs are most likely due to selection pressures acting on pleiotropic traits. Analyses that examined deeper timescales in our evolutionary history (30 million years ago to 50,000 years ago) were performed in both the Eising et al. [9] and Doust et al. [11] papers. Adopting approaches developed for studying human brain structure evolution [57], five annotations reflecting complementary aspects of human evolution were examined. Doust et al. found no evidence of enrichment for the tested annotations [11]. Eising et al. found evidence of an enrichment in archaic deserts; long regions in the human genome where there is an absence of Neanderthal admixture, suggesting these regions may be intolerant to gene flow and therefore harboring variants essential to *Homo sapiens* [9]. The findings suggested that these archaic desert regions could contain genetic variations that contribute more to reading and language traits in modern humans than expected by chance.

Through implementation of multivariate GWAS analysis combining work on quantitative measures with self-reported diagnosis data, we have produced the largest genetic study of dyslexia (effective *N* = 1,228,832) to date. Our findings account for up to 16.4% $({h}_{{snp}}^{2})$ at 5% prevalence, and 20.4% at 10% population prevalence of dyslexia. We identified thirteen novel loci associated with dyslexia and implicated early brain developmental processes in the biological underpinnings of reading.

## Supplementary information

Supplementary tables

Supplementary information

## Data Availability

The univariate GWAS summary statistics for word reading are available to download from the GenLang website https://www.genlang.org/downloads.html. The full GWAS summary statistics for the 23andMe discovery data set are made available through 23andMe to qualified researchers under an agreement with 23andMe that protects the privacy of the 23andMe participants. Please visit https://research.23andme.com/collaborate/#dataset-access/ for more information and to apply to access the data. The multivariate summary statistics for dyslexia and reading ability generated by this study are available through 23andMe to qualified researchers, as described. The Github repository for this research is available at https://github.com/hayley-mountford/multivariate_GWAS_dyslexia.
