# Supplementary material for: Multivariate genome-wide association analysis of dyslexia and quantitative reading skill improves gene discovery
Source: Transl Psychiatry. 2025 Aug 18;15:289. doi: 10.1038/s41398-025-03514-0 (PMC12361447; doi:10.1038/s41398-025-03514-0)
Supplement: Supplementary file 2 — Supplementary information [file 41398_2025_3514_MOESM2_ESM.docx]

**Supplementary Methods**

**Summary Statistics**

GWAS summary statistics for quantitative measures of word reading were available in the GenLang meta-analysis of 27,180 (male = 13,874, female = 13,202, no information = 104) participants of European ancestry only across 18 studies ^1^. Participants were children or young adults ranging from 5-26 years ^1^. Measures depended on the contributing cohort and are detailed in Eising *et al* ^1^, with the Test of Word Reading Efficiency (TOWRE) and Wide Range Achievement Test (WRAT) most common. Summary statistics without genomic-control correction were used.

Summary statistics for self-reported dyslexia diagnosis (23andMe, Inc.) were reported in Doust *et al* (2022) ^2^. Participants responded “Yes” to “have you been diagnosed with dyslexia” (N_cases_ = 51,800, female = 30,287, male = 21,513), and N_controls_ = 1,087,070 responded “No” (female = 641,016, male = 446,054). All participants were over 18 years of age (mean 49.6 (cases) and 51.7 years (controls)). Participants with non-European ancestry were excluded prior to analysis. Summary statistics without genomic-control correction were used ^2^.

23andMe participants provided informed consent and volunteered to participate in the research online, under a protocol approved by the external AAHRPP-accredited Salus IRB (https://www.versiticlinicaltrials.org/salusirb).

Both sets of summary statistics were annotated with rsIDs (build hg19), formatted for MTAG, and Z scores were calculated in R. Variants with imputation quality <0.8 or minor allele frequency of <0.01 were excluded prior to analysis.

**Multivariate GWAS using MTAG**

Multivariate GWAS was performed with MTAG ^3^ using default settings and false discovery rate (FDR) calculation. We note that minimal sample overlap is expected, given that the GWAS of dyslexia includes a single sample of adults predominantly US-based 23andMe, Inc. research participants and the GWAS of word reading is a meta-analysis of cohorts of children and young adults with a minority from the US. Moreover, MTAG uses bivariate LD score regression to control for sample overlap between cohorts.

Associations were visualised using ggplot2 ^4^. Individual regions were visualised using LocusZoom (http://locuszoom.org). FUMA v1.5.0 was used to annotate the associated regions that met the genome-wide significance threshold ^5^ (P ≤5x10^-8^, *R^2^* <0.6, and <250kb maximum distance between LD blocks to merge into one genomic locus).

**Heritability and Genetic Correlation**

SNP-based heritability (*h*^2^_snp_) was estimated using LDSC v1.0.1 ^6^. European LD reference panel was obtained from <https://alkesgroup.broadinstitute.org/LDSCORE>. We also applied a second method for estimating *h*^2^_snp_ - SumHer within LDAK (<https://dougspeed.com/>) ^7^, using the BLD-LDAK model HapMap3 GBR pre-computed based on 2000 white British UKBiobank individuals. Sample prevalence was 5% in both analyses and based on the 23andMe cohort prevalence of cases ^2^. The effective sample size (N = 1,228,832) for the multivariate dyslexia GWAS output by MTAG was applied.

GWAS summary statistics were obtained from the Complex-Traits Virtual Genetics Lab (CTG-VL) platform (https://vl.genoma.io), except for ASD ^8^ and ADHD ^9^ which were downloaded from the Psychiatric Genomics Consortium repository (https://www.med.unc.edu/pgc/download-results). Genetic correlations were performed using LD-Score v1.0.1 within the CTG-VL platform and considered significant at a Bonferroni corrected threshold of P ≤ 1.77x10^-5^ from 2824 tests.

Due to the difference in sample size between the dyslexia and GenLang univariate summary statistics, we cross-checked genetic correlations with LDSC, between each set of summary statistics and the reading ability MTAG output. We used *r_2_* and false discovery rate (FDR) to assess potential effects from the sample size and power imbalance.

**Gene-based and Gene-set Analysis**

Gene-based associations were calculated using MAGMA v1.08 ^10^ using SNP2GENE within the FUMA interface (https://fuma.ctglab.nl/) for 18,842 genes and were considered significant at a Bonferroni corrected threshold of P ≤ 2.65x10^-6^. Gene-set analyses of biological pathways defined by gene ontology (GO) pathways and curated gene-sets were examined using MAGMA. Gene-sets containing fewer than 20 genes were excluded from analysis, therefore 9,113 biological pathways were tested and a Bonferroni corrected threshold of P ≤ 5.49x10^-6^ was applied.

**Functional Mapping, Annotation and Partitioned Heritability**

Fine mapping and annotations were performed using the Variant Effect Predictor (VEP) online tool (http://grch37.ensembl.org/) on the list of candidate SNPs present with R^2^ ≤0.6 with an independent significant SNP generated by FUMA, and including tagged SNPs extracted from the 1000 genomes reference panel. Variants were considered potentially damaging if they were annotated as probably damaging by PolyPhen2 and deleterious by SIFT. Expression QTL analysis was performed using PsychENCODE eQTLs, BRAINEAC, eQTLcatalogue BrainSeq, and GTEx v8 Brain databases within FUMA, and the false discovery rate threshold (eqtlMapminQ) of ≤0.05 applied.

MAGMA, within FUMA, was used to test for enrichment of tissue-specific annotations. For this, we used bulk RNA-seq expression profiles from 53 tissue types from GTEx v8, and BrainSpan RNA-sequencing from 29 ages spanning 11 developmental stages.

To interrogate cell- and region-specific resolution, we accessed single-cell RNA-seq (scRNA) data via the Cell Type function within FUMA. Expression data from human embryonic ventral mid-brain (6-11 weeks post gestation) (GSE76381), human embryonic prefrontal cortex (8-26 weeks post gestation) (GSE104276), and human adult and fetal cortex (GSE67835) datasets were tested.

We partitioned SNP heritability using stratified LDSC, as described by Finucane *et al* ^11^, to determine if significantly more SNPs clustered within tissue-specific chromatin modification patterns than expected by chance, based on the proportion of SNPs that map within these types of genomic regions. Annotations were based on data from the Roadmap Epigenomics project and Enhancing GTEx project (ENTEx). LD scores, regression weights and European allele frequencies were obtained from https://alkesgroup.broadinstitute.org/LDSCORE.

**Polygenic Index Analysis**

Polygenic index (PGI) for dyslexia was calculated for the National Child Development Study (NCDS); a large UK birth cohort study born in 1958 ^12^, with extensive genetic data and longitudinal reading measures as described in Bridges *et al* ^13^. Individuals genotyped on one of the two lowest resolution arrays (Illumina 15k Custom chip and Affymetrix 500k) were excluded prior to analysis due to lack of available data from chromosome X, resulting in a cohort of N = 6,410 with imputed genotypes. Six measures of functional reading were used, consisting of composite measures at ages 7 years, 11 years, 16 years and across all time points, and binary measures of difficulties at ages 23 years and 33 years ^13^.

The dyslexia PGI was estimated for the NCDS cohort using PRSice2 v2.3.5 ^14^. Suitable SNP GWAS P value thresholds were generated by PRSice2 and plotted using ggplot (supplementary figures 8-12). PGIs were also estimated using SBayesRC, a recently developed method which uses functional information in the SNP weighting algorithm ^15^.

PGIs were standardised to a mean of zero and standard deviation of one. Linear or logistic regression were performed using sex, genotyping array and 10 genetic principal components as covariates ^13^ in either a null (phenotype ~ covariates) or full model (phenotype ~ PGI + covariates). Odds ratio and confidence intervals for age 23 and age 33 variables, as well as PGI analyses, were calculated in R.

**Polygenic Selection Analysis**

We sought to identify evidence of polygenic selection for dyslexia using a large panel of 1015 imputed ancient genomes, sampled from across West Eurasia^16, 17^. This dataset represents a dense transect of ancient individuals sampled over the last 15k years with local ancestry contributing to present day Europeans. We ascertained statistically independent SNPs associated with dyslexia by filtering our genome-wide summary statistics to only retain positions imputed with high confidence in the ancient dataset. LD-clumping was performed using Plink 1.90b4 with a window size of 250kb, maximum *R^2^* threshold of 0.05, and maximum P ≤ 5x10^-8^ using the 1000 Genomes Project phase 3 European populations (GBR, FIN, TSI) as the reference panel. We then inferred allele frequency trajectories and selection coefficients using CLUES^18^ and exported the posterior likelihood densities. Finally, we modelled the polygenic selection gradient for reading ability with PALM^19^ using imputed ancestral data generated and method described by Barrie *et al* ^20^.

**Supplementary Notes**

**MTAG Quality Control**

The multivariate GWAS for dyslexia using MTAG ^3^ resulted in a GWAS equivalent maximum sample size of N = 1,228,832 (MTAG χ^2^ = 1.69) with a maximum false discovery rate of 0.00024. Analysis with LDSC found moderate genomic inflation (λ = 1.573, χ^2^ = 1.773, LD intercept = 1.017 (0.012)) which is consistent with a highly polygenic trait in a well powered GWAS. Additional genomic control correction was not deemed necessary as the meta-analysis is based on the genetic correlation derived using LDSC, therefore MTAG corrects for population stratification.

The multivariate GWAS for reading ability resulted in a GWAS equivalent sample size of N = 102,082 (MTAG χ^2^ = 1.093) and a maximum false discovery rate of 0.06. The FDR indicates that 6% of SNPs are likely false positives, meaning they are null for the trait of interest (reading ability), but not null for univariate dyslexia. Further, analysis with LDSC identified moderate genomic inflation (λ = 1.28, χ^2^ = 1.431, LD intercept = 0.843 (0.01)).

As the two constituent univariate summary statistics were derived from GWAS efforts with different sample sizes (N = 27,180 versus N_cases_ = 51,800/ N_controls_ = 1,087,070) and type of measure (quantitatively assessed continuous measure versus self-reported binary), we investigated whether the multivariate GWAS for reading ability was overwhelmed by signal from the dyslexia study. To do this we estimated genetic correlations with LDSC between multivariate reading ability and univariate dyslexia (*r*_g_ = -0.98, SE = 0.00, Z = -334.76, P = 0.00), and then between multivariate reading ability and univariate GenLang word reading (*r*_g_ = 0.84, SE = 0.03, Z = 27.63, P = 4.66 x 10^-168^). While univariate word reading showed a substantial genetic correlation with multivariate reading ability (*r*_g_ = 0.84) showing evidence that it is indeed capturing genetic variation associated with quantitative reading ability, the near-complete correlation with the univariate dyslexia GWAS (*r*_g_ = -0.98) suggested it was. Taken together, it is likely that the multivariate reading ability GWAS was saturated by the univariate dyslexia GWAS. We therefore focussed downstream analyses only on the multivariate dyslexia GWAS.

**Polygenic Selection Analysis**

One-hundred and four statistically independent SNPs present in the ancient dataset were retained after LD-clumping (R2 <0.05, window 250kb). Overall, the independent SNPs associated with dyslexia did not show evidence of selection over the past 15k years (ω = -0.115, SE = 0.088, Z = 01.31, P = 0.19). Thirteen individual SNPs showed statistically significant evidence of directional selection (Bonferroni threshold of P ≤ 4.8x10^-4^ for 104 tests), however it is likely that these variants show modest directional selection because of their contributions to traits other than dyslexia.

Independent SNP rs3184504 (P = 8.14x10^-27^) mapped to genes *ATXN2* and *SH2B3,* showed the most pronounced increase in allele frequency over time and a protective effect on dyslexia. The variant is also associated with a wide range of immune, metabolic and disease traits including platelet count ^21^, type 1 diabetes ^22^ and hypothyroidism ^23^. Interestingly, the next two strongest signals are SNPs in nearby regions on chromosome 12, also protective and increasing in frequency over time; rs17696736 (P = 4.94x10^-26^) in the gene *NAA35*, has previous associations including blood pressure ^24^ and kidney function ^25^; and rs11066301 (P = 7.16x10^-25^) in *PTPN11* is associated with a range of metabolic function and disease including arterial disease ^26^. All three SNPs are associated with primarily metabolic phenotypes. Finally, a fourth SNP, rs10781465 (P = 7.18x10^-9^), also showed significant rise in allele frequency and was protective for dyslexia, although no prior trait associations were reported.

Only one protective SNP (rs72843193 (P = 1.2x10^-9^) showed falling allele frequency across time, and no prior associations were reported.

The strongest signal in alleles associate with increased risk of dyslexia was in rs1317140 in the gene *TRAIP* associated with body mass index ^27^ and urate measurement ^28^, and showed decreasing allele frequency over time. Secondly, SNP rs6784820 (P = 3.73x10^-5^) in gene *TCTA*, also showing increasing risk of dyslexia, was previously associated with household income ^29^. Lastly, rs11646282 (P = 4.64x10^-4^) also showed significant falls in allele frequency over time and increasing risk for dyslexia (no associations with other traits).

The strongest signal in alleles associated with dyslexia and showing increasing allele frequency over time was rs72841395 (P = 1.79x10^-5^) (no associated traits). Finally, rs56116936 (P = 4.26x10^-5^) (no associated traits), rs9879531 (P = 7.17x10^-5^) in gene *NCK1* and associated with blood urea nitrogen measurement ^25^, rs9837158 (P = 1.12x10^-4^) (no associated traits) and rs72916919 (P = 1.22x10^-4^) (no associated traits), also showed significantly rising alleles frequencies across time and increasing risk.

**Supplementary references**

1. Eising E, Mirza-Schreiber N, de Zeeuw EL, Wang CA, Truong DT, Allegrini AG *et al.* Genome-wide analyses of individual differences in quantitatively assessed reading- and language-related skills in up to 34,000 people. *Proc Natl Acad Sci U S A* 2022; **119**(35)**:** e2202764119.

2. Doust C, Fontanillas P, Eising E, Gordon SD, Wang Z, Alagoz G *et al.* Discovery of 42 genome-wide significant loci associated with dyslexia. *Nat Genet* 2022; **54**(11)**:** 1621-1629.

3. Turley P, Walters RK, Maghzian O, Okbay A, Lee JJ, Fontana MA *et al.* Multi-trait analysis of genome-wide association summary statistics using MTAG. *Nat Genet* 2018; **50**(2)**:** 229-237.

4. Wickham H. *ggplot2 : Elegant Graphics for Data Analysis*. 2nd 2016. edn. Springer-Verlag: New York, 2016.

5. Watanabe K, Taskesen E, van Bochoven A, Posthuma D. Functional mapping and annotation of genetic associations with FUMA. *Nat Commun* 2017; **8**(1)**:** 1826.

6. Bulik-Sullivan BK, Loh PR, Finucane HK, Ripke S, Yang J, Schizophrenia Working Group of the Psychiatric Genomics C *et al.* LD Score regression distinguishes confounding from polygenicity in genome-wide association studies. *Nat Genet* 2015; **47**(3)**:** 291-295.

7. Speed D, Holmes J, Balding DJ. Evaluating and improving heritability models using summary statistics. *Nat Genet* 2020; **52**(4)**:** 458-462.

8. Grove J, Ripke S, Als TD, Mattheisen M, Walters RK, Won H *et al.* Identification of common genetic risk variants for autism spectrum disorder. *Nat Genet* 2019; **51**(3)**:** 431-444.

9. Demontis D, Walters GB, Athanasiadis G, Walters R, Therrien K, Nielsen TT *et al.* Genome-wide analyses of ADHD identify 27 risk loci, refine the genetic architecture and implicate several cognitive domains. *Nat Genet* 2023; **55**(2)**:** 198-208.

10. de Leeuw CA, Mooij JM, Heskes T, Posthuma D. MAGMA: generalized gene-set analysis of GWAS data. *PLoS Comput Biol* 2015; **11**(4)**:** e1004219.

11. Finucane HK, Bulik-Sullivan B, Gusev A, Trynka G, Reshef Y, Loh PR *et al.* Partitioning heritability by functional annotation using genome-wide association summary statistics. *Nat Genet* 2015; **47**(11)**:** 1228-1235.

12. University College London. National Child Development Study. In: UCL Institute for Education CfLS (ed). 13th release edn2023.

13. Bridges EC, Rayner NW, Mountford HS, Bates TC, Luciano M. Longitudinal Reading Measures and Genome Imputation in the National Child Development Study: Prospects for Future Reading Research. *Twin Res Hum Genet* 2023**:** 1-11.

14. Choi SW, O'Reilly PF. PRSice-2: Polygenic Risk Score software for biobank-scale data. *Gigascience* 2019; **8**(7).

15. Zheng Z, Liu S, Sidorenko J, Wang Y, Lin T, Yengo L *et al.* Leveraging functional genomic annotations and genome coverage to improve polygenic prediction of complex traits within and between ancestries. *Nat Genet* 2024; **56**(5)**:** 767-777.

16. Allentoft ME, Sikora M, Refoyo-Martinez A, Irving-Pease EK, Fischer A, Barrie W *et al.* Population genomics of post-glacial western Eurasia. *Nature* 2024; **625**(7994)**:** 301-311.

17. Irving-Pease EK, Refoyo-Martinez A, Barrie W, Ingason A, Pearson A, Fischer A *et al.* The selection landscape and genetic legacy of ancient Eurasians. *Nature* 2024; **625**(7994)**:** 312-320.

18. Stern AJ, Wilton PR, Nielsen R. An approximate full-likelihood method for inferring selection and allele frequency trajectories from DNA sequence data. *Plos Genet* 2019; **15**(9).

19. Stern AJ, Speidel L, Zaitlen NA, Nielsen R. Disentangling selection on genetically correlated polygenic traits via whole-genome genealogies. *Am J Hum Genet* 2021; **108**(2)**:** 219-239.

20. Barrie W, Yang Y, Irving-Pease EK, Attfield KE, Scorrano G, Jensen LT *et al.* Elevated genetic risk for multiple sclerosis emerged in steppe pastoralist populations. *Nature* 2024; **625**(7994)**:** 321-328.

21. Vuckovic D, Bao EL, Akbari P, Lareau CA, Mousas A, Jiang T *et al.* The Polygenic and Monogenic Basis of Blood Traits and Diseases. *Cell* 2020; **182**(5)**:** 1214-1231 e1211.

22. Barrett JC, Clayton DG, Concannon P, Akolkar B, Cooper JD, Erlich HA *et al.* Genome-wide association study and meta-analysis find that over 40 loci affect risk of type 1 diabetes. *Nat Genet* 2009; **41**(6)**:** 703-707.

23. Verma A, Huffman JE, Rodriguez A, Conery M, Liu M, Ho YL *et al.* Diversity and scale: Genetic architecture of 2068 traits in the VA Million Veteran Program. *Science* 2024; **385**(6706)**:** eadj1182.

24. Feitosa MF, Kraja AT, Chasman DI, Sung YJ, Winkler TW, Ntalla I *et al.* Novel genetic associations for blood pressure identified via gene-alcohol interaction in up to 570K individuals across multiple ancestries. *PLoS One* 2018; **13**(6)**:** e0198166.

25. Wuttke M, Li Y, Li M, Sieber KB, Feitosa MF, Gorski M *et al.* A catalog of genetic loci associated with kidney function from analyses of a million individuals. *Nat Genet* 2019; **51**(6)**:** 957-972.

26. Klarin D, Lynch J, Aragam K, Chaffin M, Assimes TL, Huang J *et al.* Genome-wide association study of peripheral artery disease in the Million Veteran Program. *Nat Med* 2019; **25**(8)**:** 1274-1279.

27. Zhu Z, Guo Y, Shi H, Liu CL, Panganiban RA, Chung W *et al.* Shared genetic and experimental links between obesity-related traits and asthma subtypes in UK Biobank. *J Allergy Clin Immunol* 2020; **145**(2)**:** 537-549.

28. Gill D, Cameron AC, Burgess S, Li X, Doherty DJ, Karhunen V *et al.* Urate, Blood Pressure, and Cardiovascular Disease: Evidence From Mendelian Randomization and Meta-Analysis of Clinical Trials. *Hypertension* 2021; **77**(2)**:** 383-392.

29. Hill WD, Davies NM, Ritchie SJ, Skene NG, Bryois J, Bell S *et al.* Genome-wide analysis identifies molecular systems and 149 genetic loci associated with income. *Nat Commun* 2019; **10**(1)**:** 5741.

**
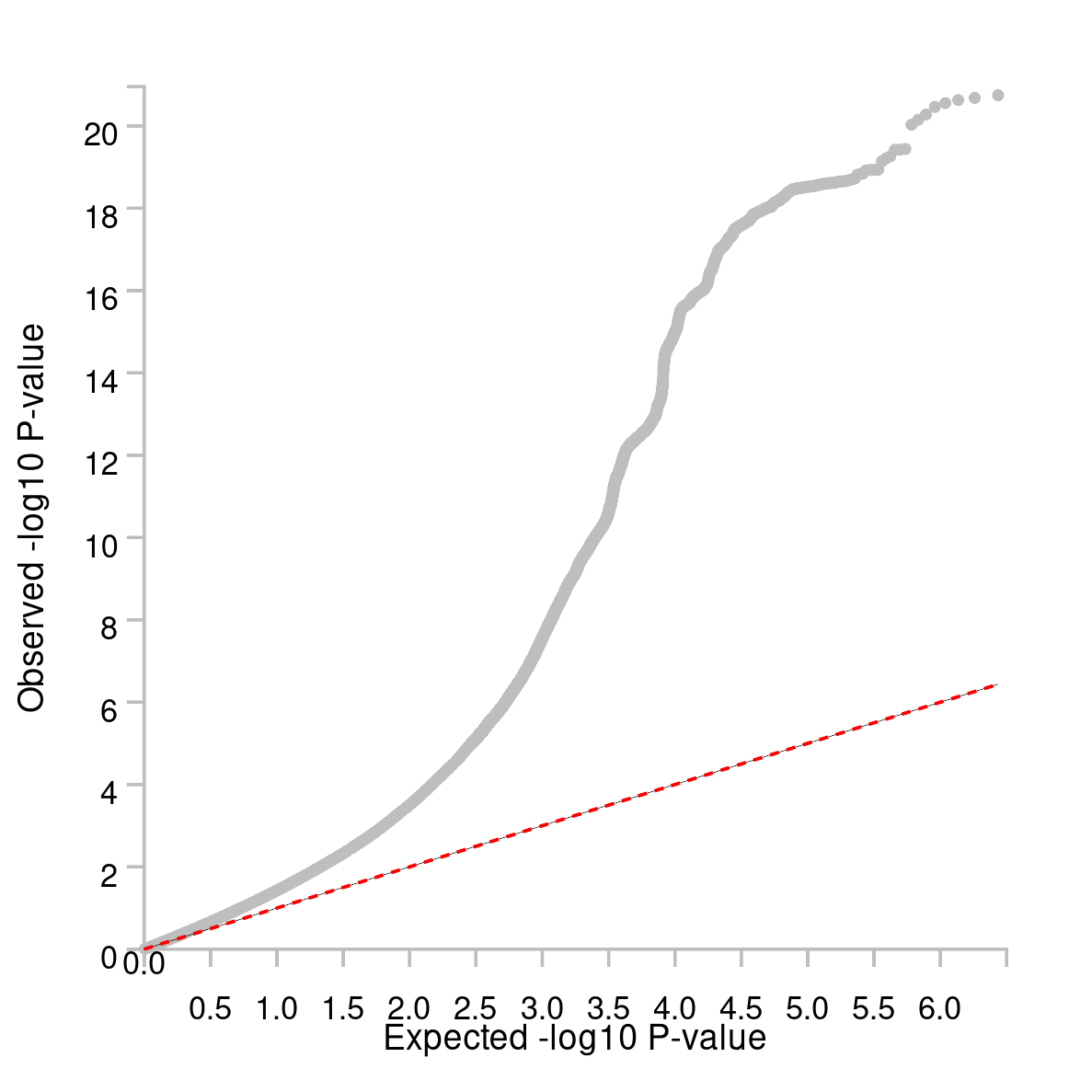
Supplementary Figures**

**Figure S1**: Quantile-Quantile (Q-Q) plot showing expected versus observed -log10 P-values for each variant from multivariate GWAS of dyslexia, represented by grey dots. The dashed red line shows the null hypothesis. The plot indicates moderate P value inflation, and is consistent with a high powered GWAS of a highly polygenic trait.


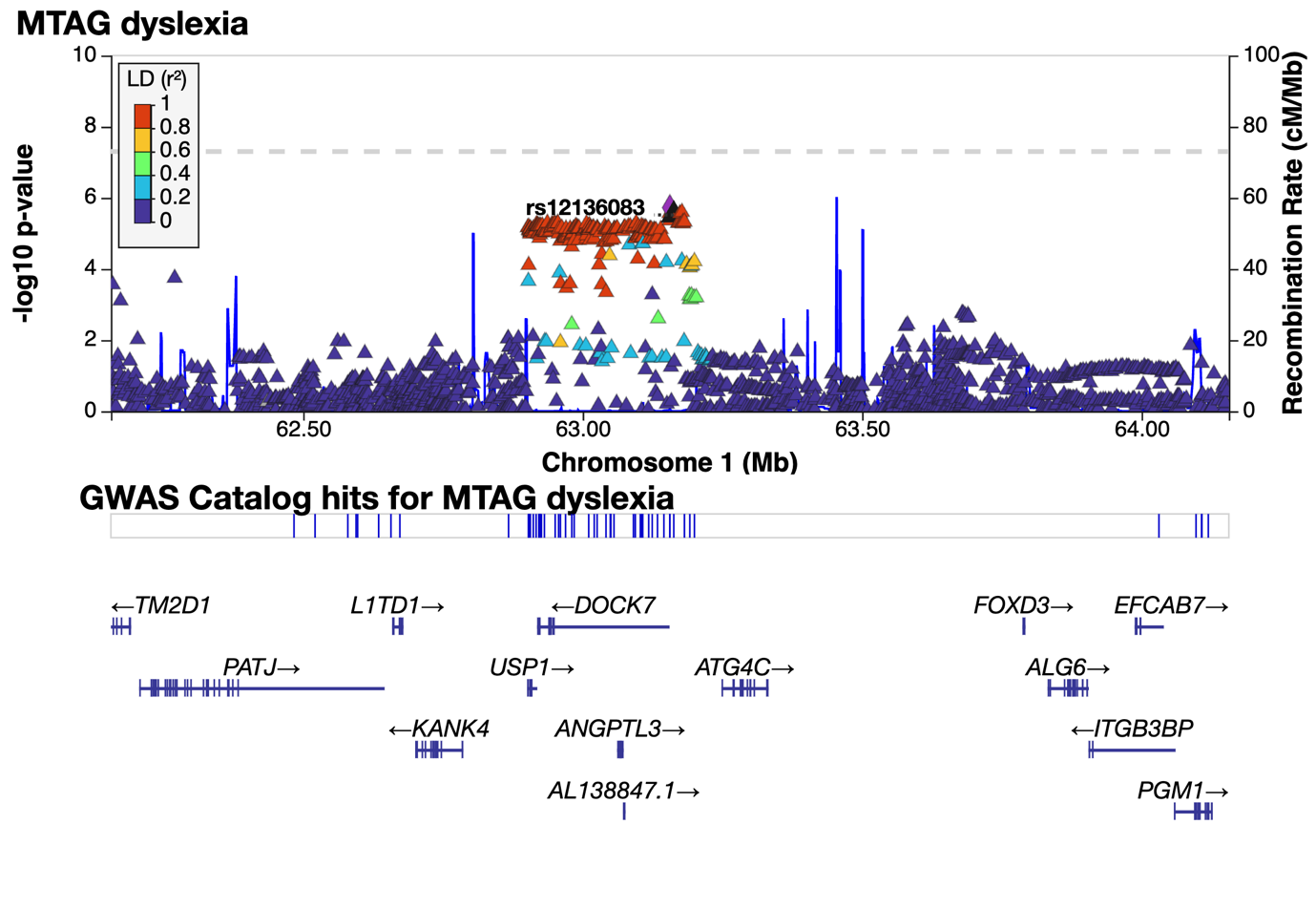


**Figure S2:** LocusZoom of suggestive significance chr1:62900811-63199936 at P = 1.96x10^-6^ with rs1168114 (1:63156043 G/A). This region overlaps completely with the locus previously reported by Eising et al 2022, however lead SNP has changed from rs11208009 to rs1168114.

**
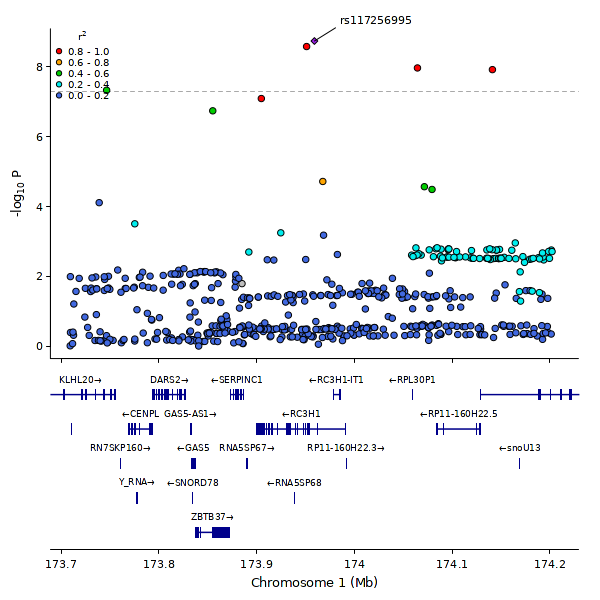
**

**Figure S3**: LocusZoom of region associated with dyslexia; region chr1q25.1 - rs117256995.


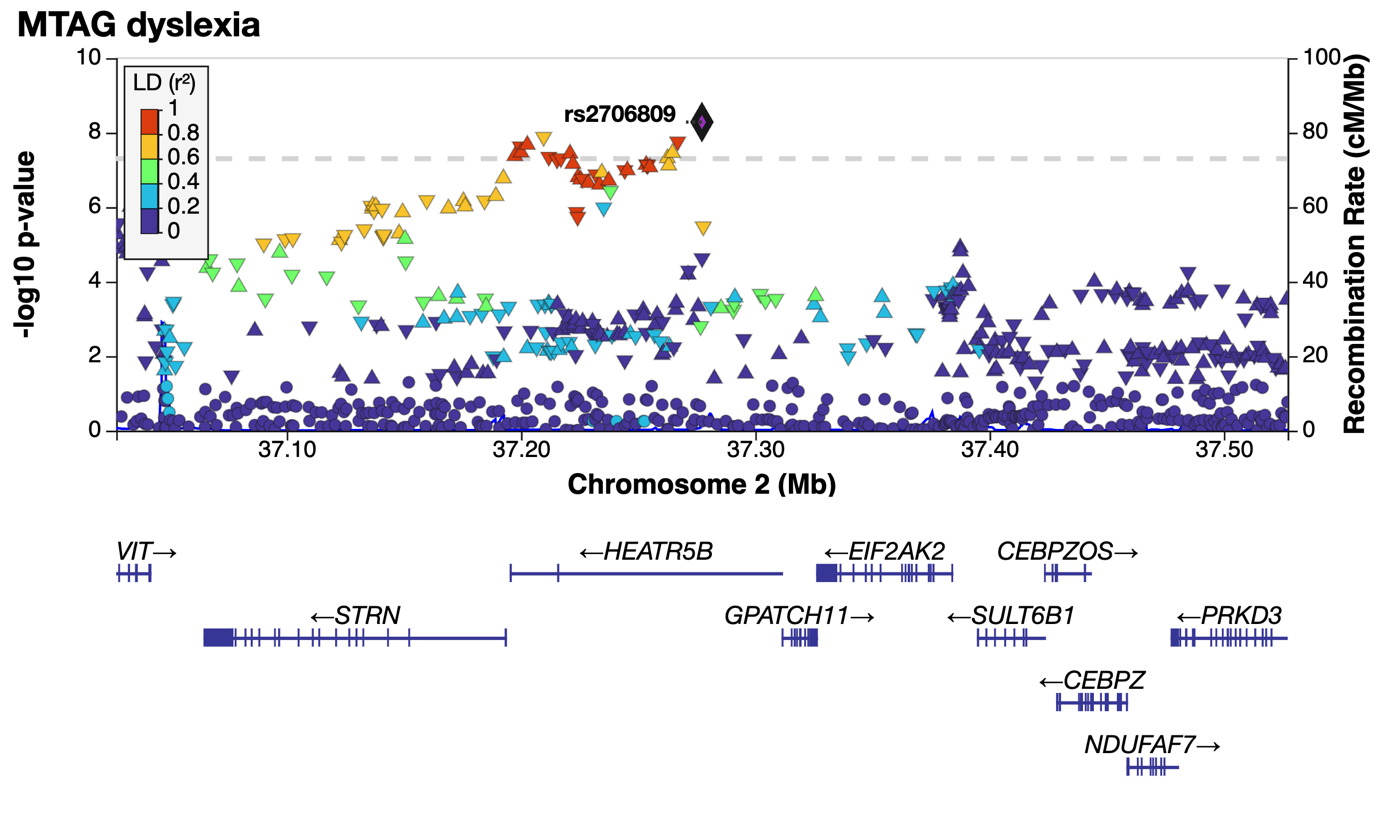


**Figure S4**: LocusZoom of region associated with dyslexia; region chr2p22.2 - rs2706809.


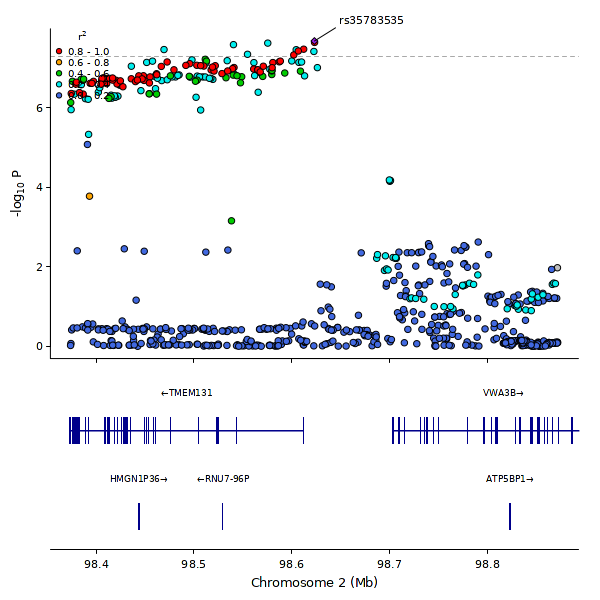


**Figure S5**: LocusZoom of region associated with dyslexia; region chr2q11.2 - rs35783535.


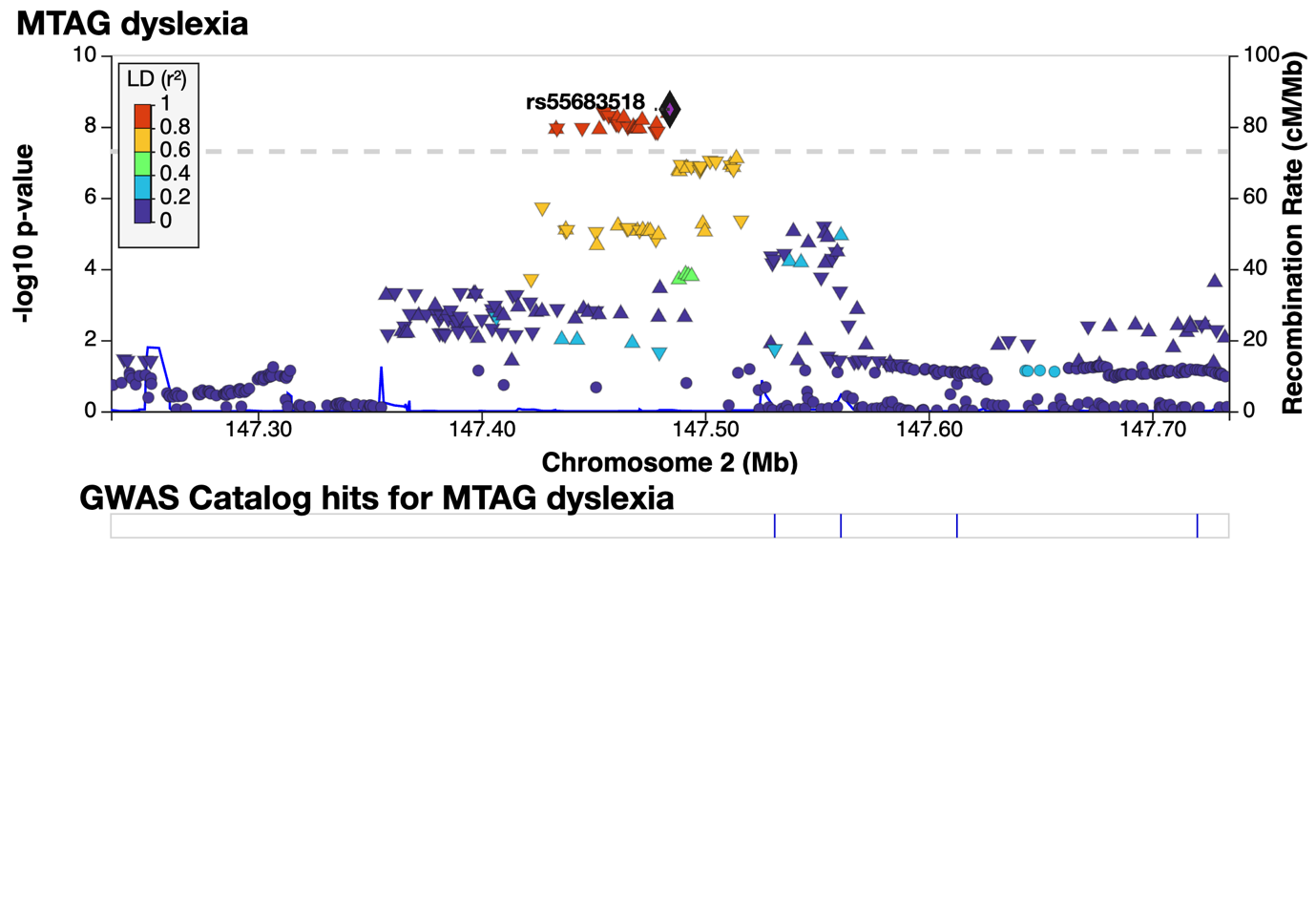


**Figure S6**: LocusZoom of region associated with dyslexia; region chr2q22.3 - rs55683518


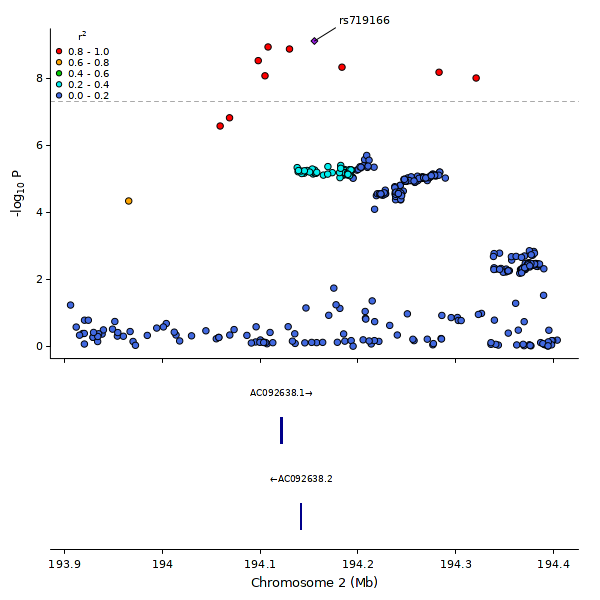


**Figure S7**: LocusZoom of region associated with dyslexia; region chr2q32.3 - rs719166.


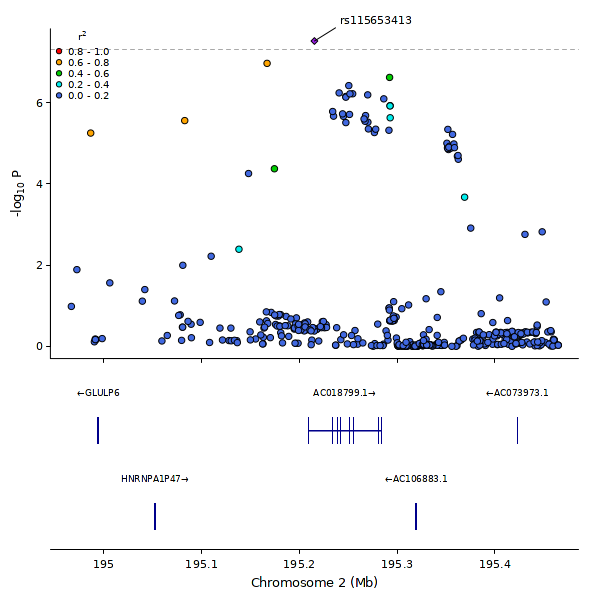


**Figure S8**: LocusZoom of region associated with dyslexia; region chr2q32.3 - rs115653413.


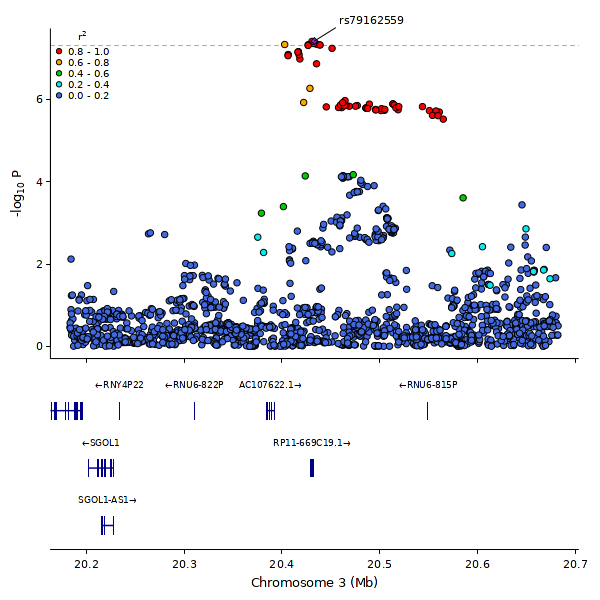


**Figure S9**: LocusZoom of region associated with dyslexia; region chr3p24.3 - rs79162559.


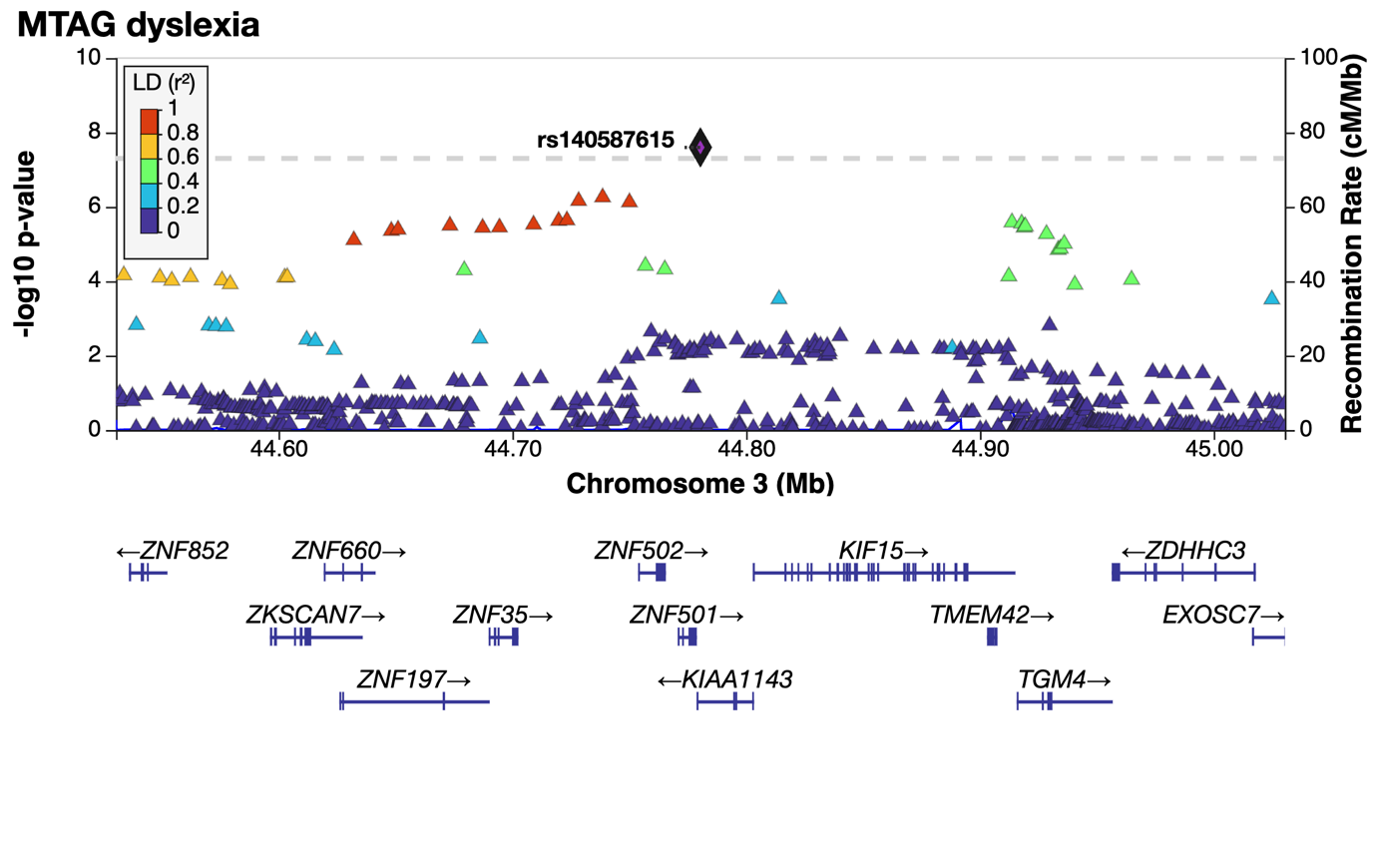


**Figure S10**: LocusZoom of novel region associated with dyslexia; region chr3p21.31 - rs140587615.

**
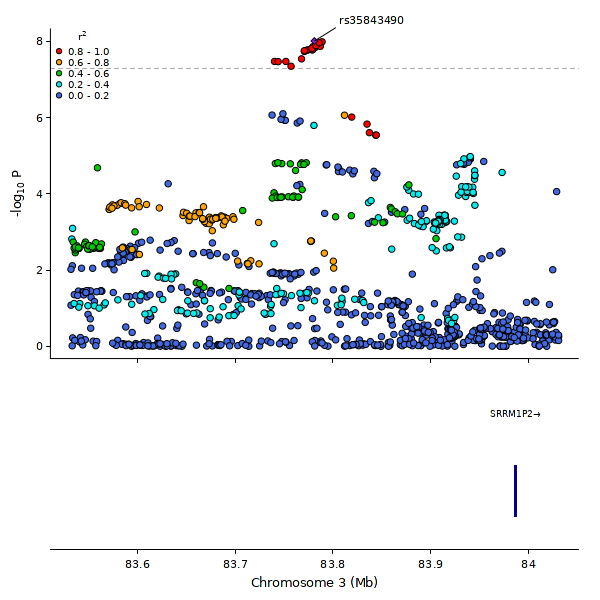
**

**Figure S11**: LocusZoom of region associated with dyslexia; region chr3p12.1 - rs35843490.

**
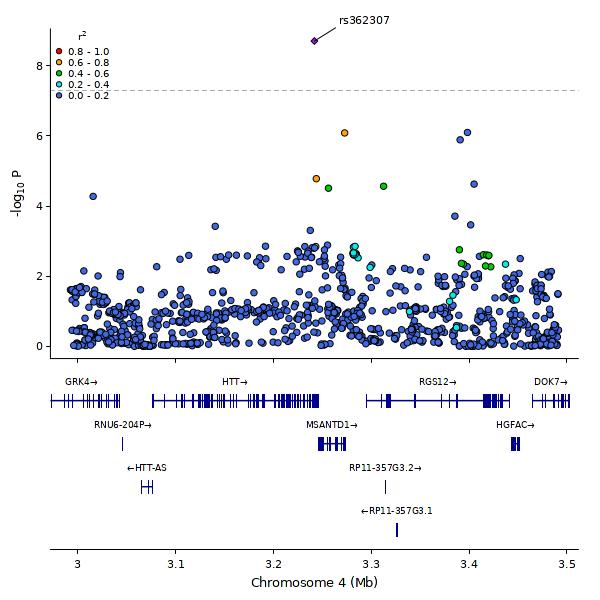
**

**Figure S12**: LocusZoom of novel region associated with dyslexia; region chr4p16.3 - rs362307.

**
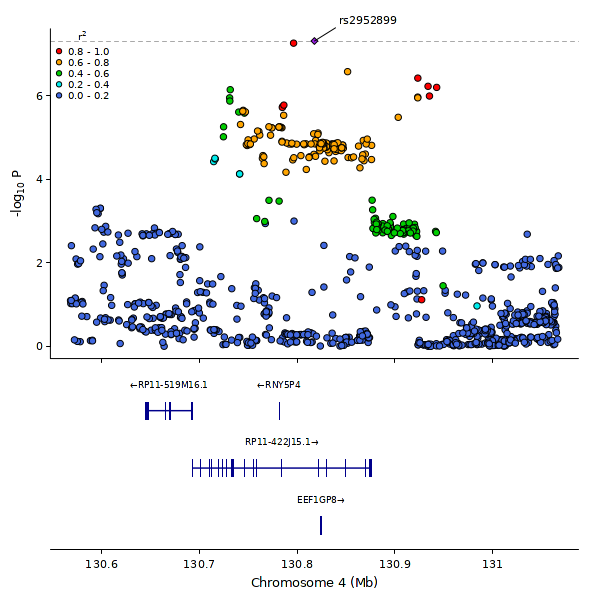
**

**Figure S13**: LocusZoom of novel region associated with dyslexia; region chr4q28.2 - rs2952899.


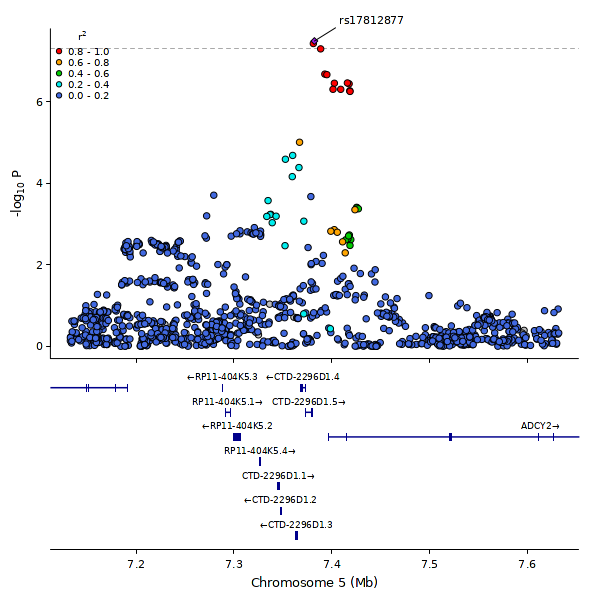


**Figure S14**: LocusZoom of novel region associated with dyslexia; region chr5p15.31 - rs17812877.


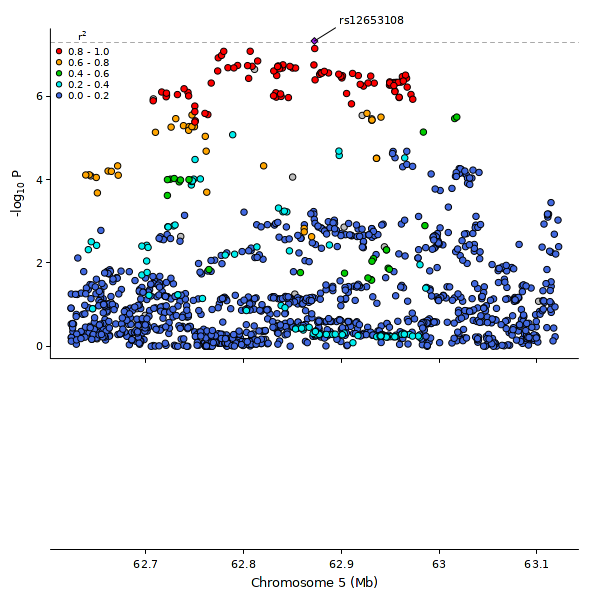


**Figure S15**: LocusZoom of novel region associated with dyslexia; region chr5q12.1 - rs12653108.

**
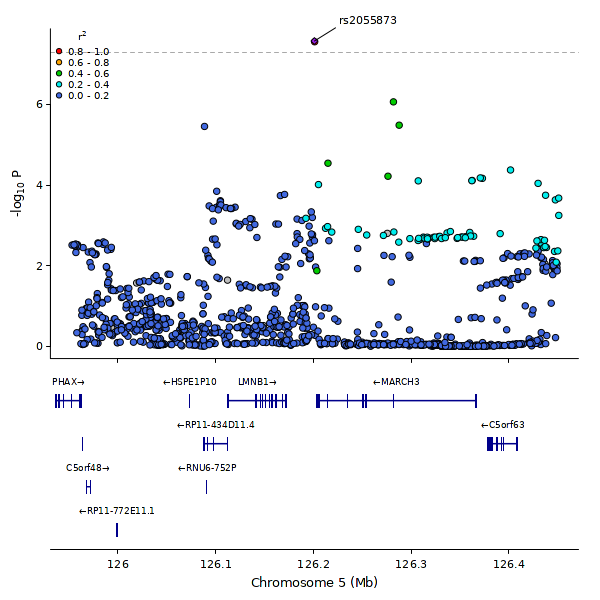
**

**Figure S16**: LocusZoom of novel region associated with dyslexia; region chr5q23.2 - rs2055873.


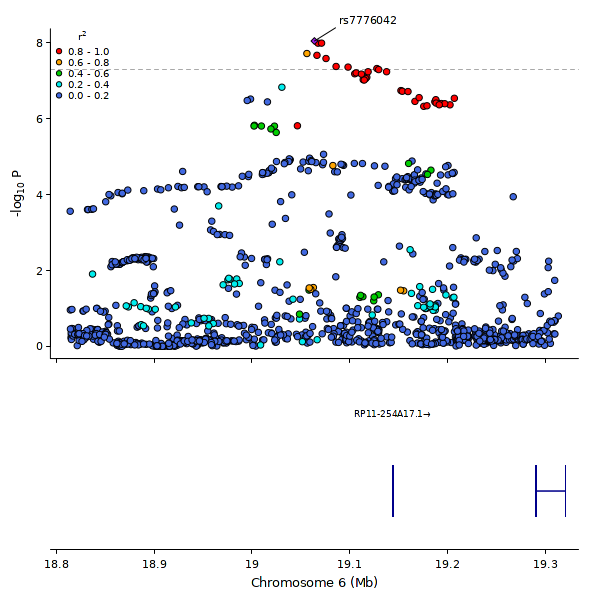


**Figure S17**: LocusZoom of novel region associated with dyslexia; region chr6p22.3 - rs7776042.


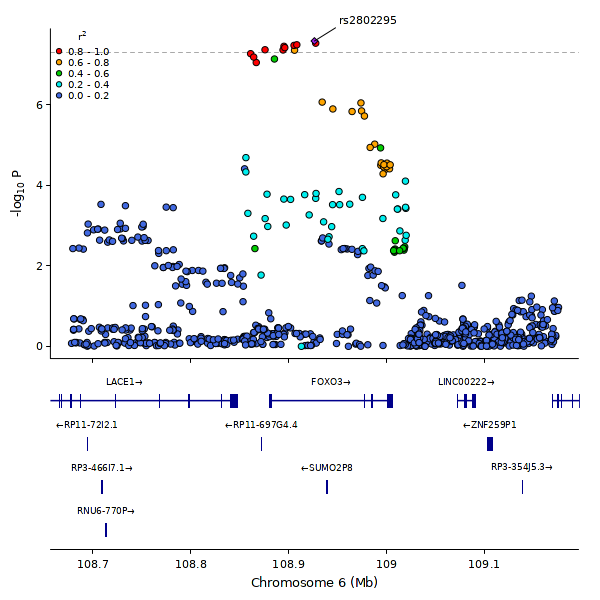


**Figure S18**: LocusZoom of region associated with dyslexia; region chr6q21 - rs2802295.

**
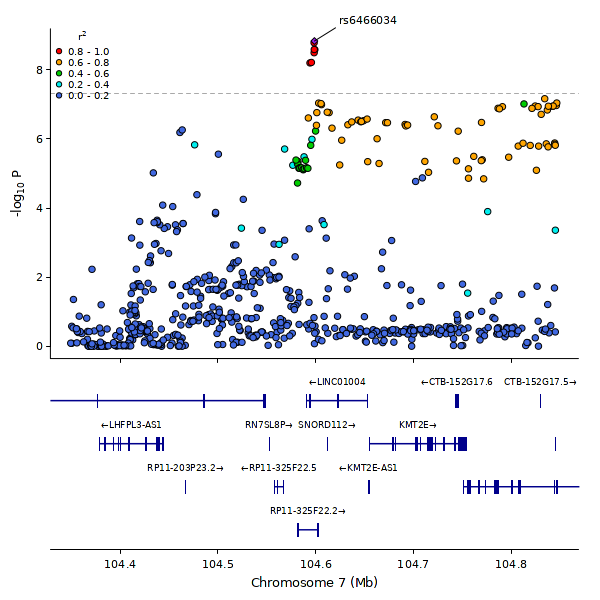
**

**Figure S19**: LocusZoom of region associated with dyslexia; region chr7q22.3 - rs6466034.


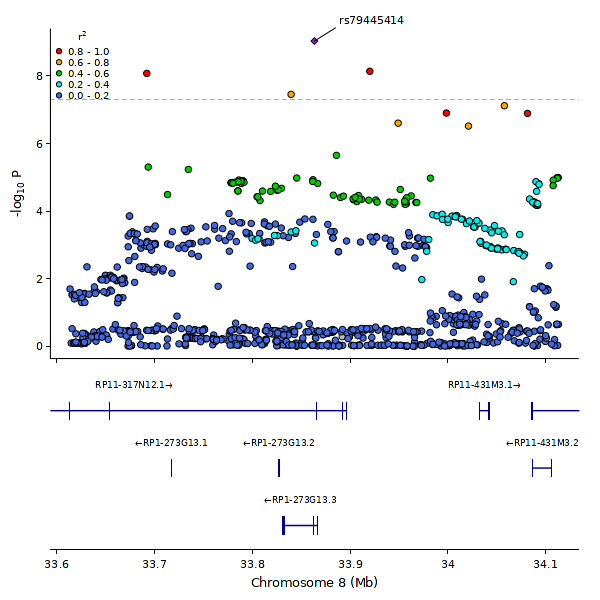


**Figure S20**: LocusZoom of novel region associated with dyslexia; region chr8p12 - rs79445414.


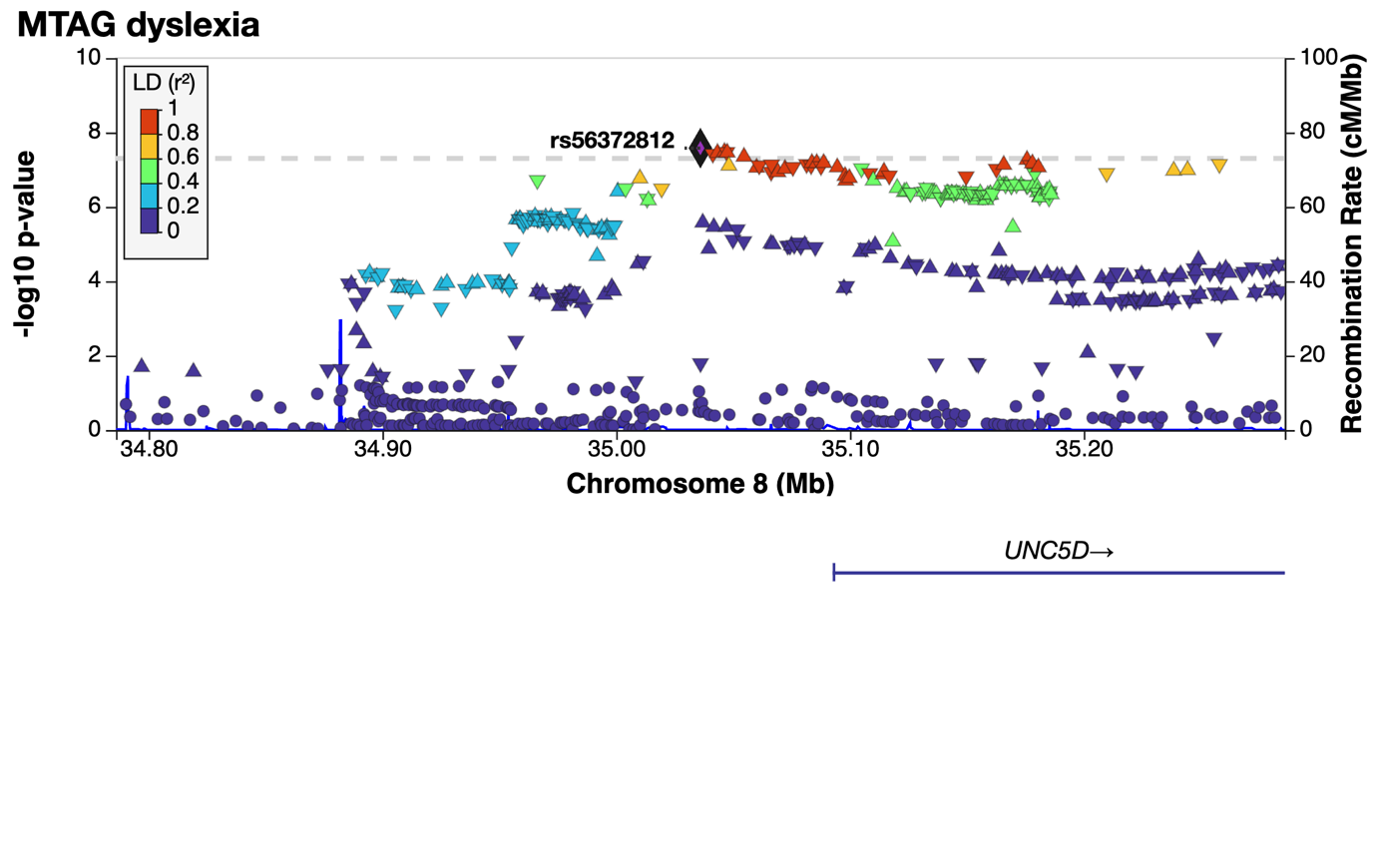


**Figure S21**: LocusZoom of novel region associated with dyslexia; region chr8p12 - rs56372812.

**
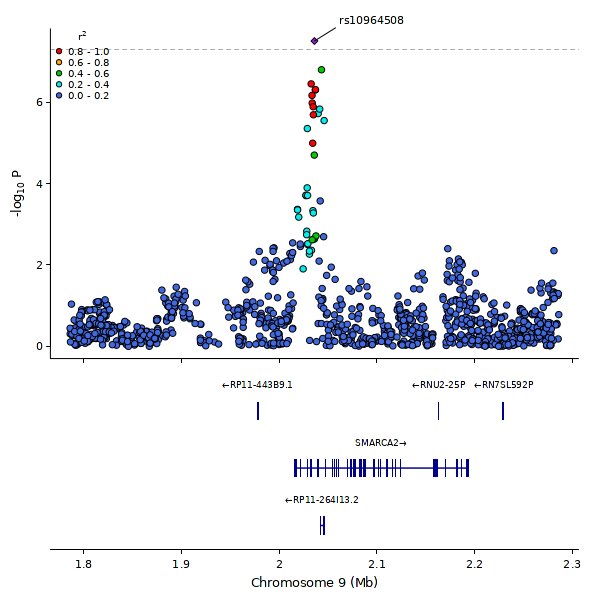
**

**Figure S22**: LocusZoom of region associated with dyslexia; region chr9p24.3 - rs10964508.


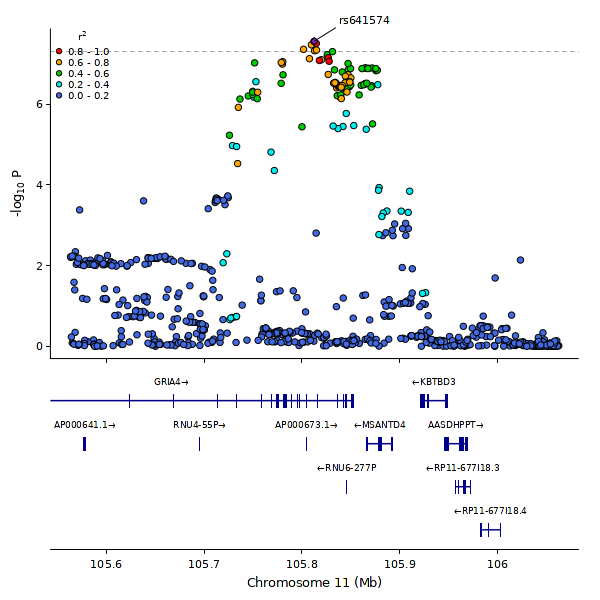


**Figure S23**: LocusZoom of novel region associated with dyslexia; region chr11q22.3 - rs641574.

**
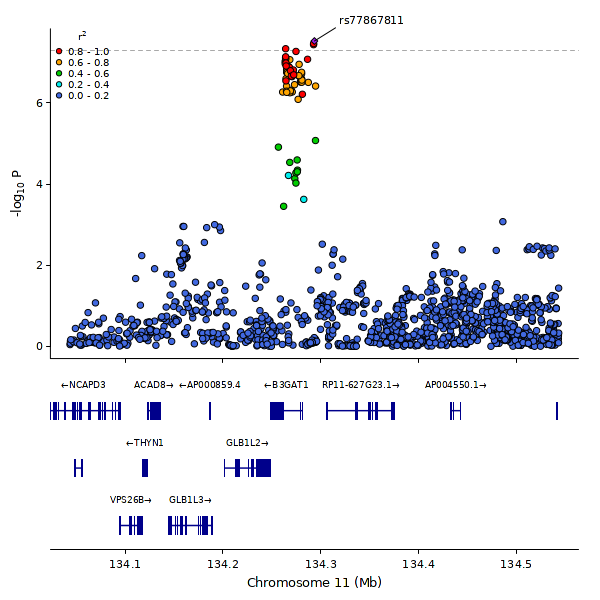
**

**Figure S24**: LocusZoom of region associated with dyslexia; region chr11q25 - rs77867811.

**
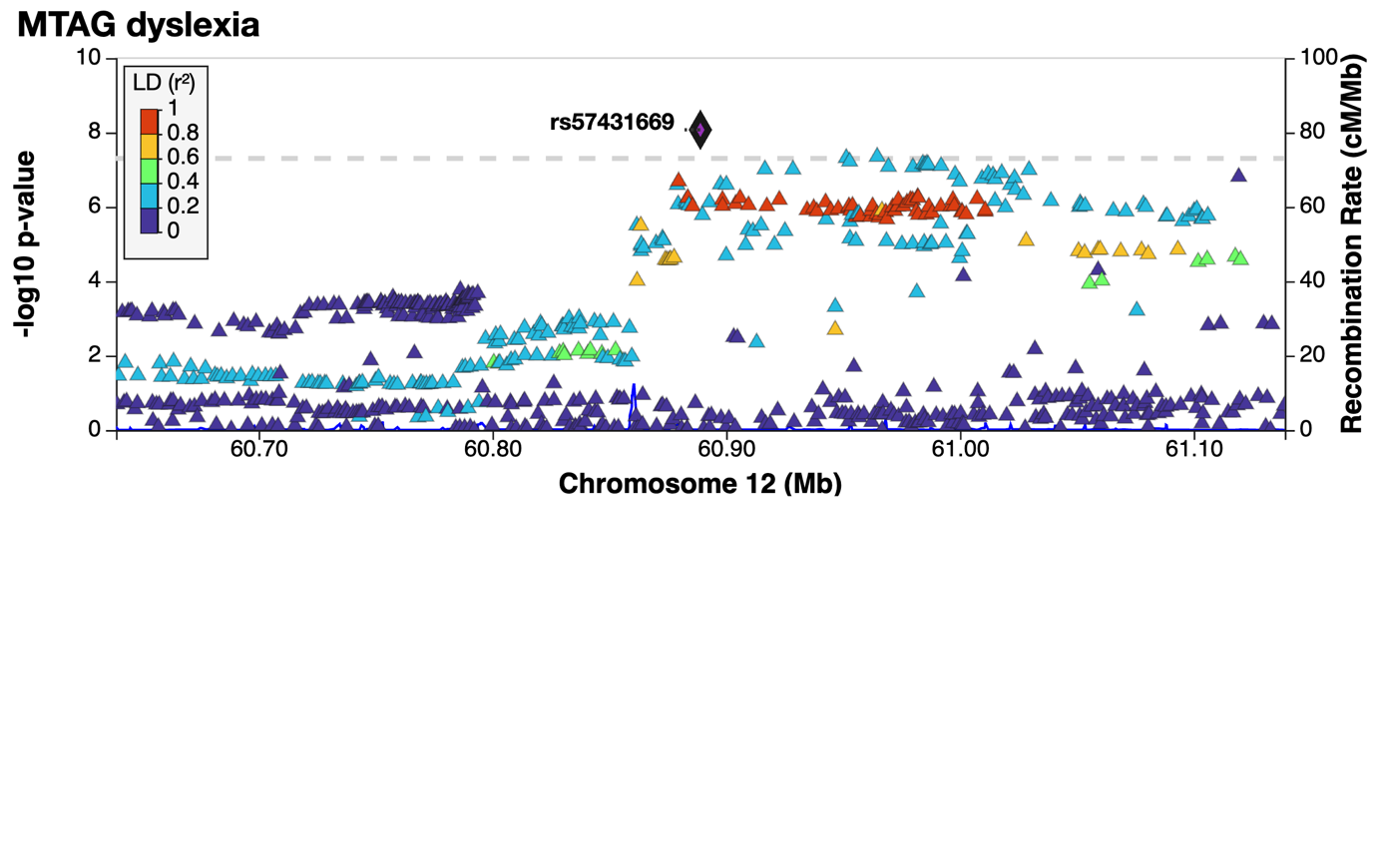
**

**Figure S25**: LocusZoom of region associated with dyslexia; region chr12q14.1 - rs57431669.

**
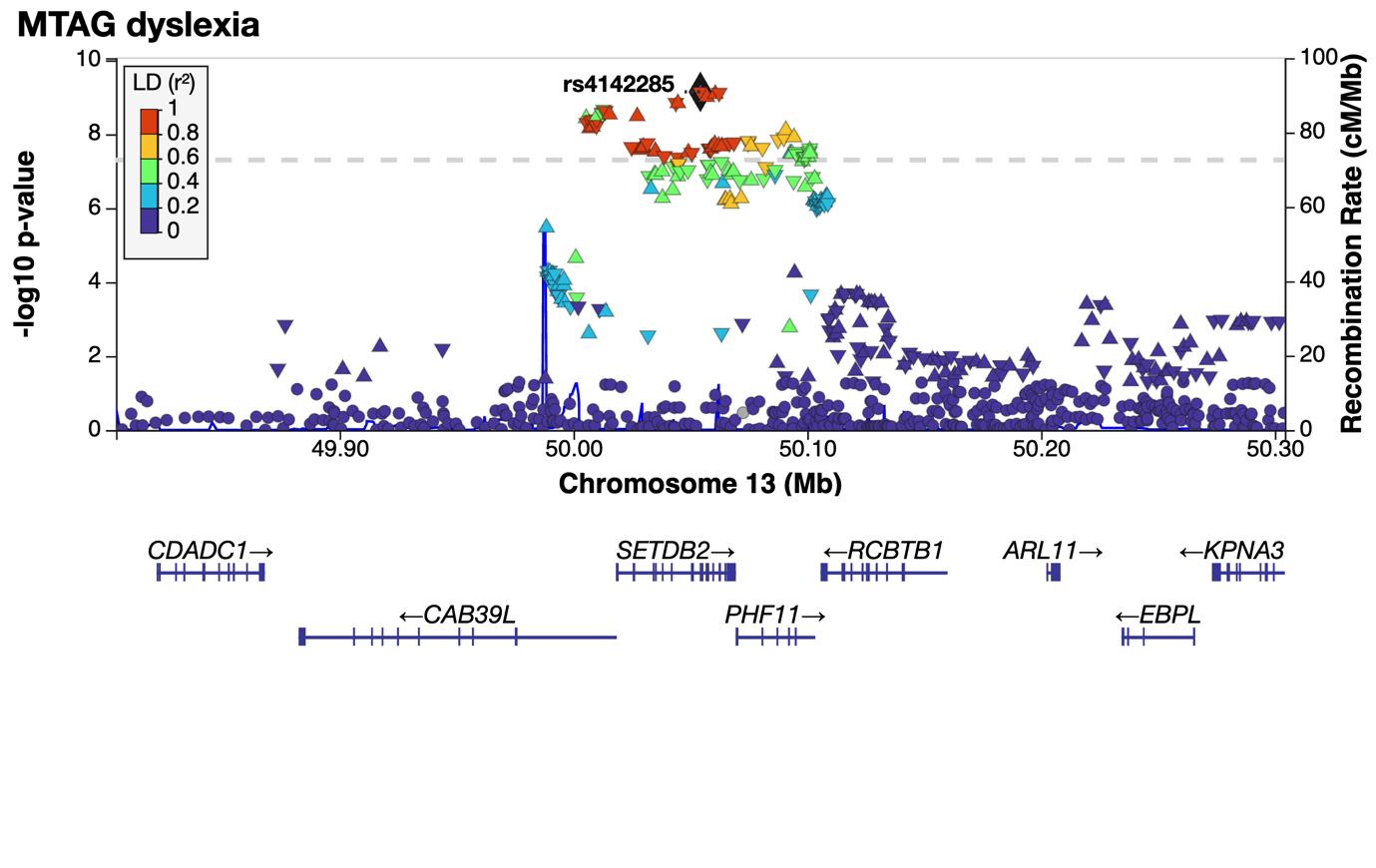
**

**Figure S26**: LocusZoom of region associated with dyslexia; region chr13q14.2 - rs4142285.


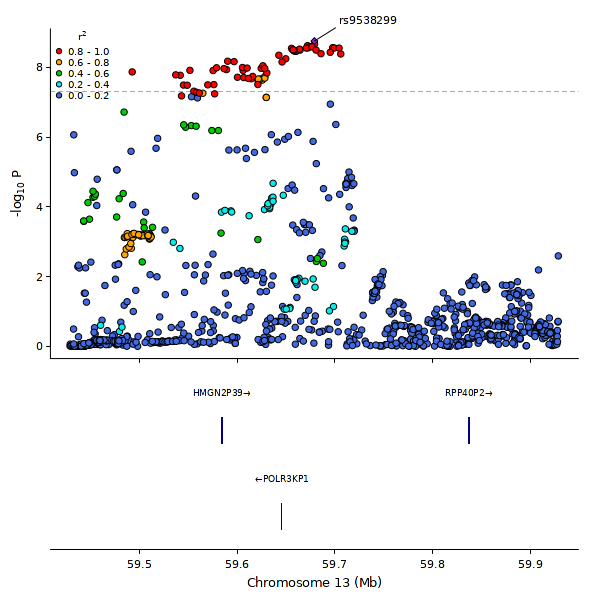


**Figure S27**: LocusZoom of region associated with dyslexia; region chr13q21.2 - rs9538299.


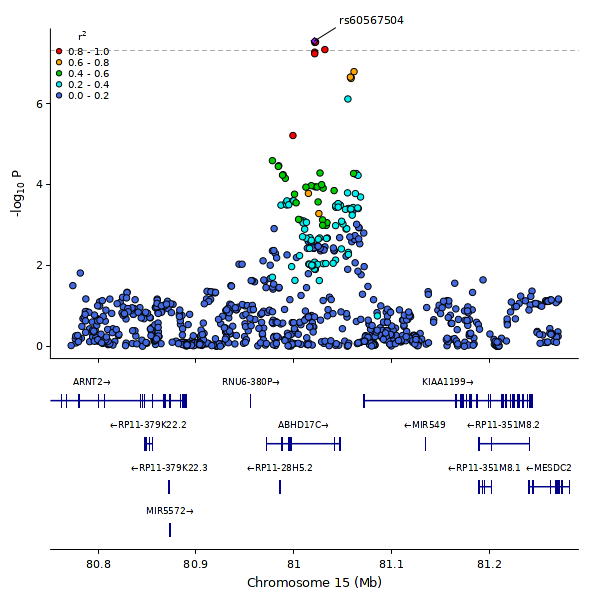


**Figure S28**: LocusZoom of novel region associated with dyslexia; region chr15q25.1 - rs60567504.


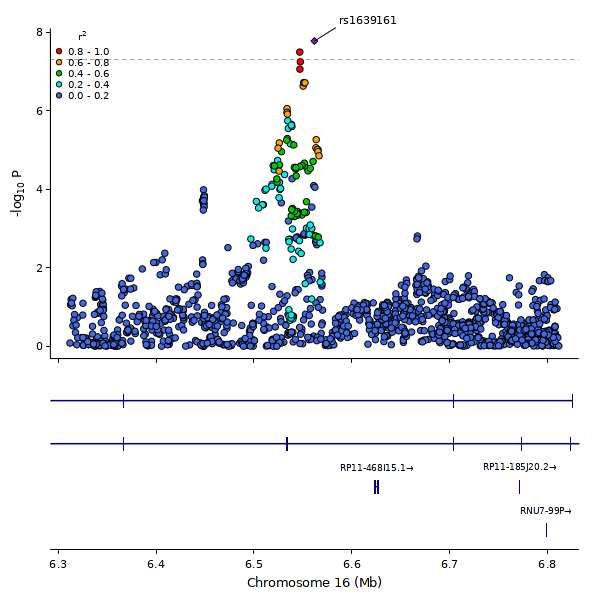


**Figure S29**: LocusZoom of region associated with dyslexia; region chr16p13.3 - rs1639161.


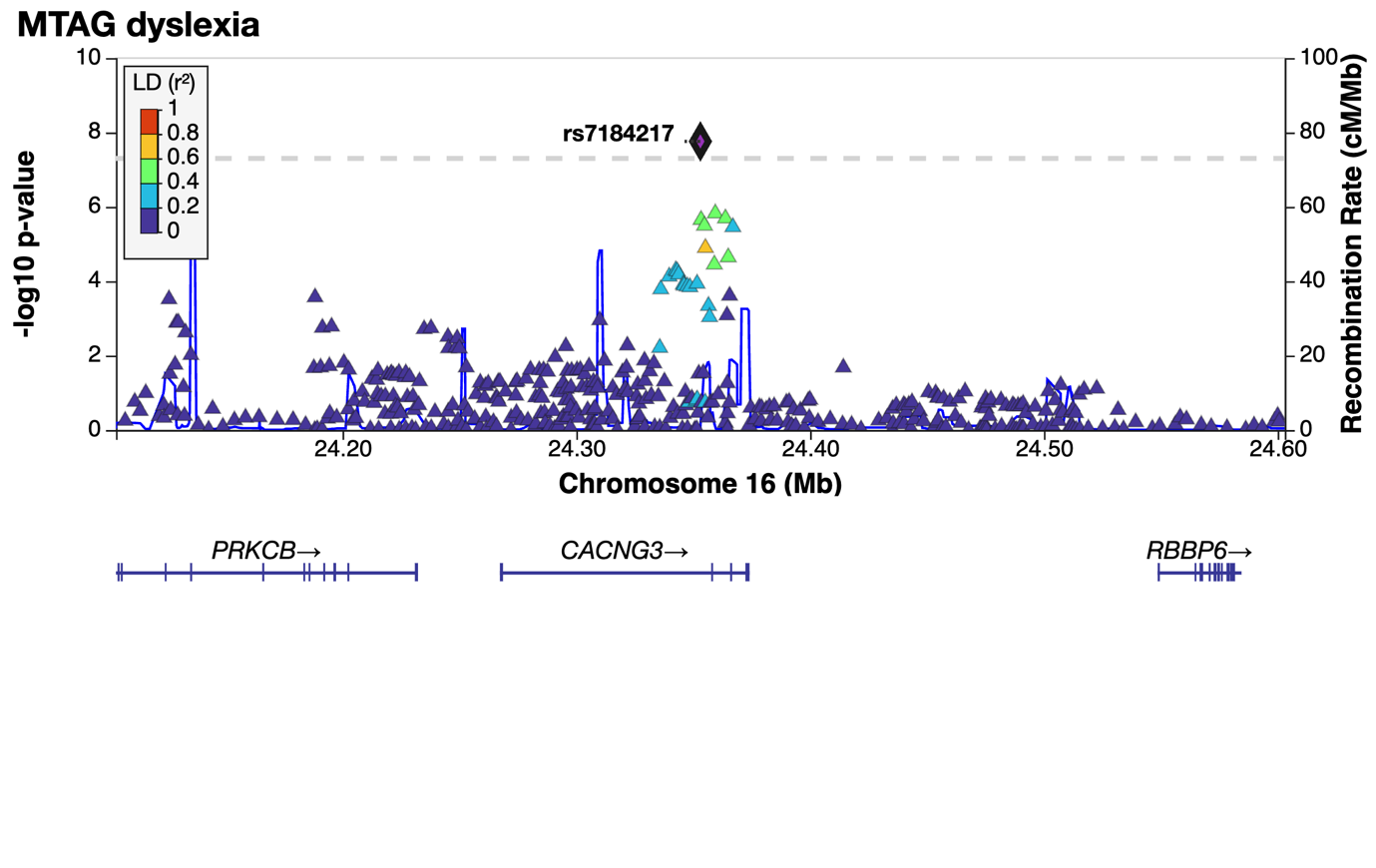


**Figure S30**: LocusZoom of novel region associated with dyslexia; region chr16p12.1 - rs7184217.


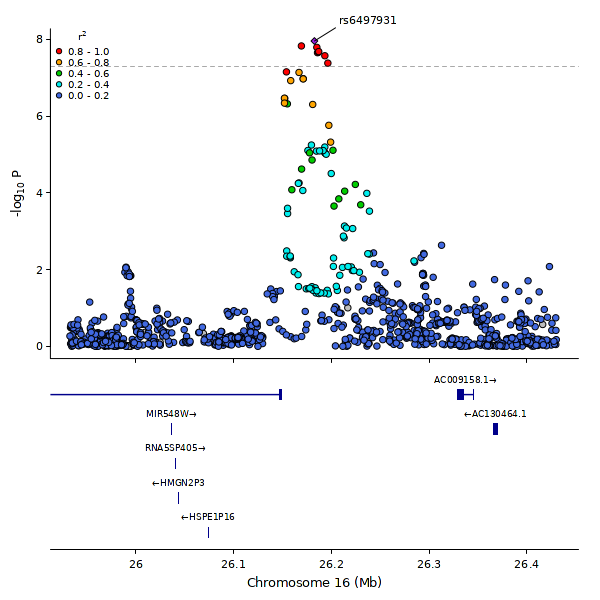


**Figure S31**: LocusZoom of region associated with dyslexia; region chr16p12.1 - rs6497931.


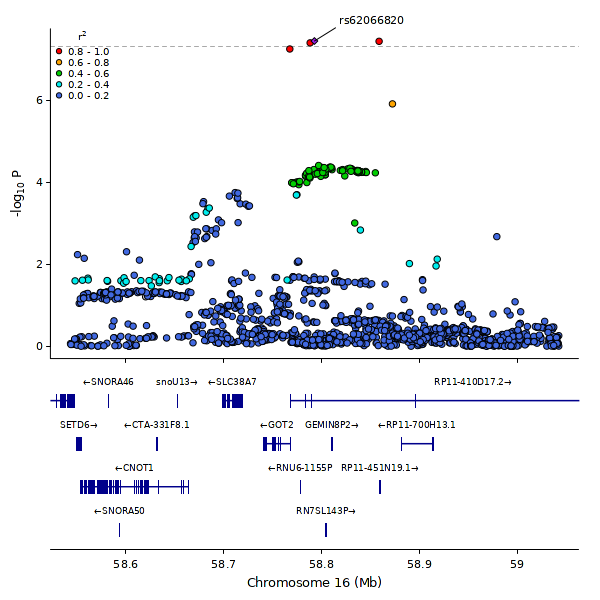


**Figure S32**: LocusZoom of region associated with dyslexia; region chr16q21 - rs62066820.

**
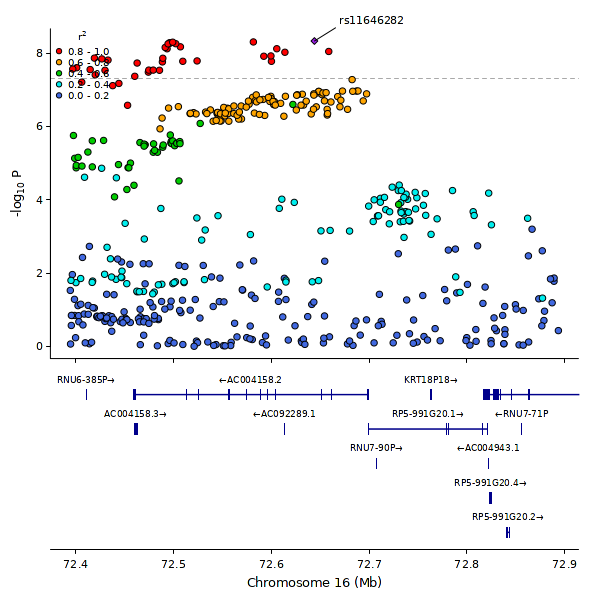
**

**Figure S33:** LocusZoom of region associated with dyslexia; region chr16q22.2 - rs11646282.


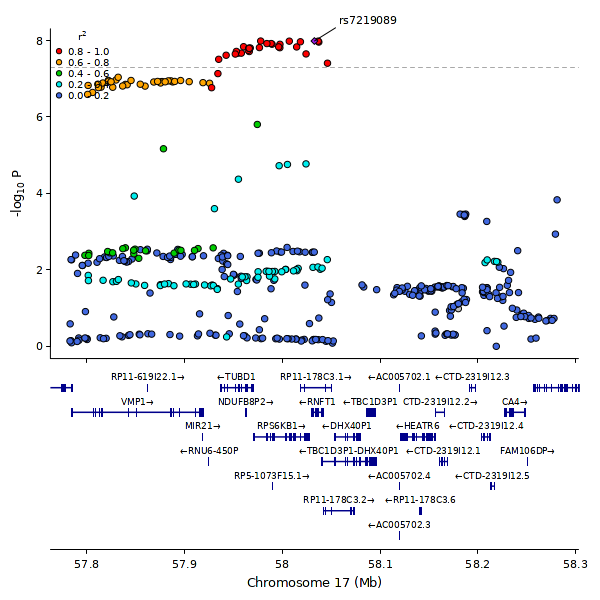


**Figure S34**: LocusZoom of region associated with dyslexia; region chr17q23.1 - rs7219089.


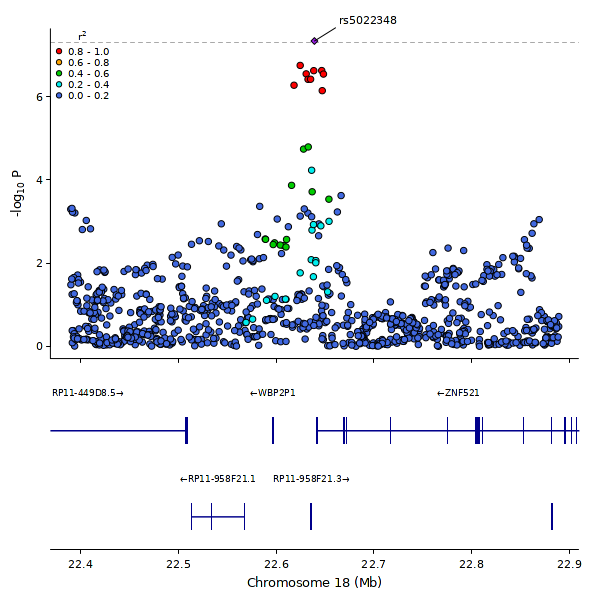


**Figure S35**: LocusZoom of novel region associated with dyslexia; region chr18q11.2 - rs5022348.

.


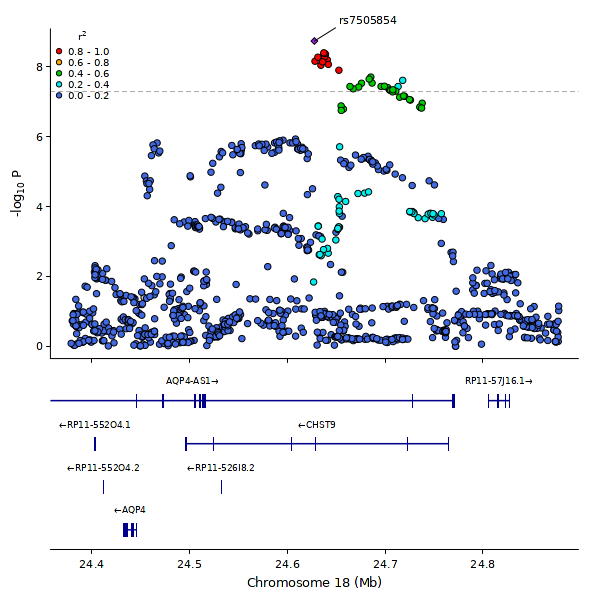


**Figure S36**: LocusZoom of region associated with dyslexia; region chr18q11.2 - rs7505854.


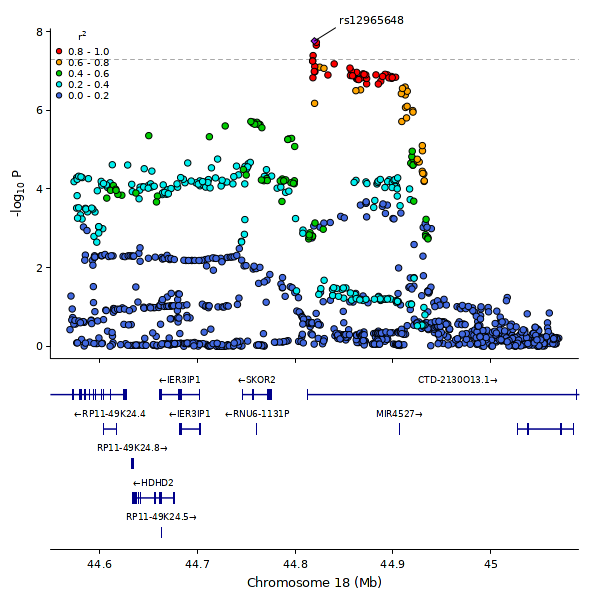


**Figure S37**: LocusZoom of regions associated with dyslexia; region chr18q21.1 - rs12965648.


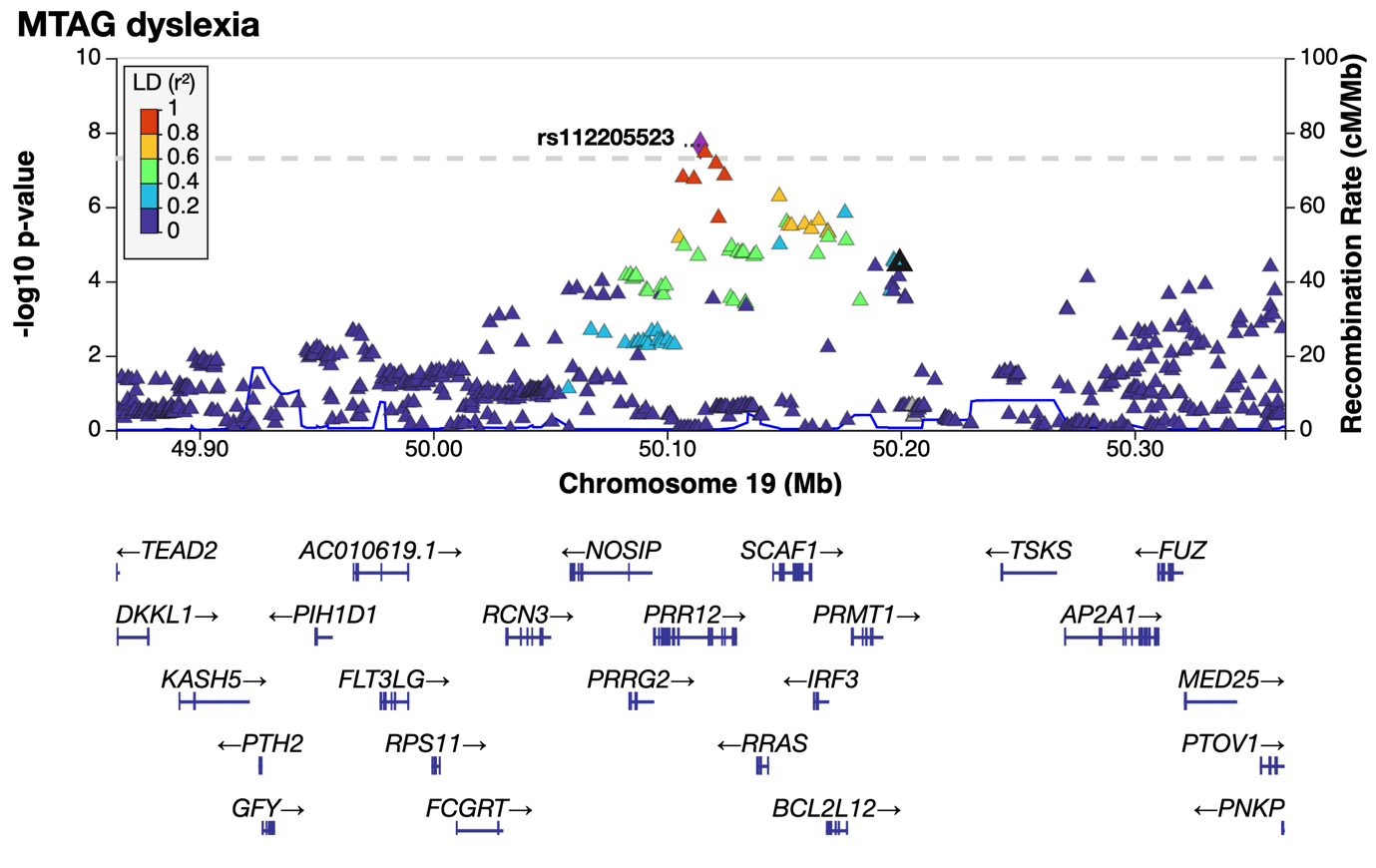


**Figure S38:** LocusZoom of region associated with dyslexia; region chr19q13.33 - rs112205523.

**
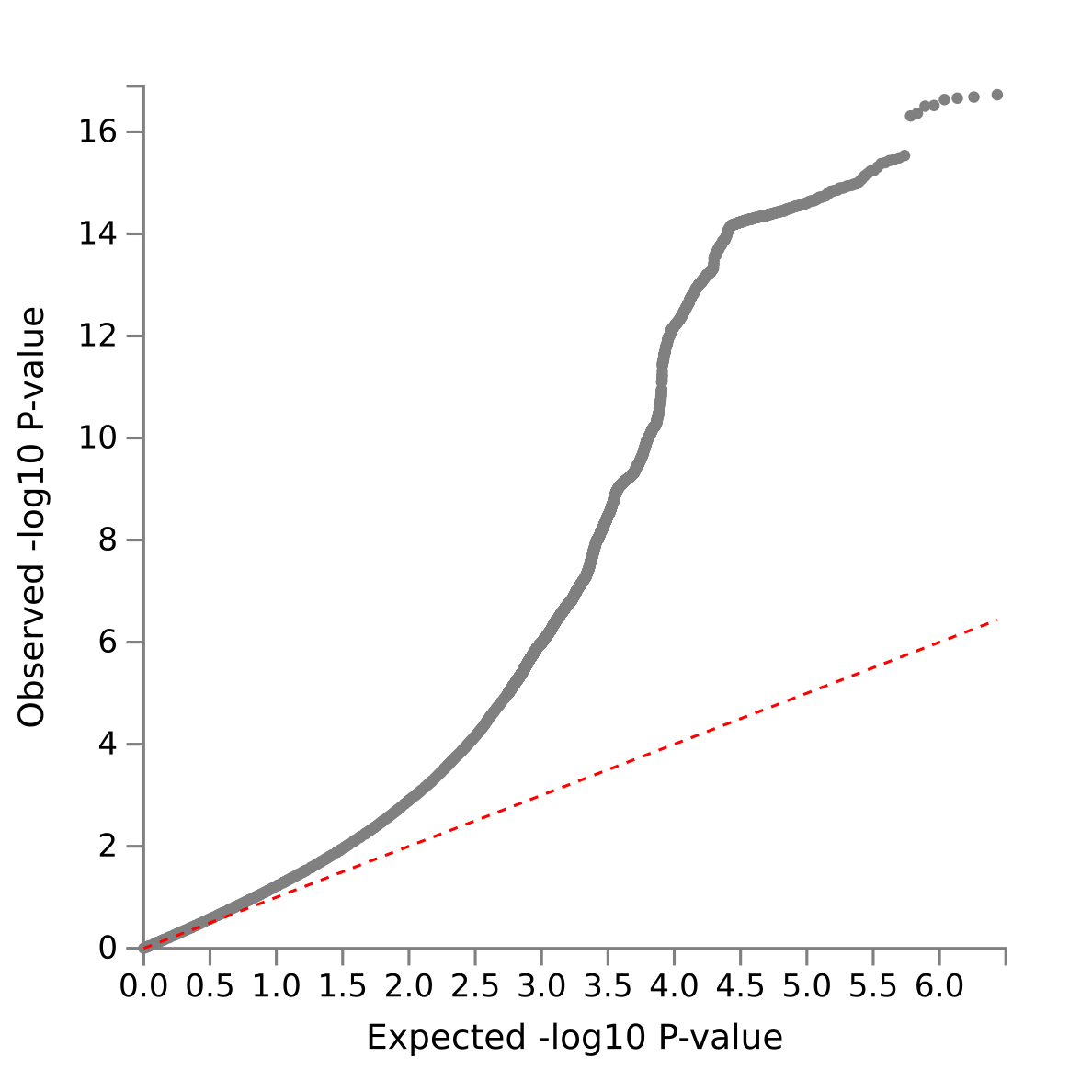
Figure S39**: Quantile-Quantile (Q-Q) plot showing expected versus observed -log10 P-values for each variant from multivariate GWAS of reading ability, represented by grey dots. The dashed red line shows the null hypothesis. The plot indicates the absence of uncorrected population stratification and is consistent with a highly polygenic trait.

**
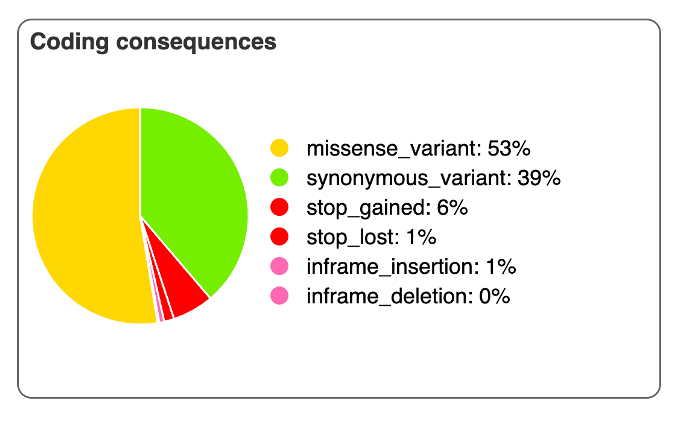
**
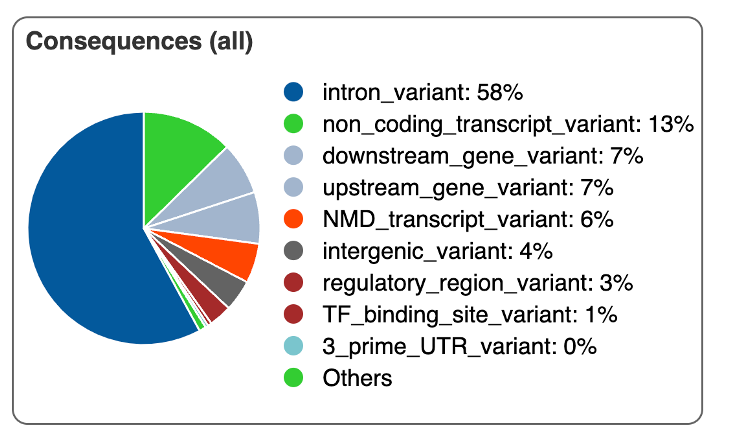


B

A

**Figure S40:** Variant consequences annotated by Variant Effect Predictor (VEP) showing A) positional annotation of the SNP, and B) for SNPs that fell within a coding region, the functional consequence of that variant associated with dyslexia.

**
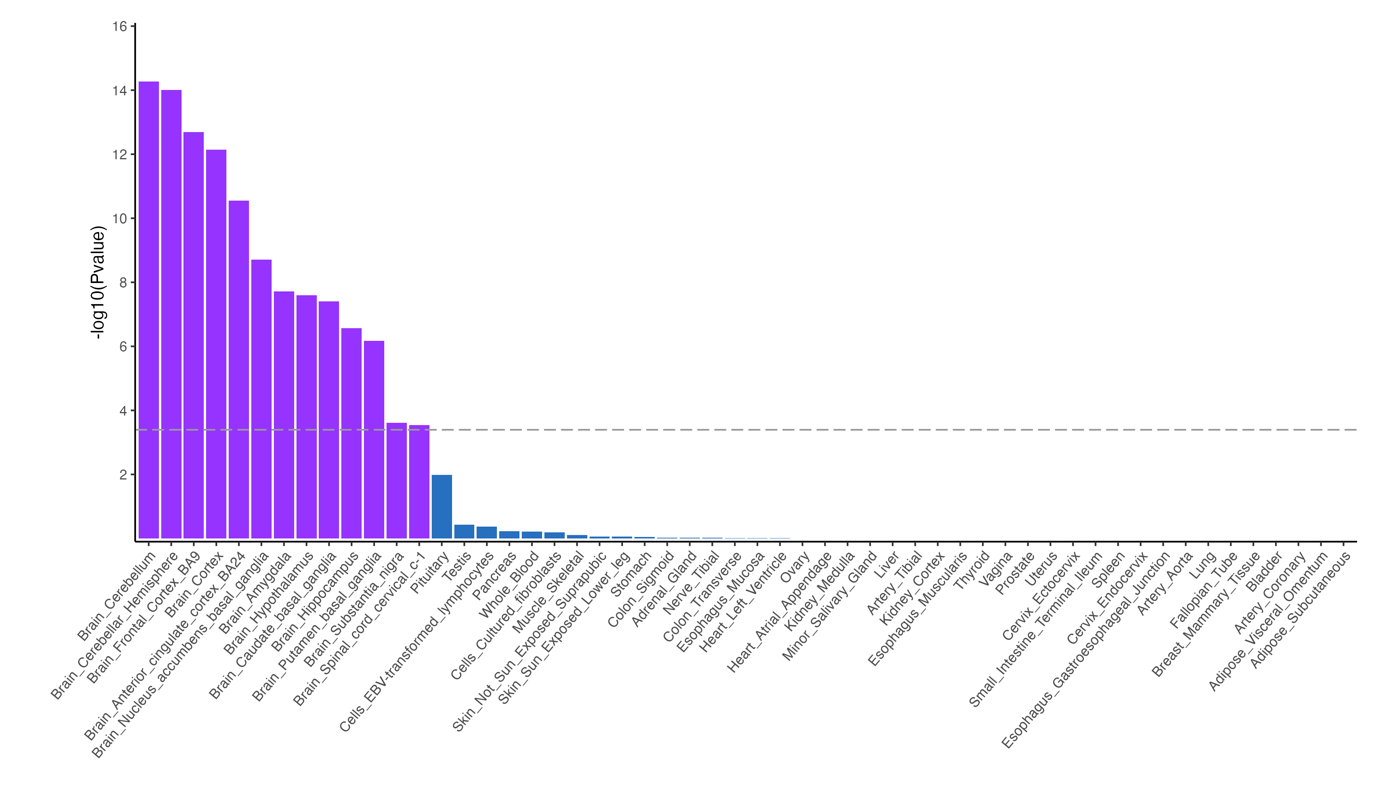
**

**Figure S41:** -log_10_ P values from a MAGMA gene-property analysis of dyslexia multivariate GWAS showing enrichment of expression in brain regions. The dashed line indicates the Bonferroni correction for 124 tests to P < 4.032 x 10^-4^.

**
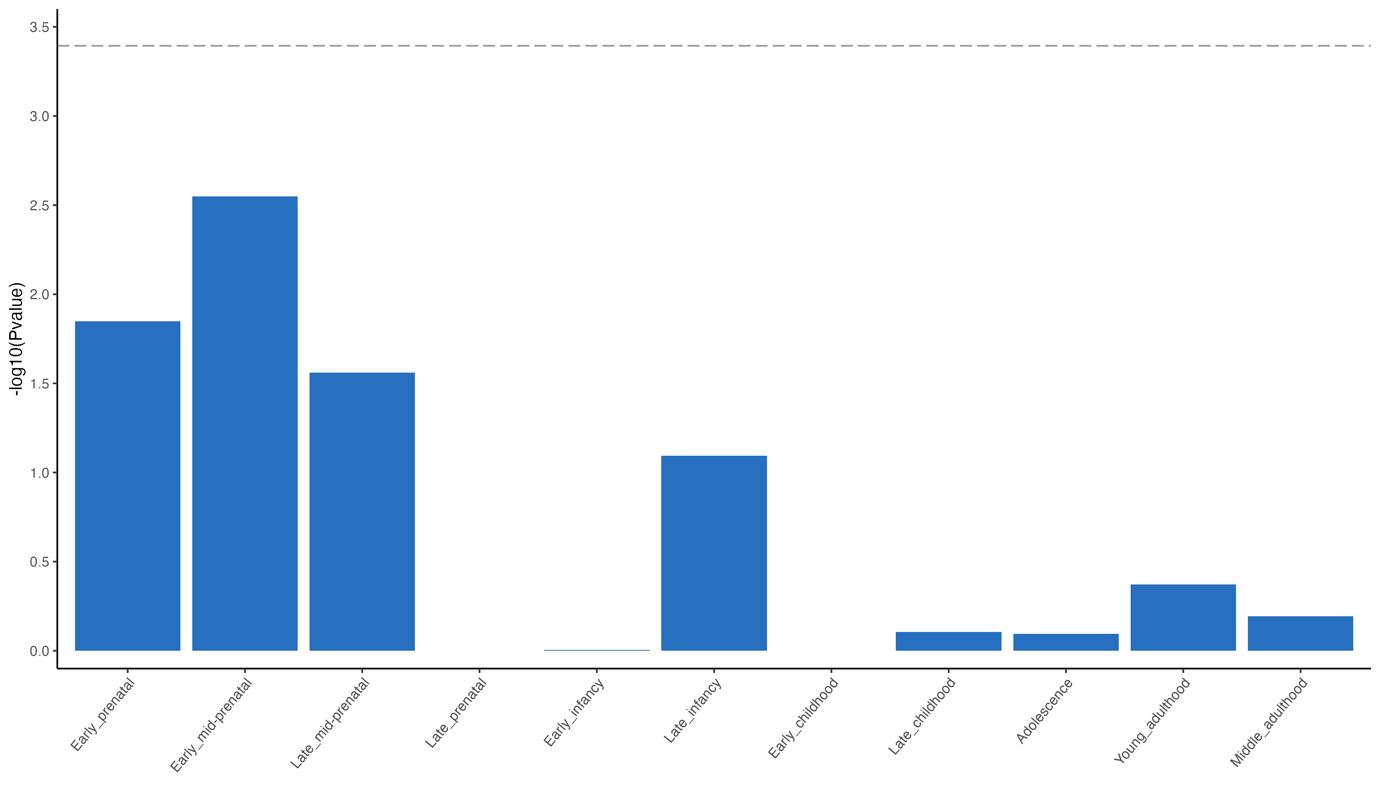
**

**Figure S42:** -log_10_ P values from a MAGMA gene property analysis of dyslexia multivariate GWAS showing enrichment through 11 developmental stages from BrainSpan. The dashed line indicates the Bonferroni correction for 124 tests to P < 4.032 x 10^-4^.


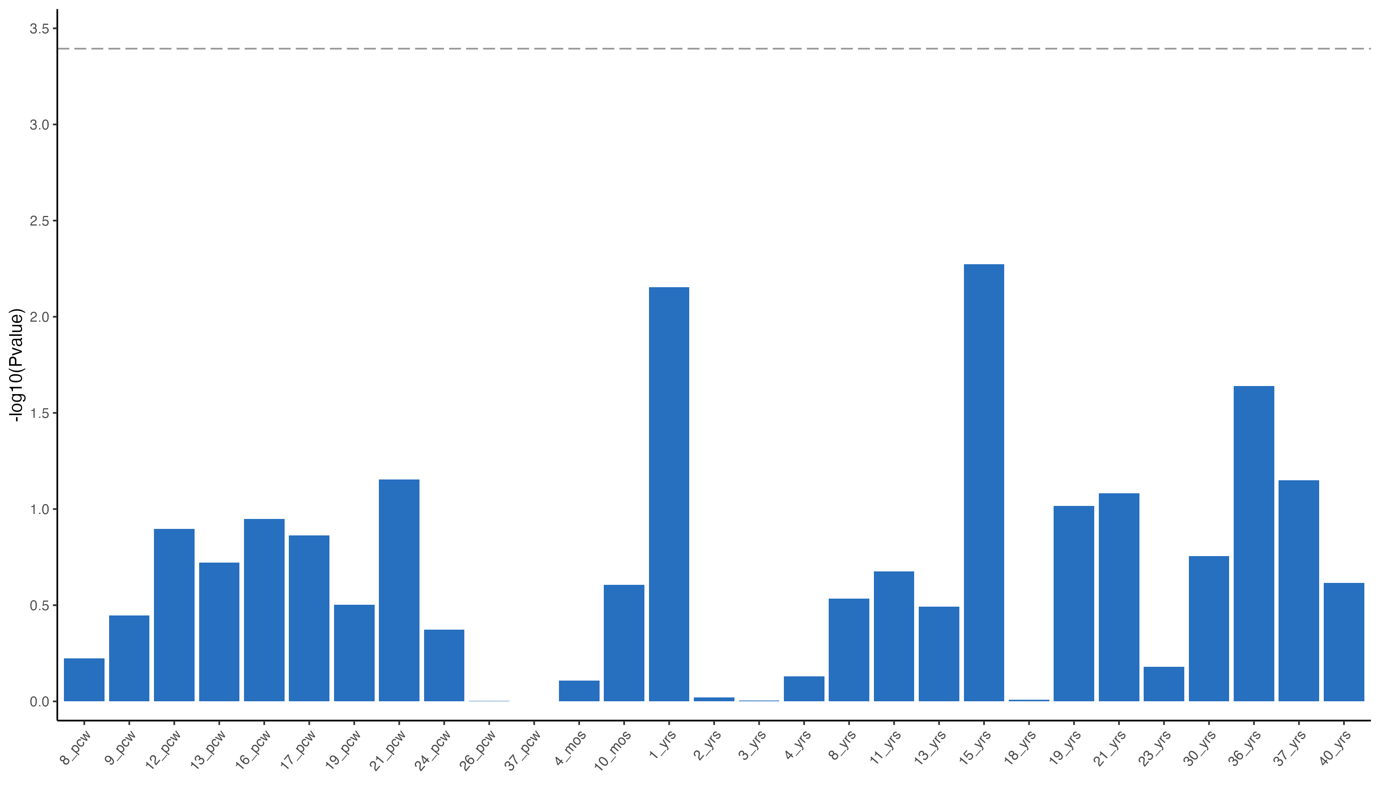


**Figure S43:** -log_10_ P values from a MAGMA gene property analysis of dyslexia multivariate GWAS showing enrichment through 29 human brain ages from BrainSpan. The dashed line indicates the Bonferroni correction for 124 tests to P < 4.03 x 10^-4^. Yrs = years, pcw = post conception weeks.


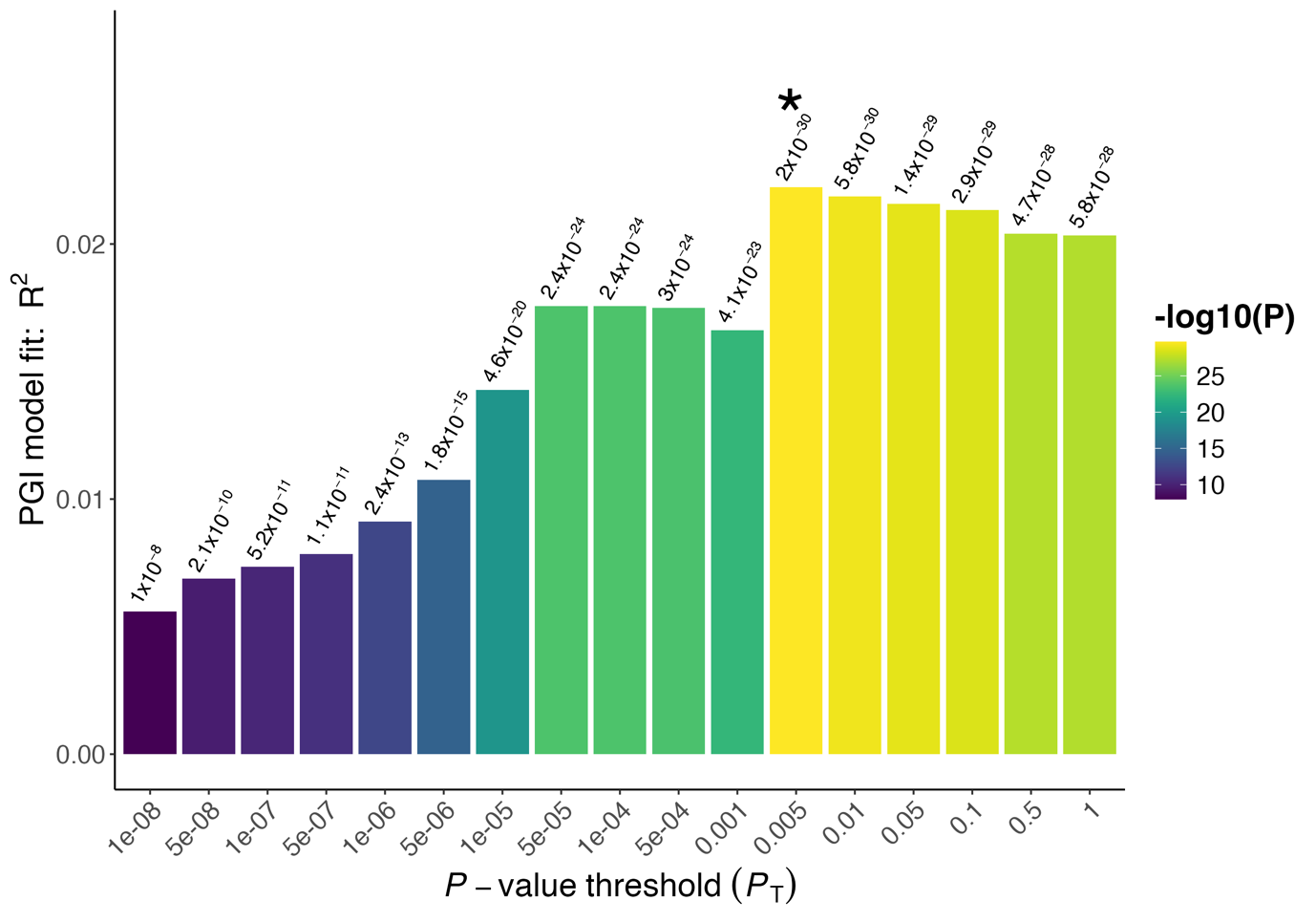


**Figure S44:** Threshold model fit for polygenic index of dyslexia using PRSice2, predicting a composite measure of reading ability at age 7 in the NCDS cohort. X axis indicated the range of P value cutoffs tested, and Y axis shows the PGI model fit (*R^2^*) at each threshold. * indicates the best P value threshold for each PGI model.


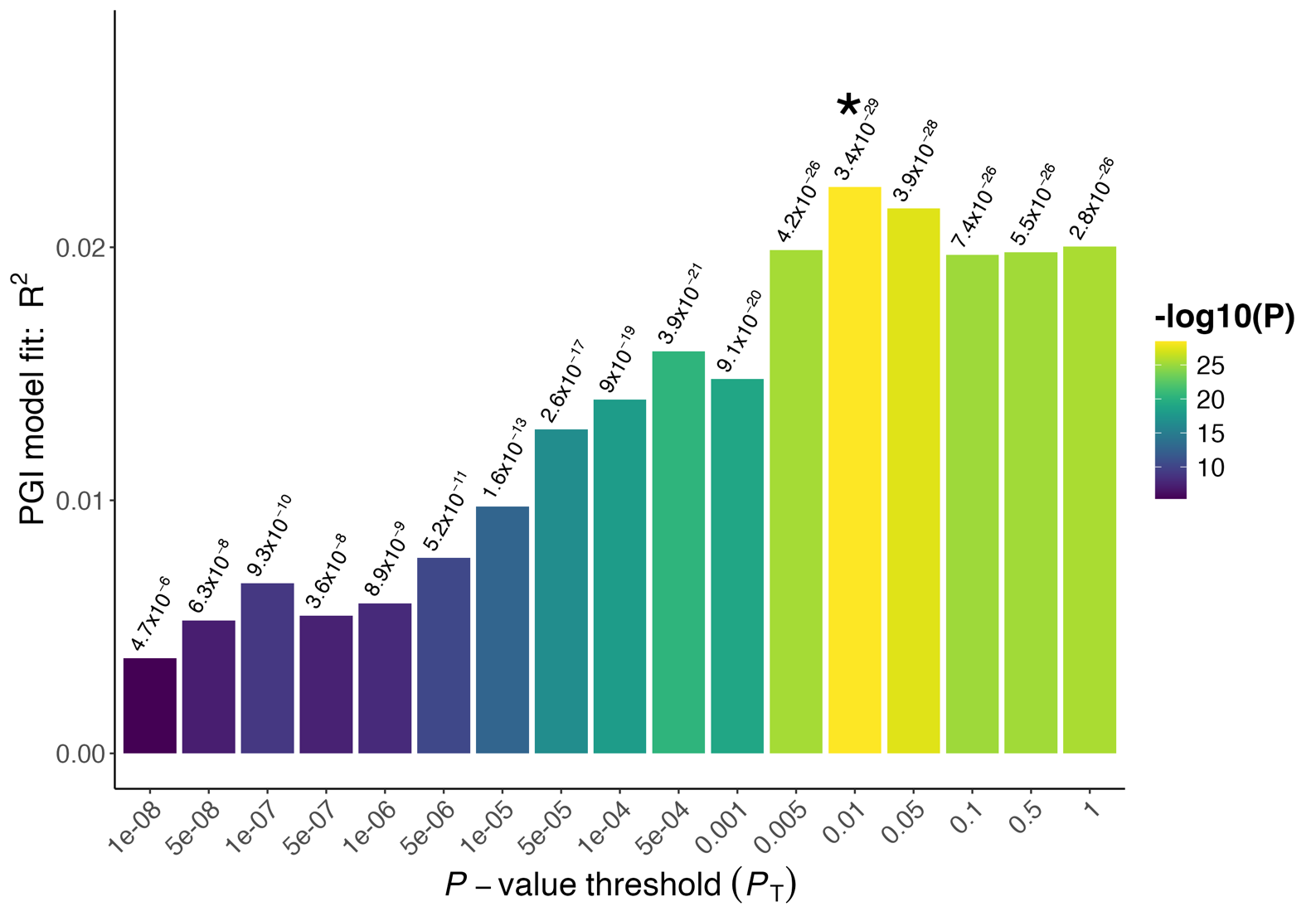


**Figure S45:** Threshold model fit for polygenic index of dyslexia using PRSice2, predicting a composite measure of reading ability at age 11 in the NCDS cohort. X axis indicated the range of P value cutoffs tested, and Y axis shows the PGI model fit (*R^2^*) at each threshold. * indicates the best P value threshold for each PGI model.


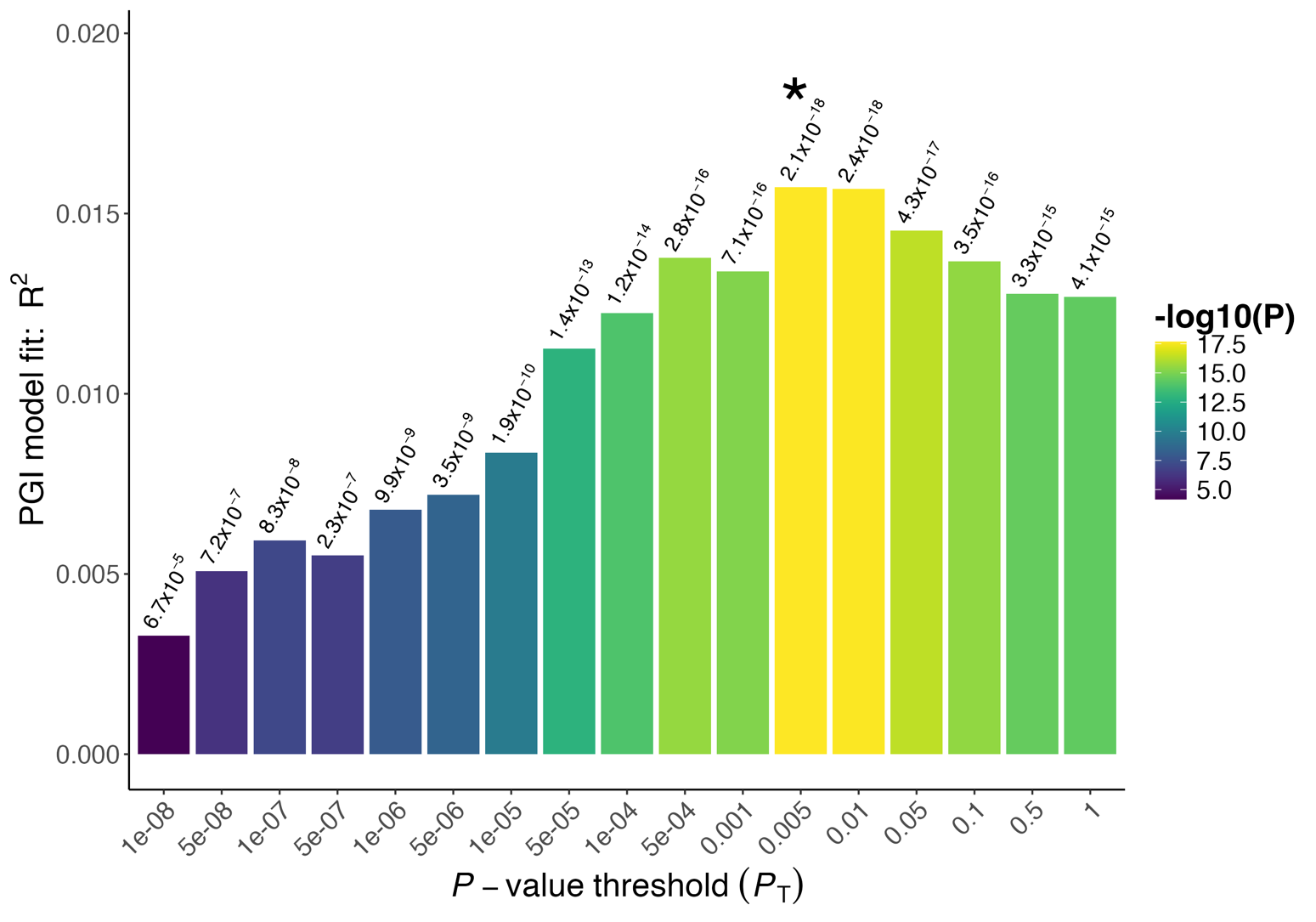


**Figure S46:** Threshold model fit for polygenic index of dyslexia using PRSice2, predicting a composite measure of reading ability at age 16 in the NCDS cohort. X axis indicated the range of P value cutoffs tested, and Y axis shows the PGI model fit (*R^2^*) at each threshold. * indicates the best P value threshold for each PGI model.

**
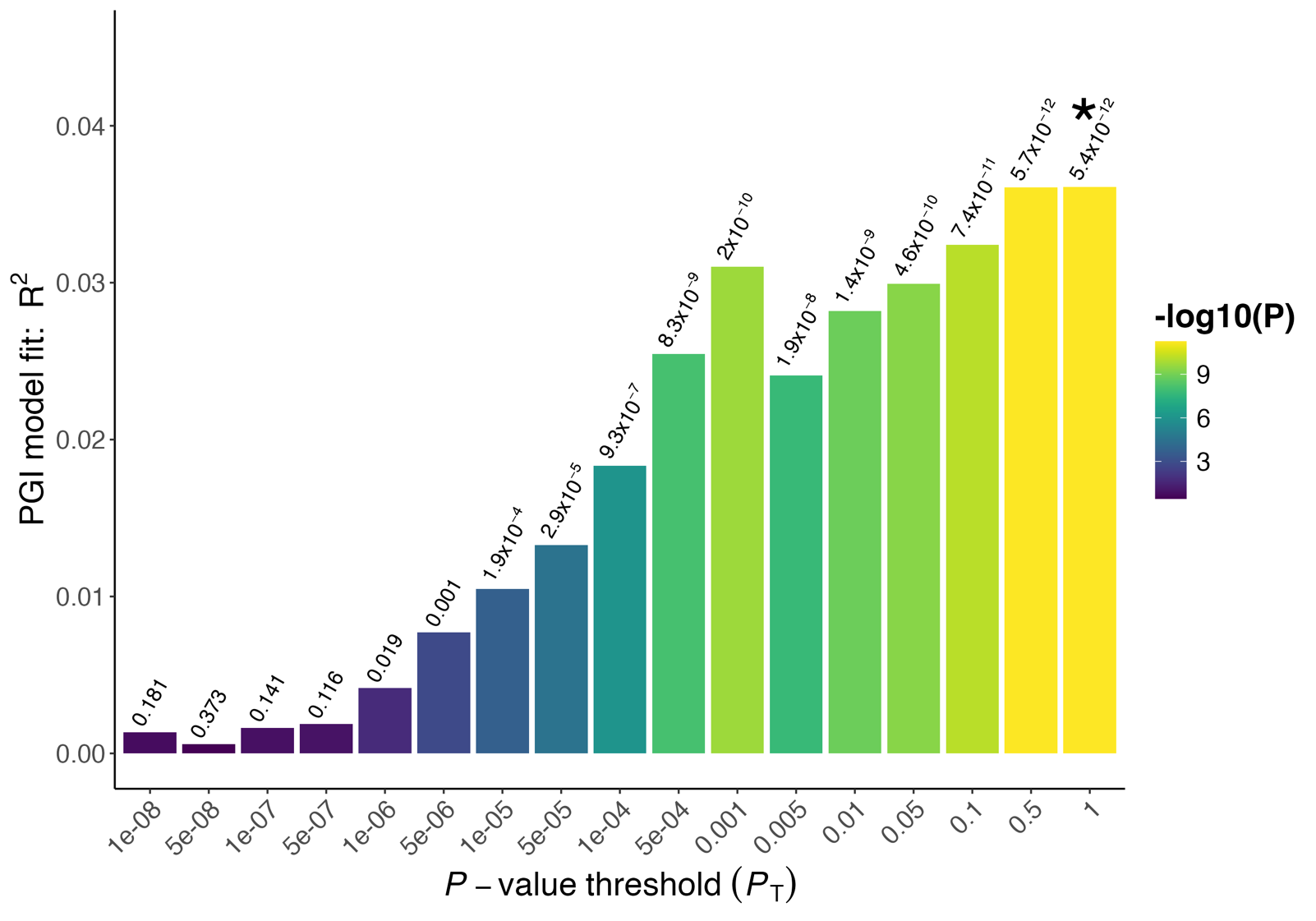
**

**Figure S47:** Threshold model fit for polygenic index of dyslexia using PRSice2, predicting a binary measure reading ability at age 23 in the NCDS cohort. X axis indicated the range of P value cutoffs tested, and Y axis shows the PGI model fit (*R^2^*) at each threshold. * indicates the best P value threshold for each PGI model.

**
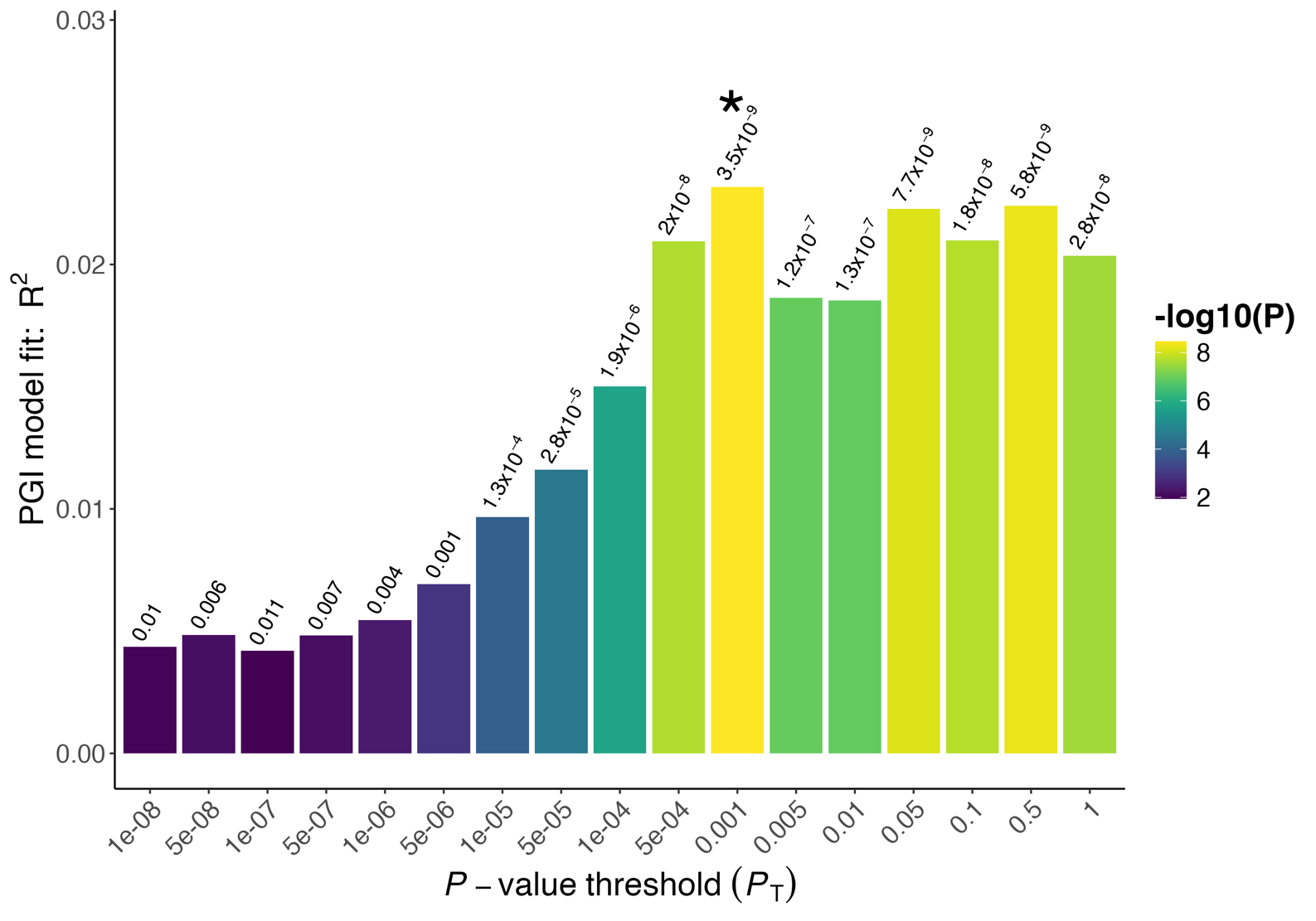
**

**Figure S48:** Threshold model fit for polygenic index of dyslexia using PRSice2, predicting a binary measure of reading ability at age 33 in the NCDS cohort. X axis indicated the range of P value cutoffs tested, and Y axis shows the PGI model fit (*R^2^*) at each threshold. * indicates the best P value threshold for each PGI model.

**
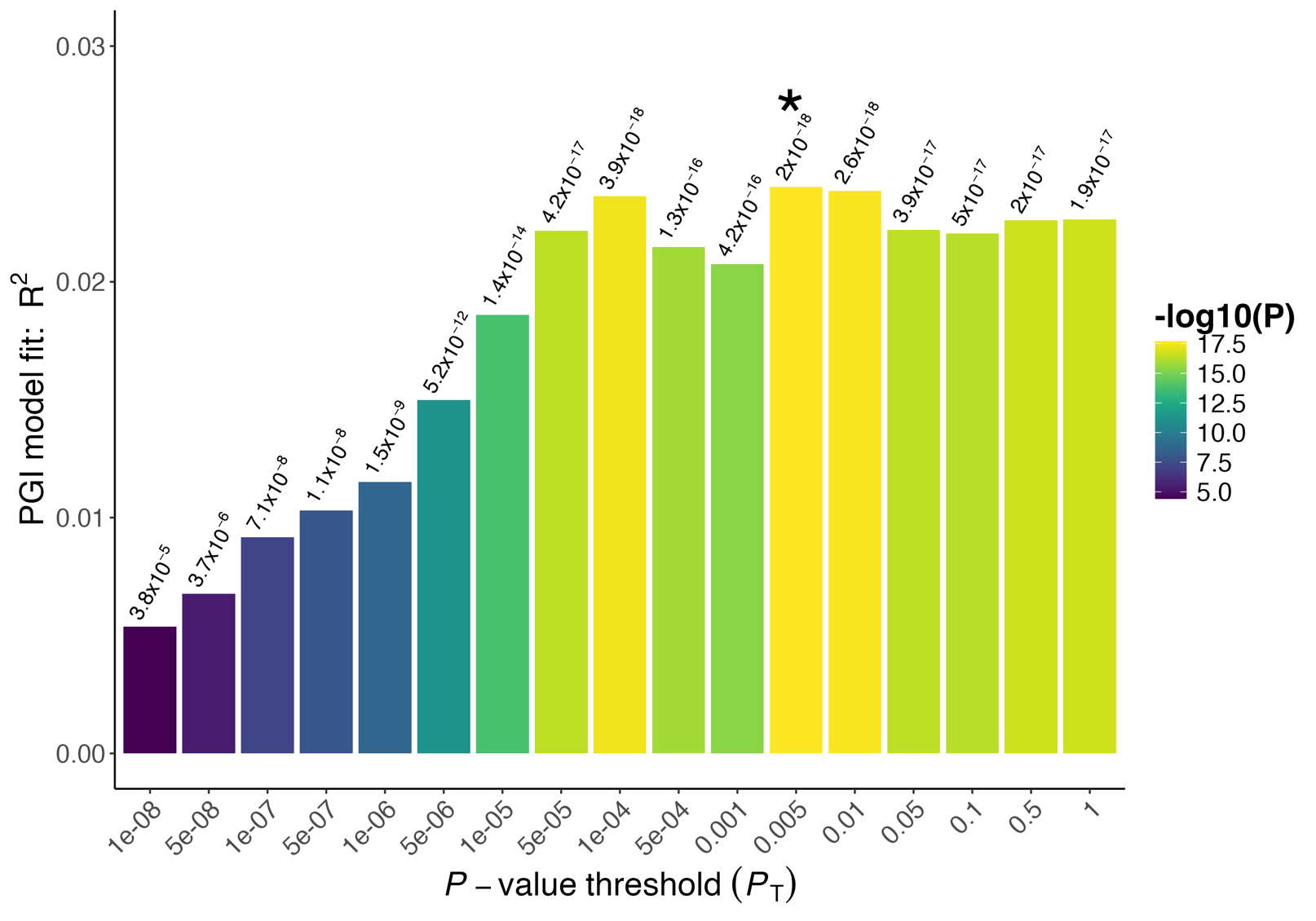
**

**Figure S49:** Threshold model fit for polygenic index of dyslexia using PRSice2, predicting a composite measure of reading ability across all ages in the NCDS cohort. X axis indicated the range of P value cutoffs tested, and Y axis shows the PGI model fit (*R^2^*) at each threshold. * indicates the best P value threshold for each PGI model.

**Supplementary Tables Text**

**Table S1**: SNPs which were most strongly associated (P ≤ 1 x 10^-5^, N = 18,551) with multivariate analysis of dyslexia.

**Table S2**: SNPs which were most strongly associated (P ≤ 1 x 10^-5^, N = 9,717) with multivariate analysis of reading ability.

**Table S3:** Regions significantly associated with multivariate GWAS of reading ability. Regions are described as previously reported as significant in Doust et. al. 2023, significant in the uncorrected summary statistics, or novel ^2^.

**Table S4:** Complete results for all 2824 genetic correlations tested in LDSC.

**Table S5:** Gene-based association results tested for 18,842 genes using MAGMA for multivariate dyslexia.

**Table S6:** Gene-set association results for 9,113 biological pathways using MAGMA for multivariate dyslexia.

**Table S7:** Variant Effect Predictions for coding SNPs. Prioritised variants annotated as damaging by both SIFT and PolyPhen2 (N = 9) are presented in bold.

**Table S8:** Gene-based annotations using FUMA showing loss-of-function predictions, and expression QTL associations.

**Table S9:** MAGMA gene-property analysis of multivariate reading ability partitioned by GTEx gene expression in individual tissues.

**Table S10:** MAGMA gene-property analysis partitioned by brain tissue gene expression across 11 developmental stages in BrainSpan.

**Table S11:** MAGMA gene-property analysis partitioned by brain tissue gene expression across 29 ages in BrainSpan.

**Table S12:** MAGMA gene-property analysis partitioned by single-cell RNA-seq brain tissue gene expression in embryonic ventral mid-brain.

**Table S13:** MAGMA gene-property analysis partitioned by single-cell RNA-seq brain tissue gene expression in embryonic pre-frontal cortex.

**Table S14:** MAGMA gene-property analysis partitioned by single-cell RNA-seq brain tissue gene expression in adult and foetal cortex grouped by neuronal cell type.

**Table S15:** Results of LDSC partitioning heritability in tissue-specific chromatin modification patterns from the Roadmap Epigenomics project and ENTEX, using the annotations and method of Finucane et al. 2015 ^11^.

**Table S16:** Polygenic index prediction for dyslexia multivariate summary statistics using PRSice2 and SBayesRC, across six measures of longitudinal reading ability in the National Child Development Study, 1958.

**Table S17:** Table showing polygenic selection analysis results for 104 independent SNPs associated with dyslexia in an imputed ancient panel.
